# Synthesis and Herbicidal Activity of Novel 1-(Diethoxy-phosphoryl)-3-(4-one-1*H*-1,2,3-triazol-1-yl)-propan-2-yl Carboxylic Esters

**DOI:** 10.3390/molecules20011088

**Published:** 2015-01-12

**Authors:** Yan Jin, Hanqing Zhao, Huizhe Lu, Colleen M. Kuemmel, Jianjun Zhang, Daoquan Wang

**Affiliations:** 1 Department of Applied Chemistry, China Agricultural University, Beijing 100193, China; E-Mails: walterjin@126.com (Y.J.); zhaohanqing@bua.edu.cn (H.Z.); wangdq@cau.edu.cn (D.W.); 2 Department of Fundamental Science, Beijing University of Agriculture, Beijing 102206, China; 3 Novo Nordisk, Inc., Princeton, NJ 08540, USA; E-Mail: colleenkuemmel@gmail.com

**Keywords:** carboxylic esters, IGPD, synthesis, herbicidal activity

## Abstract

A series of novel compounds, namely 1-(diethoxyphosphoryl)-3-(4-ones-1*H*-1,2,3-triazol-1-yl)propan-2-yl carboxylic esters, were designed on the basis of the diazafulvene intermediate of imidazole glycerol phosphate dehydratase (IGPD) and high-activity inhibitors of IGPD, and synthesized as inhibitors targeting IGPD in plants. Their structures were confirmed by ^1^H-NMR, ^13^C-NMR, ^31^P-NMR and HR-MS. The herbicidal evaluation performed by a Petri dish culture method showed that most compounds possessed moderate to good herbicidal activities. Six compounds were chosen for further herbicidal evaluation on barnyard grass by pot experiments. 1-(Diethoxyphosphoryl)-3-(4-phenyl-1*H*-1,2,3-triazol-1-yl)propan-2-yl 2-(naphthalen-1-yl)acetate (**5-A3**) and ethyl 1-(2-acetoxy-3-(diethoxyphosphoryl)propyl)-1*H*-1,2,3-triazole-4-carboxylate (**5-B4**) showed good herbicidal activities. Compared with the compounds with the best herbicidal activity ever reported, both compounds **5-A3** and **5-B4**, which can inhibit the growth of barnyard grass at the concentration of 250g/hm^2^, efficiently gave rise to a nearly 4-fold increase of the herbicidal potency. However, their herbicidal activities were lower than that of acetochlor (62.5 g/hm^2^) in the pot experiments.

## 1. Introduction

Imidazole glycerol phosphate dehydratase (IGPD) was recently discovered as a new potential target enzyme for the development of herbicides. It catalyzes the conversion of imidazole glycerol phosphate (IGP) to imidazole acetol phosphate (IAP) in the histidine biosynthetic pathway [1]. Since IGPD is absent from mammals, its inhibition provides a safe and effective method for controlling weed growth [2].

Many high-activity inhibitors of IGPD such as (3-hydroxy-3-(1*H*-1,2,4-triazol-3-yl)propyl) phosphonic acid (IRL1695, Mori *et al*. [3]) and (2-(3-phenoxypropanamido)-3-(1*H*-1,2,4-triazol-1-yl)propyl) phosphonic acid (Schweitzer *et al*. [4]) were recently synthesized [5]. While these inhibitors exhibited excellent enzymatic inhibitor activity, only the compound IRL1803 [3-hydroxy-3-(1*H*-[1,2,4]triazol-3-yl)-cyclohexyl]-phosphonic acid (Mori *et al.* [6]) also elicited herbicidal activity, preventing weed growth at a concentration of 1 kg/hm^2^. Although many inhibitors of IGPD were reported, the mechanism of the IGP dehydratase reaction is still unknown.

## 2. Results and Discussion

### 2.1. Design of the Target Molecules

IRL1695 was the first high-activity inhibitor of IGPD (IC_50_ = 130 nM, pH = 7.5, K_i_ = 40 ± 6.5 nM, K_m_/K_i_ = 9.0 × 10^3^). This compound was designed on the basis of the diazafulvene intermediate of IGPD [3,7]. Then a series of novel β-carboxamido phosphonate compounds were synthesized, most of which provided (Schweitzer *et al*. [8]) moderate to good enzymatic inhibitory activity. One in particular, phenoxyacetamide **5a**, displayed an inhibition constant of 80 nM. Schweitzer suggested that the phenyl ring of â-carboxamido phosphates may contribute to a putative lipophilic interaction between the inhibitor and IGPD. In this scenario, the phenyl ring may provide greater inhibition by filling the enzymatic cavity better than five-membered rings or aliphatic groups. Alternatively, the phenyl ring may be involved in a π-π interaction with an aromatic residue of the protein.

**Figure 1:**
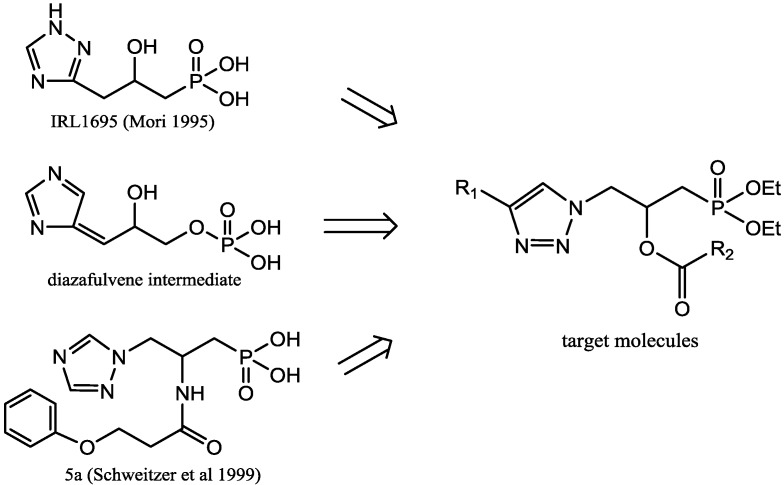
The design of the target molecules.

Inspired by these promising results, we decided to search for potential herbicidal activity in triazole derivatives. By taking advantage of these active substructures and the diazafulvene intermediate of IGPD [9], a series of novel 1-(diethoxyphosphoryl)-3-(4-one-1*H*-1,2,3-triazol-1-yl)propan-2-yl carboxylic ester compounds (Figure 1) were designed and synthesized. In the structure of compound **5**, the triazole moiety and the methyldiethoxyphosphine were connected via a substituted alkyl. In order to increase the osmosis of compounds into weeds, the phosphate of the diazafulvene intermediate and the methanephosphonic acid of IRL1695 and **5a** (Schweitzer *et al*. [8]) were replaced by methyldiethoxyphosphine. The *in vivo* herbicidal activity of all the target compounds was also evaluated. We report herein the preliminary results of these studies.

### 2.2. Synthesis of the Title Compounds

**Scheme 1:**
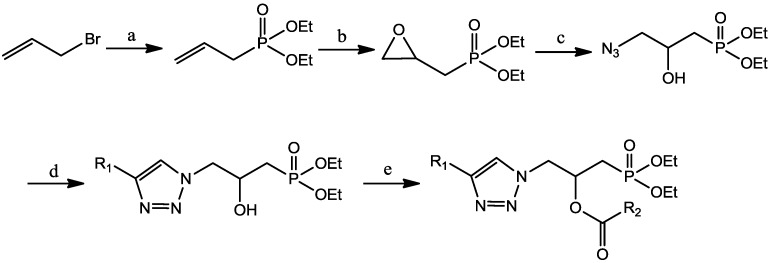
Synthesis of the target compounds **5**.

The design of the target molecules was based on some highly active substructures and the diazafulvene intermediate of IGPD [4] (Scheme 1).

*Reagents and conditions*: (a) (EtO)_3_P, reflux., 4 h, 98% for **1**; (b) *m*-CPBA, CH_2_Cl_2_, r.t, overnight 80% for **2**; (c) NH_4_Cl, NaN_3_, CH_3_OH-H_2_O, r.t, overnight; (d) sodium ascorbate, CuSO_4_^.^5H_2_O, alkyne, CH_3_OH-H_2_O, r.t, overnight, 60%–80% for 4; (e) DCC, CH_2_Cl_2_, reflux 8–14 h, 34%–80% for 5.

All target compounds were synthesized via a five-step procedure (Scheme 1). The key intermediate, diethyl(2-hydroxy-3-(4-one-1*H*-1,2,3-triazol-1-yl)propyl)phosphonate (**4**), was prepared as previously described [10,11,12,13]. According to previous studies, compound **3** does not need to be purified. Thus, the mixture from the third step was used to prepare compound **4** directly. Compound **4** was allowed to react at room temperature with various carboxylic acids in the presence of DCC and DMAP as catalysts to give the target compounds **5** in 34%–80% yields.. At first, carboxylic acids were converted to acyl chlorides by oxalyl chloride, then reacted with compound **4** to give the target compounds. However, the reactions gave low yields and the target compounds were difficult to separate. Their structures were confirmed by ^1^H-NMR which showed the characteristic signals such as a single peak at about δ 8.03 ppm for C-CH-N of 5A, ^13^C-NMR, ^31^P-NMR and HR-MS.

### 2.3. Herbicidal Activity of Compounds **5**

The herbicidal evaluation was performed using wheat and rape as model plants by the reported Petri dish culture method [14]. Most compounds were found to possess moderate to good herbicidal activities. Compounds **5-A3**, **5-B3**, **5-B4**, **5-C2**, **5-C3** and **5-D3** were screened further by using a pot experiment with barnyard grass. Of these, 1-(diethoxyphosphoryl)-3-(4-phenyl-1*H*-1,2,3-triazol-1-yl)propan-2-yl 2-(naphthalen-1-yl)acetate (**5-A3**) and ethyl 1-(2-acetoxy-3-(diethoxyphosphoryl)-propyl)-1*H*-1,2,3-triazole-4-carboxylate (**5-B4**) showed good herbicidal activity. However, their herbicidal activities were lower than that of acetochlor. The herbicidal evaluation on barnyard grass had two parts, pre-emergence treatment and post-emergence treatment, which is described in the Experimental Section. The activity data are listed in Table 1 and Table 2.

**Table 1:** The percent inhibitory ratios against the growth of root and stalk of wheat and rape of **5** at different concentrations. 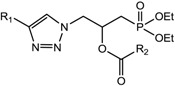

Comp.	R_1_	R_2_	Relative Inhibition (Root %/Stalk %)			
			Rape Wheat			
			100 mg/L	10 mg/L	100 mg/L	10 mg/L
**5-A1**	C_6_H_5_-	C_6_H_5_-	87.4/61.3	30.3/7.1	73.4/63.1	24.3/23.3
**5-A2**	C_6_H_5_-	4-NO_2_C_6_H_4_-	79.2/61.6	20.3/11.4	78.5/74.5	39.2/12.8
**5-A3**	C_6_H_5_-	α-C_10_H_7_CH_2_-	92.5/75.7	84.6/64.5	69.6/53.6	56.9/30.9
**5-A4**	C_6_H_5_-	CH_3_-	84.0/43.8	41.7/11.6	66.2/53.7	37.0/16.0
**5-A5**	C_6_H_5_-	2-FC_6_H_4_-	89.0/75.0	59.8/25.1	71.8/51.7	36.5/14.0
**5-A6**	C_6_H_5_-	2-ClPy-3-	90.2/64.0	34.5/14.1	78.4/70.7	32.4/16.6
**5-A7**	C_6_H_5_-	4-OCH_3_C_6_H_4_-	84.4/57.5	34.3/18.1	76.8/67.3	24.5/12.2
**5-A8**	C_6_H_5_-	3,5-(NO_2_)_2_C_6_H_3_-	69.5/21.6	26.5/0.0	71.3/56.2	51.3/37.4
**5-B1**	EtOOC-	C_6_H_5_-	84.2/61.6	43.5/25.4	75.2/58.5	49.7/28.7
**5-B2**	EtOOC-	4-NO_2_C_6_H_4_-	84.3/71.2	51.0/7.4	85.6/72.6	49.7/47.9
**5-B3**	EtOOC-	α-C_10_H_7_CH_2_-	97.7/82.5	93.0/68.8	95.8/86.7	85.0/51.0
**5-B4**	EtOOC-	CH_3_-	92.3/75.5	43.0/18.9	83.9/77.3	56.9/40.3
**5-B5**	EtOOC-	2-FC_6_H_4_-	77.7/50.7	34.3/7.4	78.3/72.5	44.4/30.1
**5-B6**	EtOOC-	2-ClPy-3-	75.5/57.8	25.5/11.2	64.6/44.3	5.2/0.0
**5-B7**	EtOOC-	4-OCH_3_C_6_H_4_-	62.3/21.9	43.7/7.5	61.8/47.4	12.1/7.4
**5-B8**	EtOOC-	3,5-(NO_2_)_2_C_6_H_3_-	70.6/29.5	18.6/-11.8	73.2/67.2	27.1/5.1
**5-C1**	t-Bu	C_6_H_5_-	89.9/64.7	56.6/32.2	66.8/47.7	32.1/14.1
**5-C2**	t-Bu	4-NO_2_C_6_H_4_-	70.2/25.4	26.8/11.8	86.3/77.7	53.6/42.8
**5-C3**	t-Bu	α-C_10_H_7_CH_2_-	95.8/82.8	92.7/64.8	90.7/77.0	75.4/37.4
**5-C4**	t-Bu	CH_3_-	46.3/21.3	18.5/-7.2	71.3/67.0	32.3/16.1
**5-C5**	t-Bu	2-FC_6_H_4_-	75.4/46.5	26.5/0.0	75.7/70.3	29.9/14.9
**5-C6**	t-Bu	4-OCH_3_C_6_H_4_-	84.0/64.5	30.9/18.9	59.2/51.5	24.7/7.1
**5-C7**	t-Bu	3,5-(NO_2_)_2_C_6_H_3_-	70.0/36.3	48.8/14.8	58.6/44.2	32.8/30.5
**5-D1**	ClCH_2_-	C_6_H_5_-	75.3/57.6	25.6/29.4	68.7/63.4	51.5/40.1
**5-D2**	ClCH_2_-	4-NO_2_C_6_H_4_-	74.4/50.7	48.3/32.2	76.8/72.8	22.7/2.1
**5-D3**	ClCH_2_-	α-C_10_H_7_CH_2_-	97.6/86.8	93.4/57.8	92.1/77.0	36.4/9.8
**5-D4**	ClCH_2_-	CH_3_-	77.3/50.5	28.5/18.8	66.5/60.9	22.9/5.0
**5-D5**	ClCH_2_-	2-FC_6_H_4_-	64.4/29.6	31.3/7.9	66.3/53.8	20.9/9.2
**5-D6**	ClCH_2_-	4-OCH_3_C_6_H_4_-	82.3/61.7	28.7/-4.8	63.6/56.2	36.0/19.5
**5-D7**	ClCH_2_-	3,5-(NO_2_)_2_C_6_H_3_-	72.2/32.2	34.8/21.3	71.6/53.8	7.0/7.2

**Table 2:** The fresh weight reduction ratios of Barnyard grass at different concentrations of **5**.

Comp.	Fresh Weight Reduction Ratios of Barnyard Grass (%)							
	Pre-Emergence Treatment				Post-Emergence Treatment			
	4 kg/hm^2^	1 kg/hm^2^	250 g/hm^2^	62.5 g/hm^2^	4 kg/hm^2^	1 kg/hm^2^	250 g/hm^2^	62.5 g/hm^2^
**Acetochlor**				99.0				99.0
**5-A3**	40.4	13.3	4.0	−2.5	99.3	93.1	79.1	68.3
**5-B3**	51.4	30.5	11.0	4.2	68 .1	47.1	15.7	4.6
**5-B4**	34.3	11.8	6.2	4.8	97.6	88.1	74.8	30.7
**5-C2**	28.7	19.0	0.0	−4.1	55.1	30.9	4.1	0.0
**5-C3**	49.8	21.4	2.2	2.7	81.0	72.0	55.5	23.1
**5-D3**	26.9	13.3	0.0	2.1	64.1	38.4	11.0	11.4

The preliminary bioassay results showed that most compounds **5** possess moderate to good herbicidal activity. When evaluating the inhibitory activity of compounds **5** at a concentration of 100 mg/L against the root growth of wheat, compounds **5-B3**, **5-C3** and **5-D3** exhibited more than 90% inhibitory activity and 20 compounds exhibited more than 70% inhibitory activity. At 10 mg/L, compounds **5-B3** and **5-C3** exhibited more than 70% inhibitory activity against the root growth of wheat. When evaluating the inhibitory activity of compounds **5** at 100 mg/mL against the stalk growth of wheat, 11 compounds exhibited more than 70% inhibitory activity. At 10 mg/L, all compounds **5** exhibited slight inhibitory activity against the stalk growth of wheat, while compound **5-B3** was the best, exhibiting 51% inhibitory activity.

**Figure 2:**
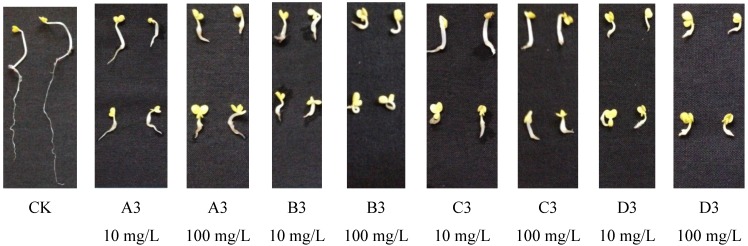
Photographs showing the lateral root and stalk development of dicotyledon rape with compounds **5-A3**, **5-B3**, **5-C3**, **5-D3** at different concentrations.

According to the Figure 2, evaluation of the inhibitory activity of compounds **5** at 100 mg/L against the root growth of rape showed compounds **5-A3**, **5-A6**, **5-B3**, **5-B4**, **5-C3** and **5-D3** exhibited more than 90% inhibitory activity and 29 compounds exhibited more than 70% inhibitory activity. At 10 mg/L, compounds **5-B3**, **5-C3** and **5-D3** exhibited more than 90% inhibitory activity. Investigation of the inhibitory activities of compounds against the stalk growth of rape showed compounds **5-A3**, **5-A5**, **5-B2**, **5-B3**, **5-B4**, **5-C3** and **5-D3** exhibited more than 70% inhibitory activity at 100 mg/L, while at 10 mg/L, all the compounds **5** exhibited slight inhibitory activity against the stalk growth of rape. Interestingly, compounds **5-B3** also exhibited the best inhibitory activity against the stalk growth of rape among all compounds **5**.

Overall, the inhibitory activity against root growth was stronger than the inhibitory activity against stalk growth. Interestingly, compound **5-B3** exhibited the best inhibitory activity in both wheat and rape, even at the concentration of 10 mg/L, where inhibitory activity against root growth of rape was 93%. Aside from compound **5-B3**, compounds whose R_2_ is α-naphthylmethylene (**5-A3**, **5-B3**, **5-C3**, **5-D3**) also exhibited much better inhibitory activity than other target compounds. This observation builds on the conclusion of Schweitzer, where the naphthyl ring fills the volume of the enzymatic cavity even more efficiently than the phenyl ring. Alternatively, the π-π interaction of the naphthyl ring with an aromatic residue of the protein may be stronger than with the phenyl ring.

**Figure 3:**
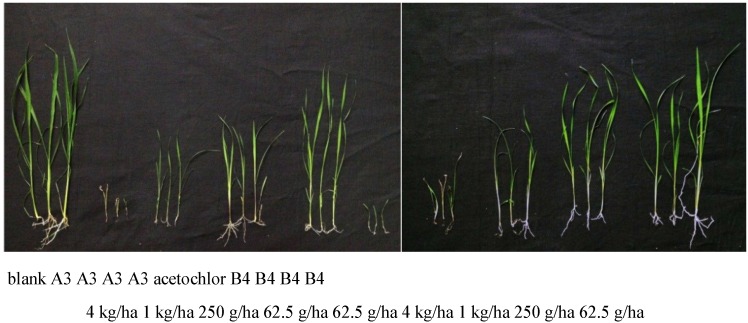
Photographs showing the lateral development of monocotyledon barnyard grass with compounds **5-A3**, **5-B4** at different concentrations with a pot experiment.

In the pot experiment, we chose six compounds (**5-A3**, **5-B3**, **5-B4**, **5-C2**, **5-C3**, **5-D3**) which exhibited more than 90% inhibitory activity against stalk growth or root growth. According to the Figure 3, the pot experiment on barnyard grass shows that compounds **5-A3** and **5-B4** exhibited good inhibitory activity with post-emergence treatment. But all the six compounds (**5-A3**, **5-B3**, **5-B4**, **5-C2**, **5-C3**, **5-D3**) only exhibited slight inhibitory activity in pre-emergence treatment. Compared with IRL1803, which exhibits the best herbicidal activity ever reported, compounds **5-A3** and **5-B4** both gave displayed a nearly a 4-fold increase in inhibition. These compounds can also efficiently inhibit the growth of barnyard grass at a concentration of 250 g/hm^2^. The increase of herbicidal activity maybe the result of the ester group in **5-A3** which increases the osmosis of compounds into weeds.

## 3. Experimental

### 3.1. General Methods

All starting materials and reagents were commercially available and used without further purification except as indicated. ^1^H-NMR (300 MHz), ^13^C-NMR (75.5 MHz) and ^31^P-NMR (121.5 MHz) spectra was recorded in CDCl_3_ or DMSO-*d*_6_ with a Bruker DPX300 spectrometer, using TMS as internal standard; Mass spectra were obtained with Agilent 1100 series LC/MSD mass spectrometer. High-resolution mass spectra (HRMS) was performed by the Peking University. Melting points were measured on a Yanagimoto melting-point apparatus and are uncorrected.

### 3.2. Chemical Synthesis

#### *General Procedure for the Synthesis of Compounds* **4** 

A mixture of diethyl (oxiran-2-ylmethyl)phosphonate (1.65 g, 10 mmol), sodium azide (1.30 g, 20 mmol) and ammonium chloride (1.06 g, 20 mmol) was dissolved in 1:1 mixture of methanol and water (30 mL), and stirred at room temperature overnight. Dichloromethane (50 mL) was added and then the organic phase was washed with water (3 × 50 mL). The organic phase was dried with anhydrous Na_2_SO_4_ and concentrated under vacuum. The residue was dissolved in a 1:1 mixture of methanol and water (30 mL), CuSO_4_·5H_2_O (0.16 g, 1 mmol) and sodium ascorbate (0.20 g, 1 mmol) and then alkyne (11 mmol) was added. The mixture was stirred at room temperature overnight. Dichloromethane was then added and the mixture was washed with water, dried with Na_2_SO_4_, and concentrated by vacuum. The desired product was obtained by purification on a silica gel column with petroleum ether–ethyl acetate.

*Diethyl (2-hydroxy-3-(4-phenyl-1H-1,2,3-triazol-1-yl)propyl)phosphonate* (**4-A**). Yield: 89%. White solid. ^1^H-NMR (CDCl_3_) δ 1.26 (t, *J* = 7.1 Hz, 6H), 1.95–2.05 (m, 2H), 3.98–4.10 (m, 4H), 4.38–4.49 (m, 2H), 4.57–4.61 (m, 1H), 5.37 (d, *J =* 4.4Hz, 1H), 7.26–7.39 (m, 3H), 7.76–7.79 (m, 2H), 8.03 (S, 1H); ^13^C-NMR (CDCl_3_) δ 15.90 (d, *J*_C-P_ = 6.2 Hz), 30.51 (d, *J*_C-P_ = 140.0 Hz), 55.70 (d, *J*_C-P_ = 15.5 Hz), 61.60 (d, *J*_C-P_ = 6.4 Hz), 61.79 (d, *J*_C-P_ = 6.3 Hz), 65.07 (d, *J*_C-P_ = 3.2 Hz), 121.25, 125.18, 127.62, 128.38, 130.18, 146.92; ^31^P-NMR (CDCl_3_) δ 29.04. 

*Ethyl 1-(3-(diethoxyphosphoryl)-2-hydroxypropyl)-1H-1,2,3-triazole-4-carboxylate* (**4-B**). Yield: 79%. White solid. ^1^H-NMR (CDCl_3_) δ 1.30–1.43 (m, 9H), 1.78–1.87 (m, 1H), 1.95–2.08 (m, 1H), 4.05–4.17 (m, 4H), 4.39–4.50 (m, 4H), 4.59–4.64 (m, 1H), 8.31 (s, 1H); ^13^C-NMR (CDCl_3_) δ 14.23, 16.24 (d, *J*_C-P_ = 3.4 Hz), 16.32 (d, *J*_C-P_ = 3.3 Hz), 30.47 (d, *J*_C-P_ = 140.7 Hz), 55.99 (d, *J*_C-P_ = 18.3 Hz), 61.19, 62.27 (d, *J*_C-P_ = 6.8 Hz), 62.39 (d, *J*_C-P_ = 6.6 Hz), 65.17 (d, *J*_C-P_ = 4.1 Hz), 129.16, 140.07, 160.68; ^31^P-NMR (CDCl_3_) δ 29.03.

*Diethyl (3-(4-(tert-butyl)-1H-1,2,3-triazol-1-yl)-2-hydroxypropyl)phosphonate* (**4-C**). Yield: 69%. White solid. ^1^H-NMR (CDCl_3_) δ 1.26–1.35 (m, 15H), 1.84–2.09 (m, 2H), 4.05–4.16 (m, 4H), 4.36–4.45 (m, 2H), 4.53–4.57 (m, 1H), 7.51 (s, 1H) ; ^13^C-NMR (CDCl_3_) δ 16.10 (d, *J*_C-P_ = 2.5 Hz), 16.19 (d, *J*_C-P_ = 2.3 Hz), 30.14, 30.48, 30.60 (d, *J*_C-P_ = 140.0 Hz), 55.51 (d, *J*_C-P_ = 16.1 Hz), 61.94 (d, *J*_C-P_ = 6.9 Hz), 62.03 (d, *J*_C-P_ = 7.3 Hz), 65.41 (d, *J*_C-P_ = 3.6 Hz), 120.20, 157.21; ^31^P-NMR (CDCl_3_) δ 29.09.

*Diethyl (3-(4-(chloromethyl)-1H-1,2,3-triazol-1-yl)-2-hydroxypropyl)phosphonate* (**4-D**). Yield: 74%. White solid. ^1^H-NMR (CDCl_3_) δ 1.20–1.25 (m, 6H), 1.81–2.03 (m, 2H), 3.95–4.07 (m, 4H), 4.31–4.38 (m, 2H), 4.49–4.56 (m, 1H), 4.61 (s, 2H), 5.20 (d, *J =* 6.9 Hz, 1H), 7.83 (s, 1H); ^13^C-NMR (CDCl_3_) δ 15.75 (d, *J*_C-P_ = 1.2 Hz), 15.83 (d, *J*_C-P_ = 1.1 Hz), 30.36 (d, *J*_C-P_ = 139.8 Hz), 35.59, 55.50 (d, *J*_C-P_ = 14.9 Hz), 61.55 (d, *J*_C-P_ = 6.6 Hz), 61.70 (d, *J*_C-P_ = 6.4 Hz), 64.78 (d, *J*_C-P_ = 3.1 Hz), 124.17, 143.51; ^31^P-NMR (CDCl_3_) δ 28.61. 

#### *General Procedure for the Synthesis of Compounds* **5** 

A mixture of carboxylic acid (5 mmol) and DCC (1.07 g, 5.5 mmol) was dissolved in dichloromethane (40 mL), stirred at room temperature for 1 h. Then the mixture was placed in ice-bath and DMAP (0.06 g, 0.5 mmol), compounds **4** (4 mmol) was added, stirred for 10 min. Then the mixture was stirred at room temperature overnight. The desired product was obtained by purification on a silica gel column with petroleum ether/ethyl acetate.

*1-(Diethoxyphosphoryl)-3-(4-phenyl-1H-1,2,3-triazol-1-yl)propan-2-yl benzoate* (**5-A1**). Yield: 79%. White solid. ^1^H-NMR (CDCl_3_) δ 1.24 (t, *J =* 7.1 Hz, 3H), 1.30 (t, *J =* 7.1 Hz, 3H), 2.25–2.34 (m, 2H), 4.05–4.18 (m, 4H), 4.91–4.93 (m, 2H), 5.72–5.79 (m, 1H), 7.27–7.44 (m, 5H), 7.52–7.55 (m, 1H), 7.77–7.80 (m, 2H), 7.98 (s, 1H), 8.00–8.03 (m, 2H); ^13^C-NMR (CDCl_3_) δ 15.77 (d, *J*_C-P_ = 6.0 Hz), 15.85 (d, *J*_C-P_ = 6.0 Hz), 27.69 (d, *J*_C-P_ = 140.9 Hz), 52.14 (d, *J*_C-P_ = 7.9 Hz), 61.68 (d, *J*_C-P_ = 1.2 Hz), 61.76 (d, *J*_C-P_ = 1.7 Hz), 67.64, 120.75, 125.18, 127.65, 128.04, 128.32, 128.76, 129.23, 130.01, 133.03, 147.22, 164.74; ^31^P-NMR (CDCl_3_) δ 25.53; HR-MS: for C_22_H_26_N_3_O_5_P [M+H]^+^ calculated *m/z* 444.16, found *m/z* 444.1682839.

*1-(Diethoxyphosphoryl)-3-(4-phenyl-1H-1,2,3-triazol-1-yl)propan-2-yl 4-nitrobenzoate* (**5-A2**). Yield: 80%. White solid. ^1^H-NMR (CDCl_3_) δ 1.28 (t, *J =* 7.1 Hz, 3H), 1.34 (t, *J =* 7.0 Hz, 3H), 2.28 (d, *J =* 6.7 Hz, 1H), 2.34 (d, *J =* 6.8 Hz, 1H), 4.07–4.21 (m, 4H), 4.94–4.97 (m, 2H), 5.74–5.81 (m, 1H), 7.30–7.43 (m, 3H), 7.78–7.81 (m, 2H), 8.00 (S, 1H), 8.17–8.20 (m, 2H), 8.23–8.27 (m, 2H); ^13^C-NMR (CDCl_3_) δ 16.18 (d, *J*_C-P_ = 3.9 Hz), 16.26 (d, *J*_C-P_ = 4.1 Hz), 28.11 (d, *J*_C-P_ = 141.1 Hz), 52.28 (d, *J*_C-P_ = 7.2 Hz), 62.25 (d, *J*_C-P_ = 6.6 Hz), 68.93, 125.54, 128.18, 128.73, 130.12, 130.82, 134.49, 147.80, 150.65, 163.44; ^31^P-NMR (CDCl_3_) δ 25.06; HR-MS: for C_22_H_25_N_4_O_7_P [M+H]^+^ calculated *m/z* 489.15, found *m/z* 489.1533621.

*1-(Diethoxyphosphoryl)-3-(4-phenyl-1H-1,2,3-triazol-1-yl)propan-2-yl 2-(naphthalen-1-yl)acetate* (**5-A3**). Yield: 73%. White solid. ^1^H-NMR (CDCl_3_) δ 1.27 (t, *J =* 7.1 Hz, 6H), 1.98–2.10 (m, 2H), 4.01–4.11 (m, 6H), 4.55 (d, *J =* 4.6 Hz, 2H), 5.49–5.53 (m, 1H), 7.17 (s, 1H), 7.29–7.40 (m, 8H), 7.62–7.74 (m, 4H), 7.86–7.89 (m, 1H); ^13^C-NMR (CDCl_3_) δ 15.91 (d, *J*_C-P_ = 5.7 Hz), 27.58 (d, *J*_C-P_ = 141.0 Hz), 38.52, 52.04 (d, *J*_C-P_ = 8.1 Hz), 61.68 (d, *J*_C-P_ = 4.4 Hz), 61.77 (d, *J*_C-P_ = 4.3 Hz), 67.45, 120.22, 123.09, 124.96, 125.34, 125.44, 126.09, 127.61, 127.81, 128.21, 128.28, 129.33, 129.95, 131.42, 131.56, 133.21, 147.13, 169.69; ^31^P-NMR (CDCl_3_) δ 25.49; HR-MS: for C_27_H_30_N_3_O_5_P [M+H]^+^ calculated *m/z* 508.19, found *m/z* 508.199584.

*1-(Diethoxyphosphoryl)-3-(4-phenyl-1H-1,2,3-triazol-1-yl)propan-2-yl acetate* (**5-A4**). Yield: 71%. White solid. ^1^H-NMR (CDCl_3_) δ 1.34 (t, *J =* 7.1 Hz, 3H), 1.35 (t, *J =* 7.1 Hz, 3H), 2.06 (s, 3H), 2.08–2.13 (m, 1H), 2.15–2.19 (m, 1H), 4.09–4.19 (m, 4H), 4.69–4.85 (m, 2H), 5.46–5.53 (m, 1H), 7.31–7.36 (m, 1H), 7.40–7.45 (m, 2H), 7.82–7.85 (m, 2H), 7.92 (S, 1H); ^13^C-NMR (CDCl_3_) δ:15.90 (d, *J*_C-P_ = 6.1 Hz), 20.37, 27.72 (d, *J*_C-P_ = 141.1 Hz), 52.00 (d, *J*_C-P_ = 7.1 Hz), 61.71 (d, *J*_C-P_ = 1.1 Hz), 61.80 (d, *J*_C-P_ = 1.1 Hz), 66.99, 120.32, 125.24, 127.75, 128.36, 129.94, 147.37, 169.18; ^31^P-NMR (CDCl_3_) δ:25.61; HR-MS: for C_17_H_24_N_3_O_5_P [M+H]^+^ calculated *m/z* 382.15, found *m/z* 382.1526338.

*1-(Diethoxyphosphoryl)-3-(4-phenyl-1H-1,2,3-triazol-1-yl)propan-2-yl 2-fluorobenzoate* (**5-A5**). Yield: 73%. White solid. ^1^H-NMR (CDCl_3_) δ 1.26 (t, *J =* 7.2 Hz, 3H), 1.32 (t, *J =* 3.5 Hz, 3H), 2.24–2.34 (m, 2H), 4.06–4.19 (m, 4H), 4.93 (d, *J =* 5.2 Hz, 2H), 5.74–5.82 (m, 1H), 7.09–7.21 (m, 2H), 7.30–7.42 (m, 3H), 7.47–7.55 (m, 1H), 7.80–7.83 (m, 2H), 7.91–7.94 (m, 1H), 8.06 (S, 1H); ^13^C-NMR (CDCl_3_) δ:15.90 (d, *J*_C-P_ = 5.8 Hz), 15.98 (d, *J*_C-P_ = 5.6 Hz), 27.77 (d, *J*_C-P_ = 140.7 Hz), 52.28 (d, *J*_C-P_ = 7.2 Hz), 61.88 (d, *J*_C-P_ = 1.4 Hz), 62.00 (d, *J*_C-P_ = 1.5 Hz), 68.28, 116.65 (d, *J*_C-F_ = 22.3 Hz), 117.40 (d, *J*_C-F_ = 9.6 Hz), 120.89 (d, *J*_C-F_ = 2.6 Hz), 123.92 (d, J_C-F_ = 3.9 Hz), 125.34, 127.79, 128.46, 130.18, 132.00, 134.85 (d, J_C-F_ = 9.1 Hz), 147.46, 159.86, 162.62 (d, J_C-F_ = 3.5 Hz), 163.31; ^31^P-NMR (121.5MHz, CDCl_3_) δ:25.26; HR-MS: for C_22_H_25_FN_3_O_5_P [M+H]^+^ calculated *m/z* 462.15, found *m/z* 462.1588621.

*1-(Diethoxyphosphoryl)-3-(4-phenyl-1H-1,2,3-triazol-1-yl)propan-2-yl 2-chloronicotinate* (**5-A6**). Yield: 75%. White solid. ^1^H-NMR (CDCl_3_) δ 1.30 (t, *J =* 6.3 Hz, 3H), 1.34 (t, *J =* 6.3 Hz, 3H), 2.25–2.34 (m, 2H), 4.08–4.21 (m, 4H), 4.86–5.00 (m, 2H), 5.76–5.83 (m, 1H), 7.30–7.35 (m, 2H), 7.38–7.43 (m, 2H), 7.79–7.82 (m, 2H), 8.04 (S, 1H), 8.24–8.27 (m, 1H), 8.48–8.50 (m, 1H); ^13^C-NMR (CDCl_3_) δ 16.06 (d, *J*_C-P_ = 3.2 Hz), 16.14 (d, *J*_C-P_ = 3.2 Hz), 27.95 (d, *J*_C-P_ = 141.5 Hz), 52.21 (d, *J*_C-P_ = 8.0 Hz), 62.08 (d, *J*_C-P_ = 3.1 Hz), 62.17 (d, *J*_C-P_ = 2.9 Hz), 68.83, 120.89, 122.13, 125.43, 125.76, 128.01, 128.59, 130.01, 140.49, 147.65, 149.52, 152.00, 162.85; ^31^P-NMR (CDCl_3_) δ 24.87; HR-MS: for C_21_H_24_ClN_4_O_5_P [M+H]^+^ calculated *m/z* 479.12, found *m/z* 479.1245606.

*1-(Diethoxyphosphoryl)-3-(4-phenyl-1H-1,2,3-triazol-1-yl)propan-2-yl 4-methoxybenzoate* (**5-A7**). Yield: 65%. White solid. ^1^H-NMR (CDCl_3_) δ 1.25 (t, *J =* 7.1 Hz, 3H), 1.31 (t, *J =* 7.1 Hz, 3H), 2.23–2.32 (m, 2H), 3.81 (s, 3H), 4.05–4.18 (m, 4H), 4.89–4.91 (m, 2H), 5.67–5.75 (m, 1H), 6.87–6.90 (m, 2H), 7.27–7.41 (m, 3H), 7.78–7.80 (m, 2H), 7.95–7.98 (m, 3H); ^13^C-NMR (CDCl_3_) δ:16.00 (d, *J*_C-P_ = 5.9 Hz), 16.08 (d, *J*_C-P_ = 5.6 Hz), 27.95 (d, *J*_C-P_ = 140.6 Hz), 52.36 (d, *J*_C-P_ = 7.2 Hz), 55.16, 61.88 (d, *J*_C-P_ = 1.6 Hz), 61.98 (d, *J*_C-P_ = 1.7 Hz), 67.51, 113.55, 120.80, 121.22, 125.41, 127.86, 128.52, 130.21, 131.59, 147.48, 163.58, 164.70; ^31^P-NMR (CDCl_3_) δ 25.72; HR-MS: for C_23_H_28_N_3_O_6_P [M+H]^+^ calculated *m/z* 474.17, found *m/z* 474.1788486.

*1-(Diethoxyphosphoryl)-3-(4-phenyl-1H-1,2,3-triazol-1-yl)propan-2-yl 3,5-dinitrobenzoate* (**5-A8**). Yield: 77%. White solid. ^1^H-NMR (CDCl_3_) δ 1.32 (t, *J =* 6.9 Hz, 3H), 1.34 (t, *J =* 6.9Hz, 3H), 2.36 (d, *J =* 6.7 Hz, 1H), 2.42 (d, *J =* 6.7 Hz, 1H), 4.11–4.21 (m, 4H), 4.92–5.05 (m, 2H), 5.84–5.91 (m, 1H), 7.22–7.35 (m, 3H), 7.66–7.69 (m, 2H), 8.11 (S, 1H), 8.99–9.04 (m, 3H); ^13^C-NMR (CDCl_3_) δ:16.06 (d, *J*_C-P_ = 5.9 Hz), 27.95 (d, *J*_C-P_ = 141.8 Hz), 52.12 (d, *J*_C-P_ = 8.9 Hz), 62.12 (d, *J*_C-P_ = 6.6 Hz), 62.20 (d, *J*_C-P_ = 6.5 Hz), 69.72, 120.88, 122.30, 125.14, 127.98, 128.48, 129.12, 129.73, 132.57, 147.43, 148.16, 161.22; ^31^P-NMR (CDCl_3_) δ 24.82; HR-MS: for C_22_H_24_N_5_O_9_P [M+H]^+^ *m/z* calculated 534.13, found *m/z* 534.1384403.

*Ethyl 1-(2-(benzoyloxy)-3-(diethoxyphosphoryl)propyl)-1H-1,2,3-triazole-4-carboxylate* (**5-B1**). Yield: 78%. White solid. ^1^H-NMR (CDCl_3_) δ 1.21–1.41 (m, 9H), 2.23–2.29 (m, 2H), 4.06–4.20 (m, 4H), 4.40 (q, *J =* 7.1 Hz, 2H), 4.97 (d, *J =* 4.9 Hz, 2H), 5.67–5.75 (m, 1H), 7.44 (t, *J =* 7.6 Hz, 2H), 7.56–7.61 (m, 1H), 7.98–8.01 (m, 2H), 8.29 (s, 1H); ^13^C-NMR (CDCl_3_) δ:14.08, 16.09 (d, *J*_C-P_ = 6.3 Hz), 16.17 (d, *J*_C-P_ = 6.3 Hz), 28.01 (d, *J*_C-P_ = 140.5 Hz), 52.60 (d, *J*_C-P_ = 6.3 Hz), 61.07, 62.05 (d, *J*_C-P_ = 10.2 Hz), 62.16 (d, *J*_C-P_ = 6.5 Hz), 67.72, 128.40, 128.61, 128.84, 129.60, 133.48, 140.20, 160.37, 165.04; ^31^P-NMR (CDCl_3_) δ 25.19; HR-MS: for C_19_H_26_N_3_O_7_P [M+H]^+^ calculated *m/z* 440.15, found *m/z* 440.1581131.

*Ethyl 1-(3-(diethoxyphosphoryl)-2-((4-nitrobenzoyl)oxy)propyl)-1H-1,2,3-triazole-4-carboxylate* (**5-B2**). Yield: 73%. yellow solid. ^1^H-NMR (CDCl_3_) δ 1.28–1.44 (m, 9H), 2.23–2.34 (m, 2H), 4.10–4.24 (m, 4H), 4.43 (q, *J =* 7.1 Hz, 2H), 5.00–5.03 (m, 2H), 5.73–5.81 (m, 1H), 8.18–8.22 (m, 2H), 8.29–8.34 (m, 3H); ^13^C-NMR (CDCl_3_) δ 13.99, 16.00 (d, *J*_C-P_ = 6.3 Hz), 16.08 (d, *J*_C-P_ = 6.3 Hz), 27.90 (d, *J*_C-P_ = 140.6 Hz), 52.53 (d, *J*_C-P_ = 6.6 Hz), 60.95, 61.95 (d, *J*_C-P_ = 9.2 Hz), 62.05 (d, *J*_C-P_ = 6.7 Hz), 67.63, 128.30, 128.58, 128.76, 129.49, 133.38, 140.06, 160.28, 164.93; ^31^P-NMR (CDCl_3_) δ 24.63; HR-MS: for C_19_H_25_N_4_O_9_P [M+H]^+^ calculated *m/z* 485.14, found *m/z* 485.1431913.

*Ethyl 1-(3-(diethoxyphosphoryl)-2-(2-(naphthalen-1-yl)acetoxy)propyl)-1H-1,2,3-triazole-4-carboxylate* (**5-B3**). Yield: 68%. White solid. ^1^H-NMR (CDCl_3_) δ 1.31 (t, *J =* 7.1 Hz, 6H), 1.42 (t, *J =* 7.1 Hz, 3H), 2.01 (d, *J =* 6.7 Hz, 1H), 2.08 (d, *J =* 6.7 Hz, 1H), 4.05–4.16 (m, 6H), 4.43 (q, *J =* 7.1 Hz, 2H), 4.66 (d, *J =* 4.8 Hz, 2H), 5.43–5.51 (m, 1H), 7.34 (d, *J =* 6.1 Hz, 1H), 7.40–7.55 (m, 3H), 7.71 (s, 1H), 7.79–7.88 (m, 3H); ^13^C-NMR (CDCl_3_) δ 14.19, 16.22 (d, *J*_C-P_ = 5.9 Hz), 27.95 (d, *J*_C-P_ = 140.6 Hz), 38.81, 52.43 (d, *J*_C-P_ = 6.3 Hz), 61.07, 62.16 (d, *J*_C-P_ = 6.2 Hz), 62.24 (d, *J*_C-P_ = 6.0 Hz), 67.52, 123.31, 125.31, 125.75, 126.43, 127.95, 128.27, 128.31, 128.69, 129.40, 131.69, 133.62, 140.05, 160.28, 170.03; ^31^P-NMR (CDCl_3_) δ 24.39; HR-MS: for C_24_H_30_N_3_O_7_P [M+H]^+^ calculated *m/z* 504.18, found *m/z* 504.1894132.

*Ethyl 1-(2-acetoxy-3-(diethoxyphosphoryl)propyl)-1H-1,2,3-triazole-4-carboxylate* (**5-B4**). Yield: 74%. White solid. ^1^H-NMR (CDCl_3_) δ: 1.27–1.44 (m, 9H), 2.04 (s, 3H), 2.05–2.20 (m, 2H), 4.09–4.21 (m, 4H), 4.42 (q, *J =* 7.1 Hz, 2H), 4.76–4.93 (m, 2H), 5.45–5.54 (m, 1H), 8.33 (s, 1H); ^13^C-NMR (CDCl_3_) δ:13.83, 15.93 (d, *J*_C-P_ = 6.0 Hz), 20.33, 27.71 (d, *J*_C-P_ = 141.1 Hz), 52.33 (d, *J*_C-P_ = 7.7 Hz), 60.77, 61.77 (d, *J*_C-P_ = 3.0 Hz), 61.86 (d, *J*_C-P_ = 2.8 Hz), 66.77, 128.37, 139.79, 160.14, 169.07; ^31^P-NMR (CDCl_3_) δ: 25.13; HR-MS: for C_14_H_24_N_3_O_7_P [M+H]^+^ calculated *m/z* 378.14, found *m/z* 378.1424631.

*Ethyl 1-(3-(diethoxyphosphoryl)-2-((2-fluorobenzoyl)oxy)propyl)-1H-1,2,3-triazole-4-carboxylate* (**5-B5**). Yield: 78%. yellow solid. ^1^H-NMR (CDCl_3_) δ 1.26–1.41 (m, 9H), 2.26 (d, *J =* 6.6 Hz, 1H), 2.33 (d, *J =* 6.5 Hz, 1H), 4.08–4.21 (m, 4H), 4.40 (q, *J =* 7.1 Hz, 2H), 5.00–5.01 (m, 2H), 5.72–5.80 (m, 1H), 7.12–7.25 (m, 2H), 7.54–7.61 (m, 1H), 7.90–7.95 (m, 1H), 8.40 (s, 1H); ^13^C-NMR (CDCl_3_) δ 13.85, 15.85 (d, *J*_C-P_ = 5.8 Hz), 15.93 (d, *J*_C-P_ = 5.9 Hz), 27.67 (d, *J*_C-P_ = 140.5 Hz), 52.41 (d, *J*_C-P_ = 6.7 Hz), 60.78, 61.96 (d, *J*_C-P_ = 6.6 Hz), 67.96, 116.64 (d, *J*_C-F_ = 22.2 Hz), 117.14 (d, *J*_C-F_ = 9.4 Hz), 123.89 (d, *J*_C-F_ = 3.9 Hz), 128.67 (d, *J*_C-F_ = 2.1 Hz), 131.92, 134.93 (d, *J*_C-F_ = 9.3 Hz), 139.88, 160.00 (d, *J*_C-F_ = 27.0 Hz), 162.46 (d, *J*_C-F_ = 3.5 Hz), 163.27; ^31^P-NMR (CDCl_3_) δ:24.83; HR-MS: for C_19_H_25_FN_3_O_7_P [M+H]^+^ calculated *m/z* 458.14, found *m/z* 458.1486913.

*1-(Diethoxyphosphoryl)-3-(4-(ethoxycarbonyl)-1H-1,2,3-triazol-1-yl)propan-2-yl 2-chloronicotinate* (**5-B6**). Yield: 76%. yellow solid. ^1^H-NMR (CDCl_3_) δ 1.29–1.42 (m, 9H), 2.25–2.34 (m, 2H), 4.10–4.23 (m, 4H), 4.41 (q, *J =* 7.1 Hz, 2H), 4.93–5.08 (m, 2H), 5.75–5.82 (m, 1H), 7.36–7.40 (m, 1H), 8.25–8.29 (m, 1H), 8.40 (s, 1H), 8.53–8.55 (m, 1H); ^13^C-NMR (CDCl_3_) δ: 13.90, 15.95 (d, *J*_C-P_ = 3.5 Hz), 16.03 (d, *J*_C-P_ = 3.5 Hz), 27.76 (d, *J*_C-P_ = 141.3 Hz), 52.30 (d, *J*_C-P_ = 7.6 Hz), 60.95, 62.09 (d, *J*_C-P_ = 4.5 Hz), 62.18 (d, *J*_C-P_ = 4.4 Hz), 68.49, 122.08, 125.45, 128.64, 139.97, 140.42, 149.46, 152.01, 160.10, 162.64; ^31^P-NMR (CDCl_3_) δ: 24.53; HR-MS: for C_18_H_24_ClN_4_O_7_P [M+H]^+^ calculated *m/z* 475.11, found *m/z* 475.1143897.

*Ethyl 1-(3-(diethoxyphosphoryl)-2-((4-methoxybenzoyl)oxy)propyl)-1H-1,2,3-triazole-4-carboxylate* (**5-B7**). Yield: 80%. White solid. ^1^H-NMR (CDCl_3_) δ 1.20–1.41 (m, 9H), 2.20–2.29 (m, 2H), 3.86 (s, 3H), 4.07–4.20 (m, 4H), 4.40 (q, *J =* 7.1 Hz, 2H), 4.96 (d, *J =* 4.8 Hz, 2H), 5.66–5.70 (m, 1H), 6.89–6.94 (m, 2H), 7.92–7.96 (m, 2H), 8.29 (s, 1H); ^13^C-NMR (CDCl_3_) δ:13.98, 16.00 (d, *J*_C-P_ = 5.7 Hz), 16.07 (d, *J*_C-P_ = 6.0 Hz), 27.89 (d, *J*_C-P_ = 140.5 Hz), 52.55 (d, *J*_C-P_ = 6.3 Hz), 55.20, 60.97, 62.08 (d, *J*_C-__P_ = 6.5 Hz), 67.24, 113.58, 120.96, 128.52, 131.62, 140.05, 160.30, 163.66, 164.63; ^31^P-NMR (CDCl_3_) δ 25.16; HR-MS: for C_20_H_28_N_3_O_8_P [M+H]^+^ calculated *m/z* 470.16, found *m/z* 470.1686778.

*Ethyl 1-(3-(diethoxyphosphoryl)-2-((3,5-dinitrobenzoyl)oxy)propyl)-1H-1,2,3-triazole-4-carboxylate* (**5-B8**). Yield: 76%. White solid. ^1^H-NMR (CDCl_3_) δ 1.31–1.40 (m, 9H), 2.37 (d, *J =* 6.7 Hz, 1H), 2.44 (d, *J =* 6.7 Hz, 1H), 4.13–4.23 (m, 4H), 4.37 (q, *J =* 7.1 Hz, 2H), 5.01–5.14 (m, 2H), 5.84–5.92 (m, 1H), 8.45 (s, 1H), 9.09 (d, *J =* 2.1 Hz, 2H), 9.20–9.22 (m, 1H); ^13^C-NMR (CDCl_3_) δ 13.87, 16.05 (d, *J*_C-P_ = 6.0 Hz), 27.93 (d, *J*_C-P_ = 141.6 Hz), 52.35 (d, *J*_C-P_ = 8.8 Hz), 60.99, 62.18 (d, *J*_C-P_ = 7.2 Hz), 62.28 (d, *J*_C-P_ = 6.9 Hz), 69.54, 122.50, 128.64, 129.29, 132.55, 139.97, 148.35, 160.07, 161.20; ^31^P-NMR (CDCl_3_) δ 24.38; HR-MS: for C_19_H_24_N_5_O_11_P [M+H]^+^ calculated *m/z* 530.12, found *m/z* 530.1282694.

*1-(4-(tert-Butyl)-1H-1,2,3-triazol-1-yl)-3-(diethoxyphosphoryl)propan-2-yl benzoate* (**5-C1**). Yield: 68%. White solid. ^1^H-NMR (CDCl_3_) δ: 1.23–1.35 (m, 15H), 2.20–2.33 (m, 2H), 4.06–4.19 (m, 4H), 4.76–4.87 (m, 2H), 5.65–5.73 (m, 1H), 7.36–7.47 (m, 3H), 7.56–7.61 (m, 1H), 7.99–8.03 (m, 2H); ^13^C-NMR (CDCl_3_) δ: 16.02 (d, *J*_C-P_ = 6.3 Hz), 16.10 (d, *J*_C-P_ = 6.3 Hz), 28.01 (d, *J*_C-P_ = 140.7 Hz), 30.03, 30.41, 52.04 (d, *J*_C-P_ = 7.6 Hz), 61.87 (d, *J*_C-P_ = 1.4 Hz), 61.95 (d, *J*_C-P_ = 1.4 Hz), 67.95, 119.79, 128.25, 129.03, 129.45, 133.27, 157.44, 164.95; ^31^P-NMR (CDCl_3_) δ: 25.58; HR-MS: for C_20_H_30_N_3_O_5_P [M+H]^+^ calculated *m/z* 424.19, found *m/z* 424.199584.

*1-(4-(tert-Butyl)-1H-1,2,3-triazol-1-yl)-3-(diethoxyphosphoryl)propan-2-yl4-nitrobenzoate* (**5-C2**). Yield: 76%. White solid. ^1^H-NMR (CDCl_3_) δ: 1.26–1.38 (m, 15H), 2.27 (d, *J =* 6.6 Hz, 1H), 2.33 (d, *J =* 6.6 Hz, 1H), 4.08–4.21 (m, 4H), 4.83–4.86 (m, 2H), 5.69–5.76 (m, 1H), 7.42 (s, 1H), 8.17–8.21 (m, 2H), 8.27–8.31 (m, 2H); ^13^C-NMR (CDCl_3_) δ: 16.18 (d, *J*_C-P_ = 4.0 Hz), 16.26 (d, *J*_C-P_ = 3.6 Hz), 28.16 (d, *J*_C-P_ = 141.4 Hz), 30.15, 30.56, 52.05 (d, *J*_C-P_ = 7.6 Hz), 62.21 (d, *J*_C-P_ = 6.5 Hz), 68.96, 119.81, 123.48, 130.78, 134.58, 150.65, 157.75, 163.36; ^31^P-NMR (CDCl_3_) δ: 24.99; HR-MS: for C_20_H_29_N_4_O_7_P [M+H]^+^ calculated *m/z* 469.18, found *m/z* 469.1846622.

*1-(4-(tert-Butyl)-1H-1,2,3-triazol-1-yl)-3-(diethoxyphosphoryl)propan-2-yl 2-(naphthalen-1-yl)acetate* (**5-C3**). Yield: 61%. White solid. ^1^H-NMR (CDCl_3_) δ: 1.21–1.33 (m, 15H), 1.91-2.13 (m, 2H), 4.03–4.15 (m, 6H), 4.55–4.56 (m, 2H), 5.42–5.51 (m, 1H), 6.89 (s, 1H), 7.36–7.56 (m, 4H), 7.79–7.87 (m, 2H), 7.94–7.97 (m, 1H); ^13^C-NMR (CDCl_3_) δ: 16.31 (d, *J*_C-P_ = 6.3 Hz), 27.98 (d, *J*_C-P_ = 141.2 Hz), 30.23, 30.53, 38.99, 52.26 (d, *J*_C-P_ = 8.0 Hz), 62.09 (d, *J*_C-P_ = 3.8 Hz), 62.18 (d, *J*_C-P_ = 3.9 Hz), 68.02, 119.39, 123.54, 125.40, 125.87, 126.53, 128.03, 128.28, 128.75, 129.73, 131.90, 133.74, 157.71, 170.22; ^31^P-NMR (CDCl_3_) δ: 25.39; HR-MS: for C_25_H_34_N_3_O_5_P [M+H]^+^ calculated *m/z* 488.22, found *m/z* 488.2308841.

*1-(4-(tert-Butyl)-1H-1,2,3-triazol-1-yl)-3-(diethoxyphosphoryl)propan-2-yl acetate* (**5-C4**). Yield: 71%. White solid. ^1^H-NMR (CDCl_3_) δ 1.26–1.37 (m, 15H), 2.03–2.21 (m, 5H), 4.08–4.19 (m, 4H), 4.58–4.76 (m, 2H), 5.41–5.47 (m, 1H), 7.37 (s, 1H); ^13^C-NMR (CDCl_3_) δ 16.16 (d, *J*_C-P_ = 6.1 Hz), 20.60, 28.02 (d, *J*_C-P_ = 141.2 Hz), 30.11, 30.47, 52.02 (d, *J*_C-P_ = 7.9 Hz), 61.92 (d, *J*_C-P_ = 6.3 Hz), 67.34, 119.48, 157.53, 169.36; ^31^P-NMR (CDCl_3_) δ 26.22; HR-MS: for C_15_H_28_N_3_O_5_P [M+H]^+^ calculated *m/z* 362.18, found *m/z* 362.183934.

*1-(4-(tert-Butyl)-1H-1,2,3-triazol-1-yl)-3-(diethoxyphosphoryl)propan-2-yl 2-fluorobenzoate* (**5-C5**). Yield: 66%. yellow solid. ^1^H-NMR (CDCl_3_) δ: 1.25–1.38 (m, 15H), 2.18–2.33 (m, 2H), 4.08–4.20 (m, 4H), 4.82–4.84 (m, 2H), 5.70–5.74 (m, 1H), 7.11–7.25 (m, 2H), 7.49 (s, 1H), 7.54–7.57 (m, 1H), 7.90–7.95 (m, 1H); ^13^C-NMR (CDCl_3_) δ: 15.99 (d, *J*_C-P_ = 5.9 Hz), 16.07 (d, *J*_C-P_ = 5.9 Hz), 27.92 (d, *J*_C-P_ = 141.0 Hz), 30.01, 30.41, 52.11 (d, *J*_C-P_ = 7.6 Hz), 61.99 (d, *J*_C-P_ = 1.2 Hz), 62.07 (d, *J*_C-P_ = 1.4 Hz), 68.42, 116.72 (d, *J*_C-F_ = 22.3 Hz), 117.55 (d, *J*_C-F_ = 9.5 Hz), 119.91 (d, *J*_C-F_ = 2.3 Hz), 123.96 (d, *J*_C-F_ = 3.9 Hz), 132.07, 134.90 (d, *J*_C-F_ = 9.2 Hz), 157.45, 162.67 (d, *J*_C-F_ = 3.5 Hz), 163.40; ^31^P-NMR (CDCl_3_) δ:24.90; HR-MS: for C_20_H_29_FN_3_O_5_P [M+H]^+^ calculated *m/z* 442.18, found *m/z* 442.1901622.

*1-(4-(tert-Butyl)-1H-1,2,3-triazol-1-yl)-3-(diethoxyphosphoryl)propan-2-yl 4-methoxybenzoate* (**5-C6**). Yield: 43%. yellow solid. ^1^H-NMR (CDCl_3_) δ 1.21–1.37 (m, 15H), 2.19–2.29 (m, 2H), 3.87 (s, 3H), 4.05–4.18 (m, 4H), 4.78–4.80 (m, 2H), 5.58–5.67 (m, 1H), 6.89–6.94 (m, 2H), 7.35 (s, 1H), 7.93–7.98 (m, 2H); ^13^C-NMR (CDCl_3_) δ: 16.23 (d, *J*_C-P_ = 6.0 Hz), 16.30 (d, *J*_C-P_ = 5.9 Hz), 28.27 (d, *J*_C-P_ = 140.7 Hz), 30.24, 30.62, 52.28 (d, *J*_C-P_ = 7.4 Hz), 55.41, 62.05 (d, *J*_C-P_ = 1.9 Hz), 62.13 (d, *J*_C-P_ = 2.0 Hz), 67.82, 113.73, 119.89, 121.55, 131.78, 157.68, 163.79, 164.91; ^31^P-NMR (CDCl_3_) δ 25.68; HR-MS: for C_21_H_32_N_3_O_6_P [M+H]^+^ calculated *m/z* 454.20, found *m/z* 454.2101487.

*1-(4-(tert-Butyl)-1H-1,2,3-triazol-1-yl)-3-(diethoxyphosphoryl)propan-2-yl 3,5-dinitrobenzoate* (**5-C7**). Yield: 63%. White solid. ^1^H-NMR (CDCl_3_) δ: 1.24–1.37 (m, 15H), 2.27–2.35 (m, 2H), 4.10–4.21 (m, 4H), 4.77–4.89 (m, 2H), 5.76–5.84 (m, 1H), 7.42 (s, 1H), 9.09–9.10 (m, 2H), 9.23–9.24 (m, 1H); ^13^C-NMR (CDCl_3_) δ: 16.33 (d, *J*_C-P_ = 5.8 Hz), 28.39 (d, *J*_C-P_ = 141.9 Hz), 30.19, 30.66, 52.06 (d, *J*_C-P_ = 8.6 Hz), 62.34 (d, *J*_C-P_ = 6.8 Hz), 62.43 (d, *J*_C-P_ = 6.8 Hz), 69.96, 119.69, 122.65, 129.43, 133.06, 148.65, 158.11, 161.37 ; ^31^P-NMR (CDCl_3_) δ:24.65; HR-MS: for C_20_H_28_N_5_O_9_P [M+H]^+^ calculated *m/z* 514.16, found *m/z* 514.1697404.

*1-(4-(Chloromethyl)-1H-1,2,3-triazol-1-yl)-3-(diethoxyphosphoryl)propan-2-yl benzoate* (**5-D1**). Yield: 76%. White solid. ^1^H-NMR (CDCl_3_) δ 1.20–1.35 (m, 6H), 2.22 (d, *J =* 6.5 Hz, 1H), 2.28 (d, *J =* 6.5 Hz, 1H), 4.06–4.19 (m, 4H), 4.69 (s, 2H), 4.88–4.90 (m, 2H), 5.65–5.73 (m, 1H), 7.45 (t, *J =* 7.6 Hz, 2H), 7.56–7.61 (m, 1H), 7.78 (s, 1H), 7.98–8.01 (m, 2H); ^13^C-NMR (CDCl_3_) δ: 16.12 (d, *J*_C-P_ = 6.3 Hz), 16.20 (d, *J*_C-P_ = 6.3 Hz), 28.04 (d, *J*_C-P_ = 140.7 Hz), 35.90, 52.45 (d, *J*_C-P_ = 6.8 Hz), 62.13 (d, *J*_C-P_ = 6.6 Hz), 67.86, 123.99, 128.41, 128.95, 129.61, 133.46, 144.68, 165.09; ^31^P-NMR (CDCl_3_) δ: 25.14; HR-MS: for C_17_H_23_ClN_3_O_5_P [M+H]^+^ calculated *m/z* 416.11, found *m/z* 416.1136615.

*1-(4-(Chloromethyl)-1H-1,2,3-triazol-1-yl)-3-(diethoxyphosphoryl)propan-2-yl 4-nitrobenzoate* (**5-D2**). Yield: 78%. White solid. ^1^H-NMR (CDCl_3_) δ: 1.29 (t, *J =* 7.1 Hz, 3H), 1.34 (t, *J =* 7.1 Hz, 3H), 2.26 (d, *J =* 6.9 Hz, 1H), 2.32 (d, *J =* 7.1 Hz, 1H), 4.08–4.21 (m, 4H), 4.70 (s, 2H), 4.91–4.94 (m, 2H), 5.72–5.76 (m, 1H), 7.83 (s, 1H), 8.17 (d, *J =* 2.0 Hz, 1H), 8.20 (d, *J =* 1.9 Hz, 1H), 8.27 (d, *J =* 2.0 Hz, 1H), 8.29 (d, *J =* 1.7 Hz, 1H); ^13^C-NMR (CDCl_3_) δ: 16.15 (d, *J*_C-P_ = 3.8 Hz), 16.23 (d, *J*_C-P_ = 3.7 Hz), 28.04 (d, *J*_C-P_ = 141.1 Hz), 35.85, 52.28 (d, *J*_C-P_ = 7.0 Hz), 62.20 (d, *J*_C-P_ = 1.7 Hz), 62.28 (d, *J*_C-P_ = 1.5 Hz), 68.77, 123.49, 124.00, 130.79, 134.39, 144.75, 150.64, 163.32; ^31^P-NMR (CDCl_3_) δ:24.70; HR-MS: for C_17_H_22_ClN_4_O_7_P [M+H]^+^ calculated *m/z* 461.09, found *m/z* 461.0987397.

*1-(4-(Chloromethyl)-1H-1,2,3-triazol-1-yl)-3-(diethoxyphosphoryl)propan-2-yl 2-(naphthalen-1-yl)acetate* (**5-D3**). Yield: 80%. White solid. ^1^H-NMR (CDCl_3_) δ: 1.26–1.35 (m, 6H), 1.94–2.04 (m, 2H), 4.06–4.16 (m, 6H), 4.41–4.46 (m, 2H), 4.51–4.53 (m, 2H), 5.41–5.44 (m, 1H), 6.59 (s, 1H), 7.37–7.57 (m, 4H), 7.84–7.96 (m, 3H); ^13^C-NMR (CDCl_3_) δ: 16.28 (d, *J*_C-P_ = 5.9 Hz), 27.90 (d, *J*_C-P_ = 141.0 Hz), 35.67, 39.11, 52.39 (d, *J*_C-P_ = 7.4 Hz), 62.16 (d, *J*_C-P_ = 6.6 Hz), 62.25 (d, *J*_C-P_ = 6.5 Hz), 67.72, 123.50, 125.48, 126.01, 126.63, 128.22, 128.27, 128.75, 129.70, 131.80, 133.67, 144.29, 169.87; ^31^P-NMR (CDCl_3_) δ: 25.14; HR-MS: for C_22_H_27_ClN_3_O_5_P [M+H]^+^ calculated *m/z* 480.14, found *m/z* 480.1449616.

*1-(4-(Chloromethyl)-1H-1,2,3-triazol-1-yl)-3-(diethoxyphosphoryl)propan-2-yl acetate* (**5-D4**). Yield: 54%. White solid. ^1^H-NMR (CDCl_3_) δ 1.31–1.38 (m, 6H), 2.05–2.15 (m, 2H), 2.06 (s, 3H), 4.09–4.20 (m, 4H), 4.65–4.82 (m, 4H), 5.41–5.48 (m, 1H), 7.74 (s, 1H); ^13^C-NMR (CDCl_3_) δ 16.31 (d, *J*_C-P_ = 6.0 Hz), 20.76, 28.14 (d, *J*_C-P_ = 141.0 Hz), 36.00, 52.44 (d, *J*_C-P_ = 6.9 Hz), 62.17 (d, *J*_C-P_ = 3.3 Hz), 62.26 (d, *J*_C-P_ = 3.2 Hz), 67.31, 123.85, 144.81, 169.54; ^31^P-NMR (CDCl_3_) δ 25.19; HR-MS: for C_12_H_21_ClN_3_O_5_P [M+H]^+^ calculated *m/z* 354.09, found *m/z* 354.0980115.

*1-(4-(Chloromethyl)-1H-1,2,3-triazol-1-yl)-3-(diethoxyphosphoryl)propan-2-yl 2-fluorobenzoate* (**5-D5**). Yield: 52%. White solid. ^1^H-NMR (CDCl_3_) δ 1.25–1.38 (m, 6H), 2.23–2.31 (m, 2H), 4.08–4.20 (m, 4H), 4.70 (s, 2H), 4.89–4.91 (m, 2H), 5.70–5.74 (m, 1H), 7.12–7.25 (m, 2H), 7.55–7.58 (m, 1H), 7.85 (s, 1H), 7.89–7.95 (m, 1H); ^13^C-NMR (CDCl_3_) δ 16.05 (d, *J*_C-P_ = 5.9 Hz), 16.13 (d, *J*_C-P_ = 5.9 Hz), 27.93 (d, *J*_C-P_ = 140.8 Hz), 35.86, 52.46 (d, *J*_C-P_ = 6.8 Hz), 62.18 (d, *J*_C-P_ = 6.5 Hz), 68.29, 116.84 (d, *J*_C-F_ = 22.3 Hz), 124.06 (d, *J*_C-F_ = 2.1 Hz), 124.09 (d, *J*_C-F_ = 1.9 Hz), 132.13, 135.05 (d, *J*_C-F_ = 9.1 Hz), 144.58, 160.04, 162.71 (d, *J*_C-F_ = 3.5 Hz), 163.48; ^31^P-NMR (CDCl_3_) δ 25.02; HR-MS: for C_17_H_22_ClFN_3_O_5_P [M+H]^+^ calculated *m/z* 434.10, found *m/z* 434.1042397.

*1-(4-(Chloromethyl)-1H-1,2,3-triazol-1-yl)-3-(diethoxyphosphoryl)propan-2-yl 4-methoxybenzoate* (**5-D6**). Yield: 34%. White solid. ^1^H-NMR (CDCl_3_) δ: 1.24–1.38 (m, 6H), 2.19 (d, *J =* 6.6 Hz, 1H), 2.25 (d, *J =* 6.6 Hz, 1H), 3.87 (s, 3H), 4.06–4.19 (m, 4H), 4.69 (s, 2H), 4.86–4.88 (m, 2H), 5.62–5.66 (m, 1H), 6.90–6.95 (m, 2H), 7.74 (s, 1H), 7.93–7.98 (m, 2H); ^13^C-NMR (CDCl_3_) δ: 16.26 (d, *J*_C-P_ = 6.0 Hz), 16.33 (d, *J*_C-P_ = 5.7 Hz), 28.20 (d, *J*_C-P_ = 140.5 Hz), 36.04, 52.62 (d, *J*_C-P_ = 6.4 Hz), 55.45, 62.24 (d, *J*_C-P_ = 6.6 Hz), 67.63, 113.83, 121.35, 124.03, 131.87, 144.83, 163.90, 164.93; ^31^P-NMR (CDCl_3_) δ:25.39; HR-MS: for C_18_H_25_ClN_3_O_6_P [M+H]^+^ calculated *m/z* 446.12, found *m/z* 446.1242262.

*1-(4-(Chloromethyl)-1H-1,2,3-triazol-1-yl)-3-(diethoxyphosphoryl)propan-2-yl 3,5-dinitrobenzoate* (**5-D7**). Yield: 76%. White solid. ^1^H-NMR (CDCl_3_) δ 1.26–1.38 (m, 6H), 2.32 (d, *J =* 6.8 Hz, 1H), 2.39 (d, *J =* 7.0 Hz, 1H), 4.12–4.23 (m, 4H), 4.68 (s, 2H), 4.88–5.02 (m, 2H), 5.79–5.87 (m, 1H), 7.87 (s, 1H), 9.09–9.10 (m, 2H), 9.21–9.22 (m, 1H); ^13^C-NMR (CDCl_3_) δ 16.18 (d, *J*_C-P_ = 5.9 Hz), 28.13 (d, *J*_C-P_ = 141.8 Hz), 35.76, 52.24 (d, *J*_C-P_ = 8.4 Hz), 62.30 (d, *J*_C-P_ = 6.8 Hz), 62.40 (d, *J*_C-P_ = 6.6 Hz), 69.71, 122.60, 124.00, 129.38, 132.73, 144.74, 148.49, 161.27; ^31^P-NMR (CDCl_3_) δ 24.38; HR-MS: for C_17_H_21_ClN_5_O_9_P [M+H]^+^ calculated *m/z* 506.08, found *m/z* 506.0838179.

### 3.3. In Vivo Herbicidal Activity

For the preliminary herbicidal activity screen the growth inhibition effect on the dicotyledon rape and the monocotyledon wheat was examined with a standard Petri dish test. Briefly, the compounds to be tested were dissolved in DMF and emulsified with Tween^®^ 80, and the solutions were diluted with water to the concentrations of 100 mg/L and 10 mg/L, respectively. The solution (9 mL) was added to a Petri dish and two pieces of filter paper were placed on the dish bottom. Twenty seeds of each of rape and wheat were placed on the filter paper. The covered Petri dish was transferred into an artificial climate incubator, where the condition was controlled-temperature 25 °C, room humidity 80%, light intensity 10 Klux, and photoperiod 12 h/day. The incubation was continued for 5 days. The lengths of all roots and stalks were measured and the percentage inhibition was calculated relative to controls using distilled water.

Further herbicidal activity was examined using a pot experiment with barnyard grass [6]. Briefly, the compounds to be tested (**5-A3**, **5-B3**, **5-B4**, **5-C2**, **5-C3**, **5-D3**) were dissolved in DMF and emulsified with Tween^®^ 80, and the solutions were diluted with water to the concentrations of 4 kg/hm^2^, 1 kg/hm^2^, 250 g/hm^2^ and 62.5 g/hm^2^, respectively. Ten germinated seeds of barnyard grass were 5 mm below the soil in each 8 cm × 8 cm flowerpot. Pre-emergence and post-emergence treatment was applied for each compound at concentration. For pre-emergence treatment, plants were treated with compounds 10 days after seeds were sowed while for post-emergence, plants were treated with compounds 24 h after seeds were sowed. The pottings were transferred into an artificial climate incubator, where the condition was controlled at temperature 25 °C, room humidity 80%, light intensity 10 Klux, and photoperiod 12 h/day. The incubation was stopped two weeks after plants were treated. The fresh weight were measured and the fresh weight reduction ratios were calculated relative to controls using distilled water, compared with 62.5 g/hm^2^ acetochlor.

## 4. Conclusions

In summary, a series of novel 1-(diethoxyphosphoryl)-3-(4-one-1*H*-1,2,3-triazol-1-yl)propan-2-yl carboxylic esters were synthesized and their herbicidal activities were evaluated. The bioassays showed that most of the tested compounds have good herbicidal activities against rape and wheat at 100 mg/L. Among all the novel compounds tested, compounds 5-A3 and 5-B4 displayed better herbicidal activities than IRL1803 ([3-hydroxy-3-(1*H*-[1,2,4]triazol-3-yl)-cyclohexyl]-phosphonic acid, 1000 g/hm^2^) which has the best herbicidal activity ever reported. Both compounds **5-A3** and **5-B4**, which can efficiently inhibit the growth of barnyard grass at the concentration of 250 g/hm^2^, displayed a nearly 4-fold increase in the herbicidal potency. Furthermore, using the preliminary bioassay, compounds whose R_2_ is α-naphthylmethylene (5-A3, 5-B3, 5-C3, 5-D3) exhibited much better inhibitory activity than other target compounds. This observation supports the conclusion of Schweitzer, whereby the naphthyl ring fills the volume of the cavity in the enzyme even more efficiently than the phenyl ring, or the π-π interaction of the naphthyl ring with an aromatic residue of the protein may be stronger than with the phenyl ring. This may help us develop new herbicides based on the new potential target enzyme imidazole glycerol phosphate dehydratase (IGPD).

## Supplementary Materials

Supplementary materials can be accessed at: http://www.mdpi.com/1420-3049/20/01/1088/s1.
