# Supplementary material for: Synthesis and Herbicidal Activity of Novel 1-(Diethoxy-phosphoryl)-3-(4-one-1H-1,2,3-triazol-1-yl)-propan-2-yl Carboxylic Esters
Source: Molecules. 2015 Jan 12;20(1):1088–103. doi: 10.3390/molecules20011088 (PMC6272695; doi:10.3390/molecules20011088)

Supplementary Materials

Figure S1. ^{1}H-NMR spectrum of compound **5**–**A1**.

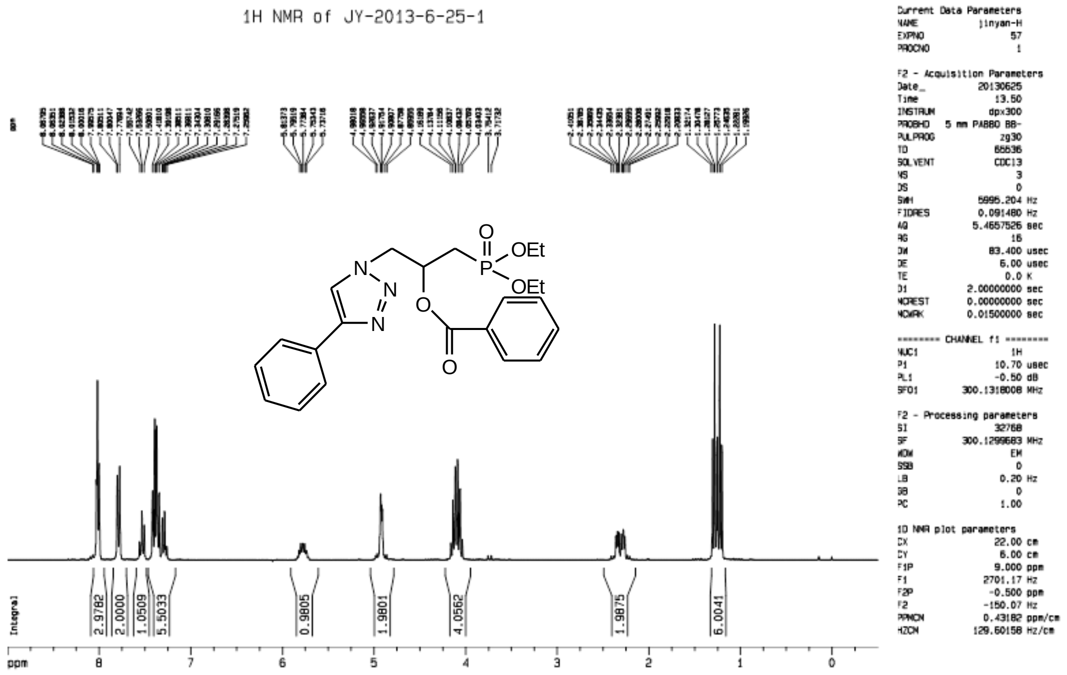

Figure S2. ^{13}C-NMR spectrum of compound **5**–**A1**.

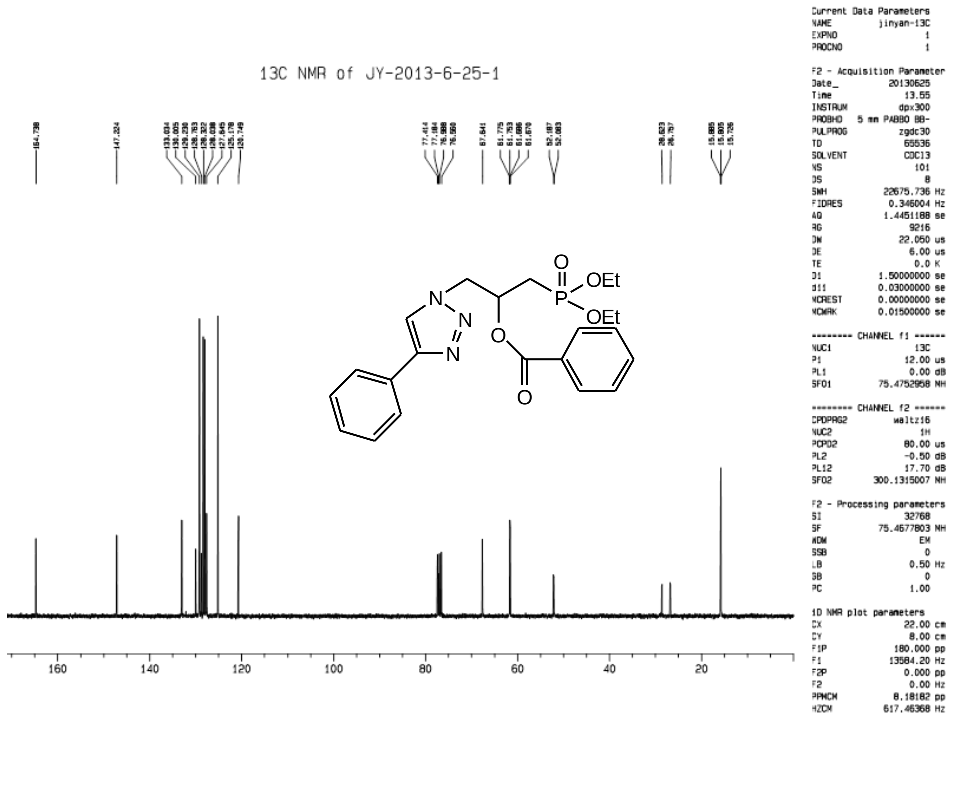

Figure S3. ^{31}P-NMR spectrum of compound **5–A1**.

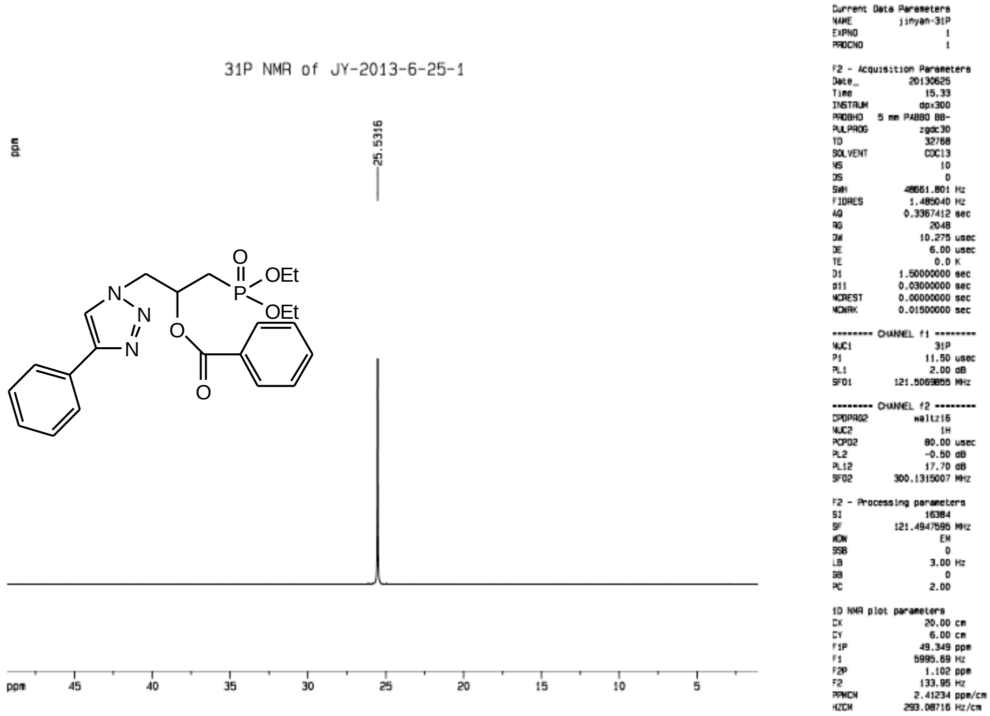

Figure S4. HRMS spectrum of compound **5–A1**.

5-A1[M+H]444.1682839

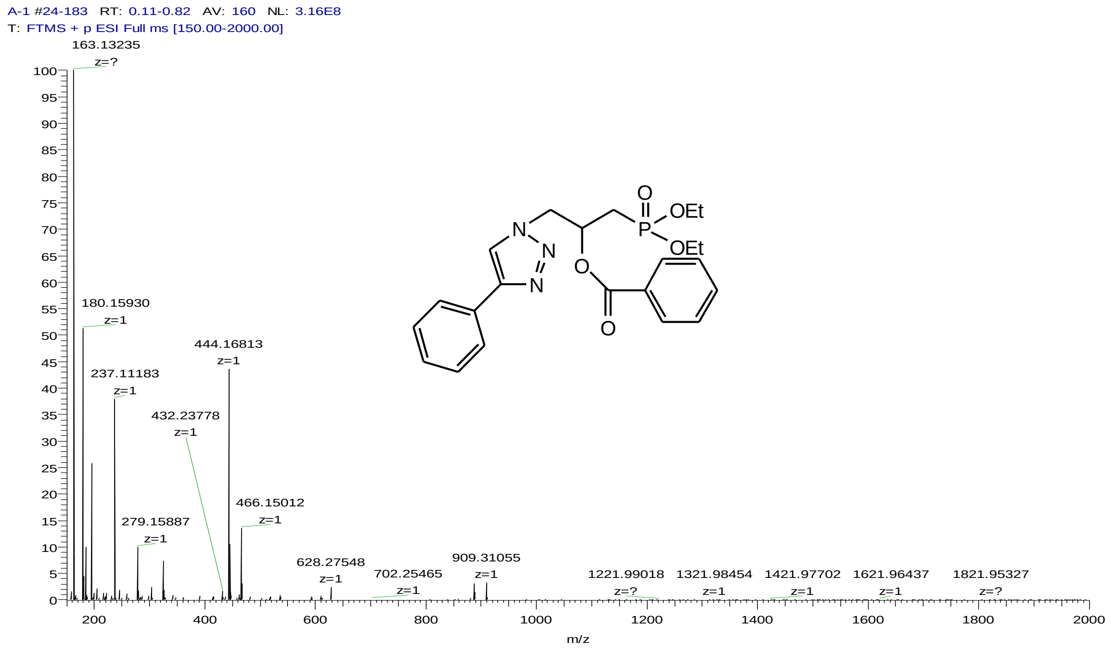

Figure S5. HRMS spectrum of compound **5–A1**.

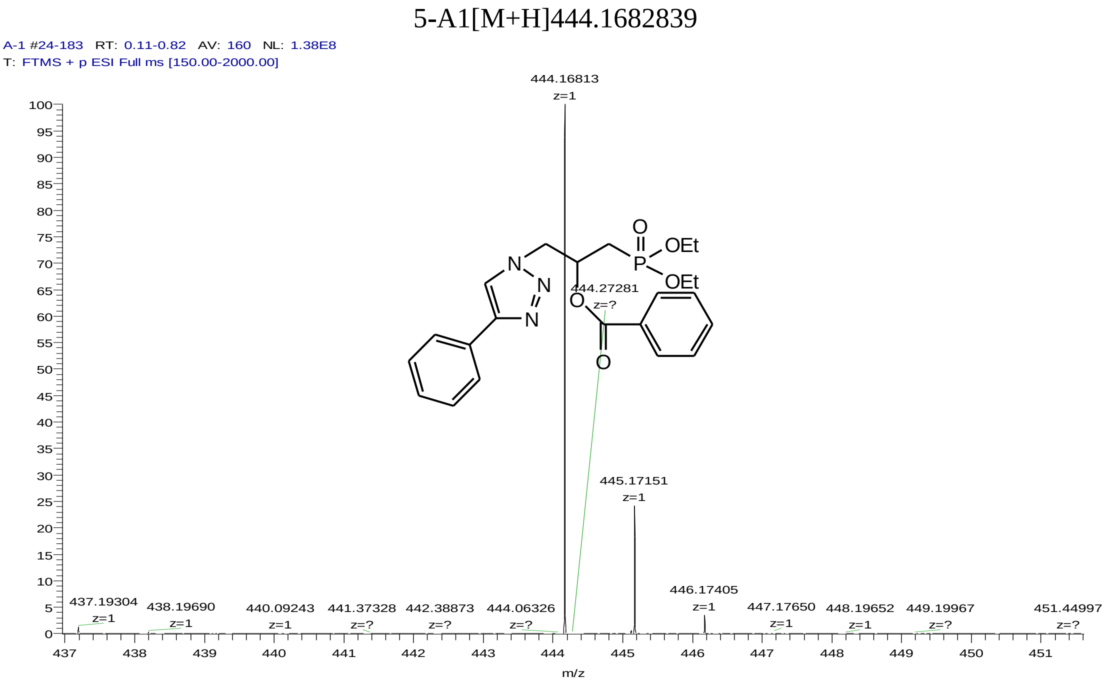

Figure S6. ^{1}H-NMR spectrum of compound **5-A2**.

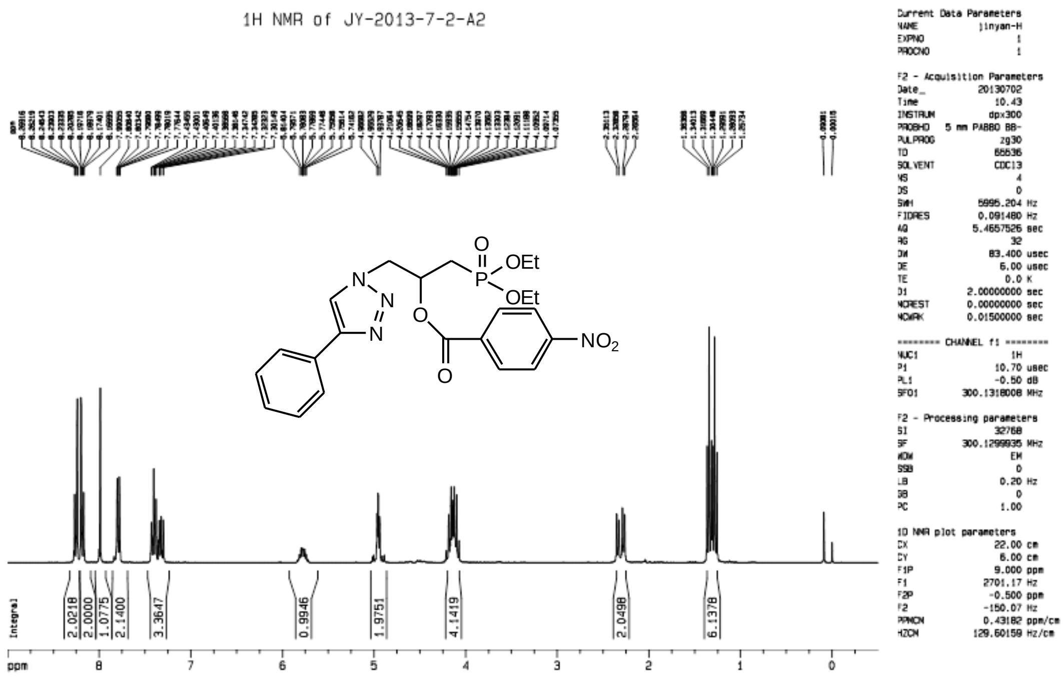

Figure S7. ^{13}C-NMR spectrum of compound **5-A2**.

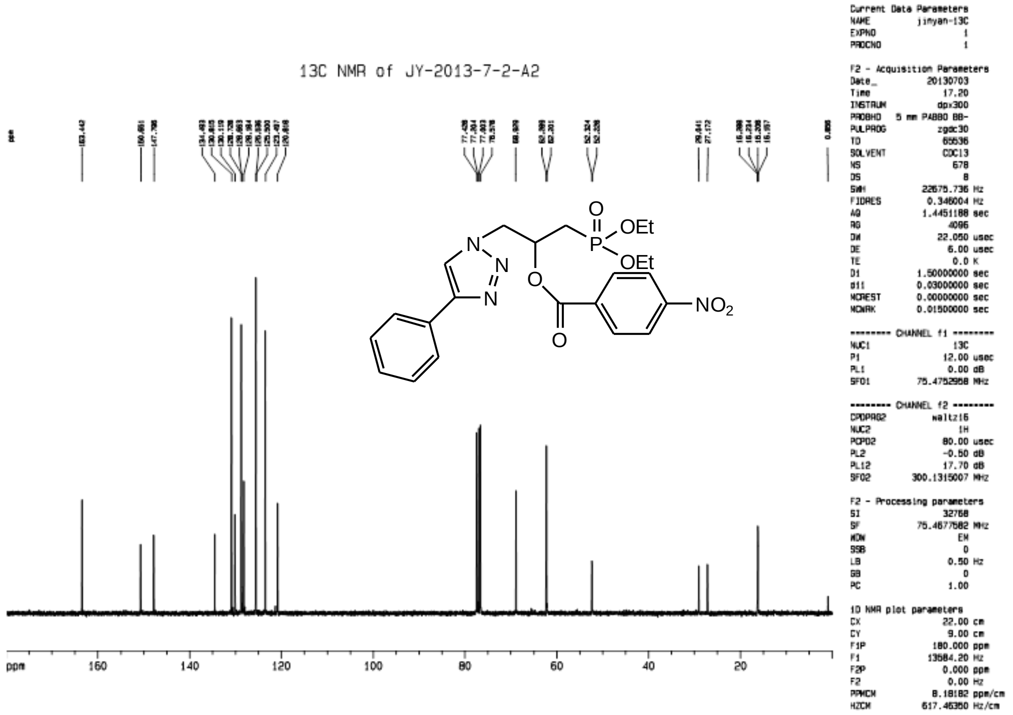

Figure S8. ^{31}P-NMR spectrum of compound **5-A2**.

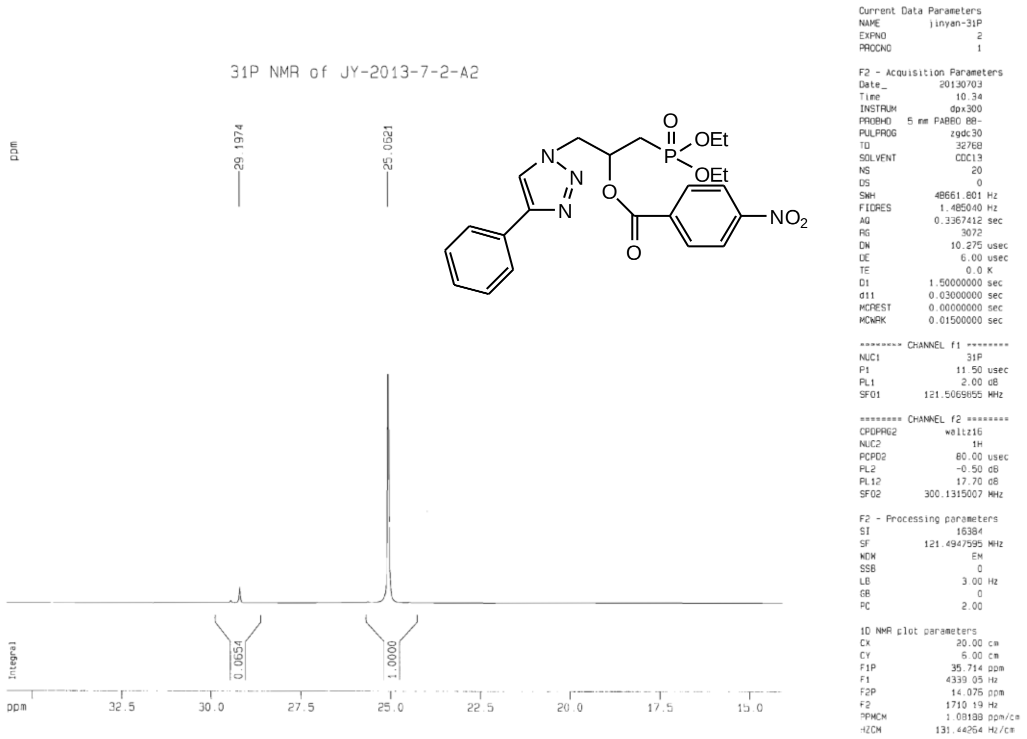

Figure S9. HRMS spectrum of compound **5-A2**.

5-A2 [M+H] 489.1533621

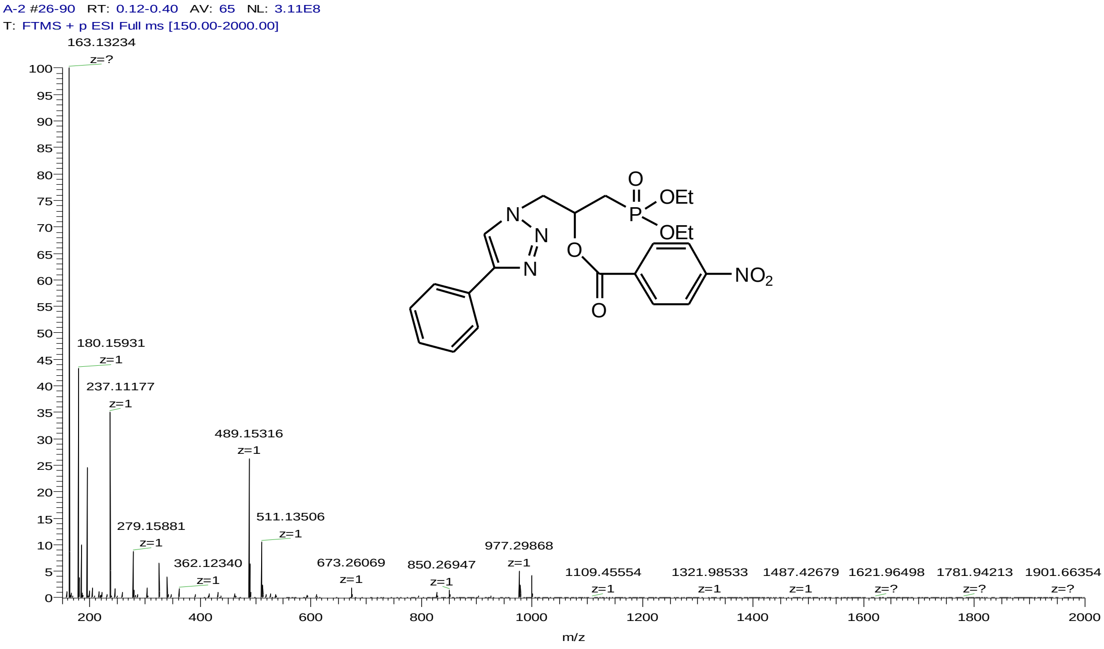

Figure S10. HRMS spectrum of compound **5-A2**.

5-A2 [M+H] 489.1533621

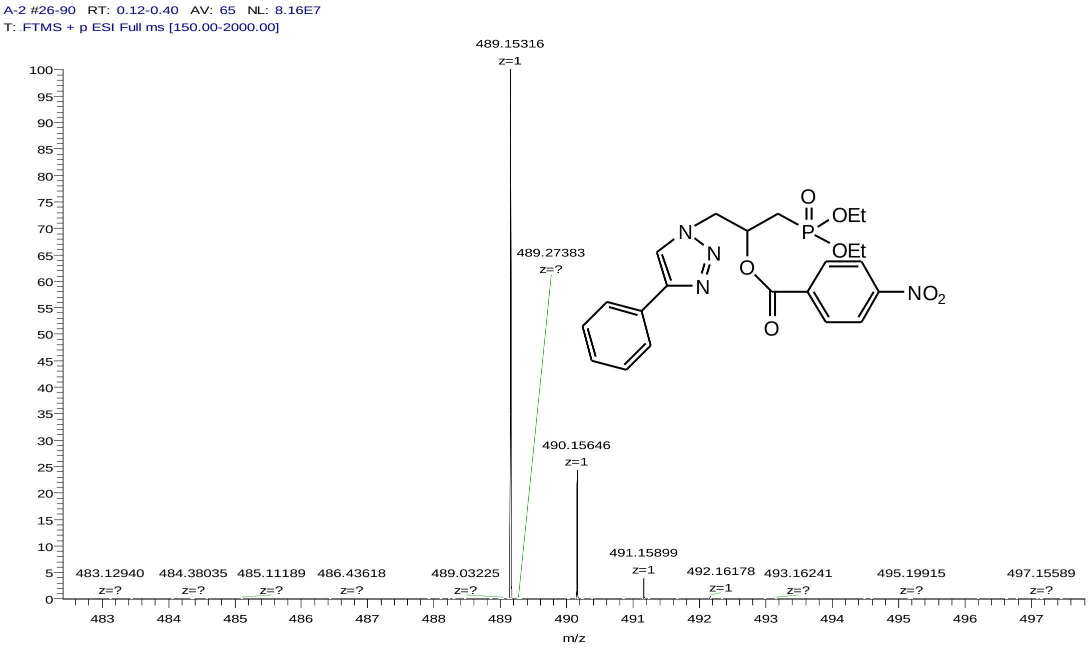

•

Figure S11. ^{1}H-NMR spectrum of compound **5-A3**.

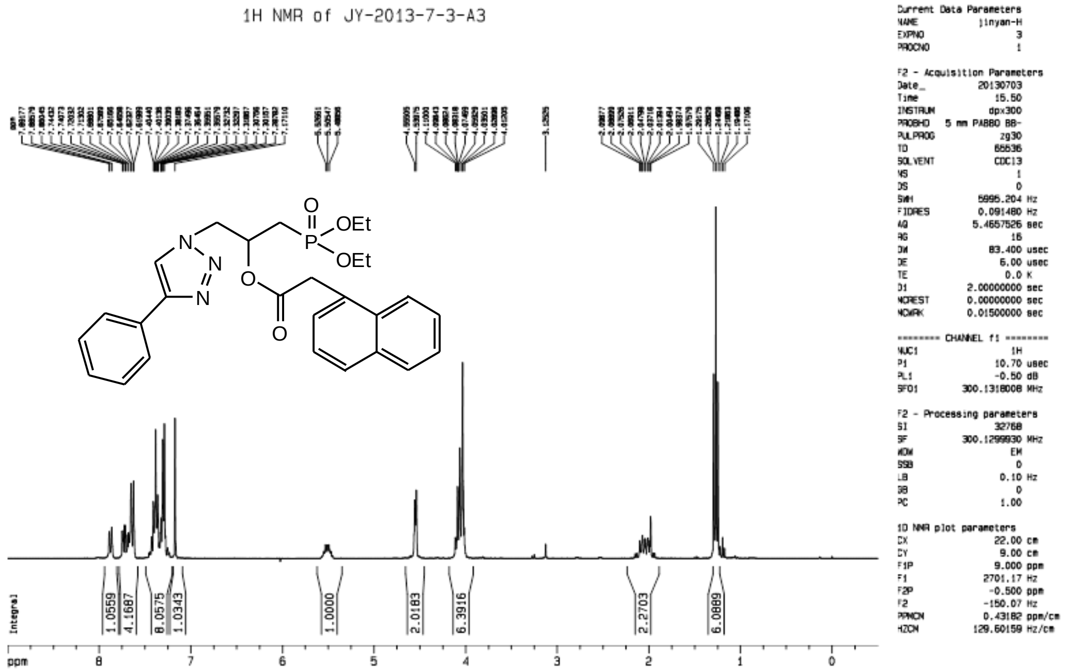

Figure S12. ^{13}C-NMR spectrum of compound **5-A3**.

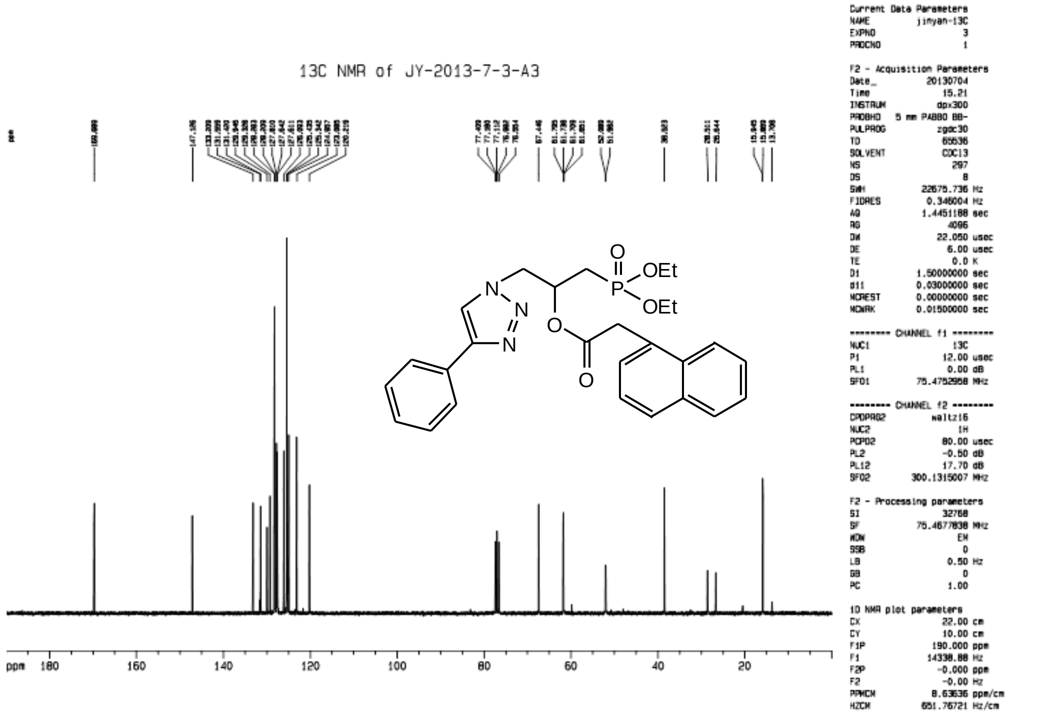

Figure S13. ^{31}P-NMR spectrum of compound **5-A3**.

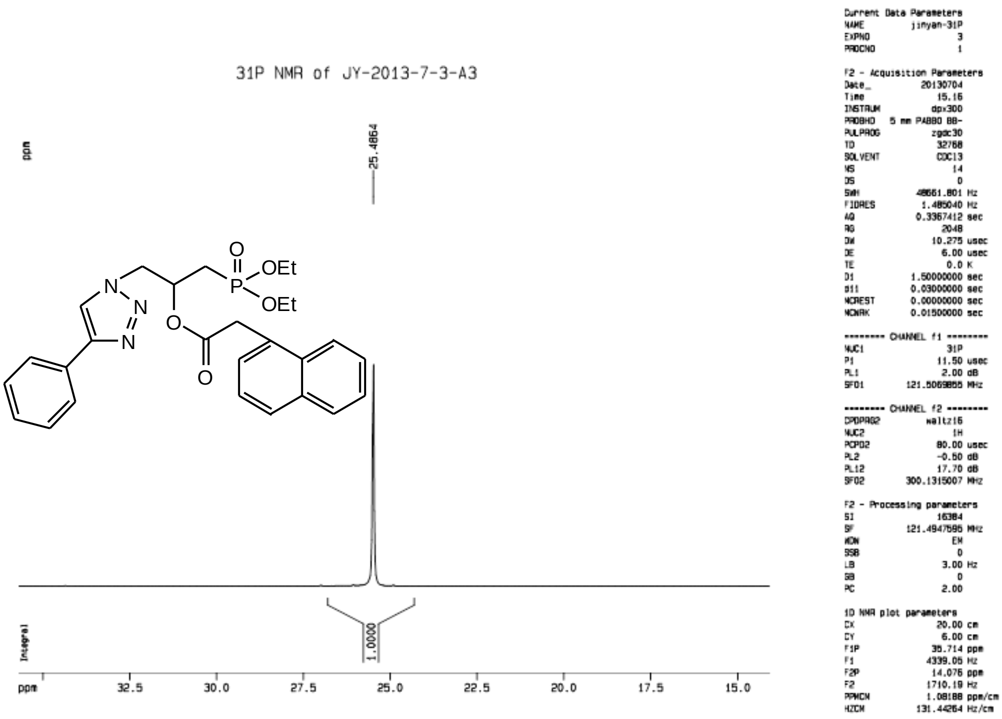

Figure S14. HRMS spectrum of compound **5-A3**.

5-A3 [M+H]508.1995840

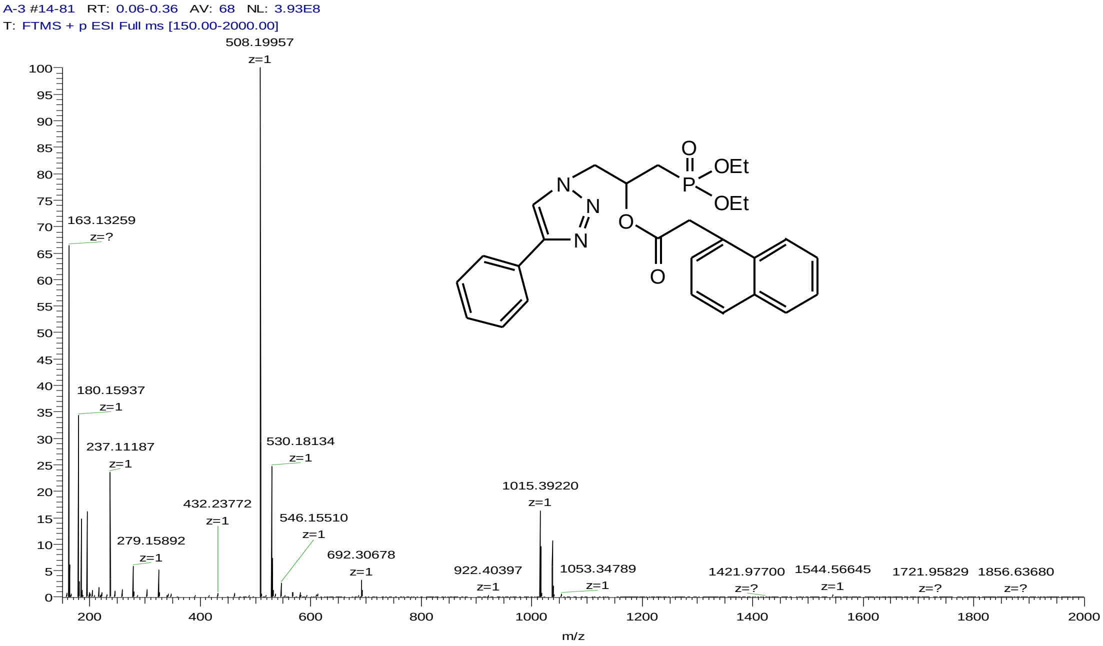

Figure S15. HRMS spectrum of compound **5-A3**.

5-A3 [M+H] 508.1995840

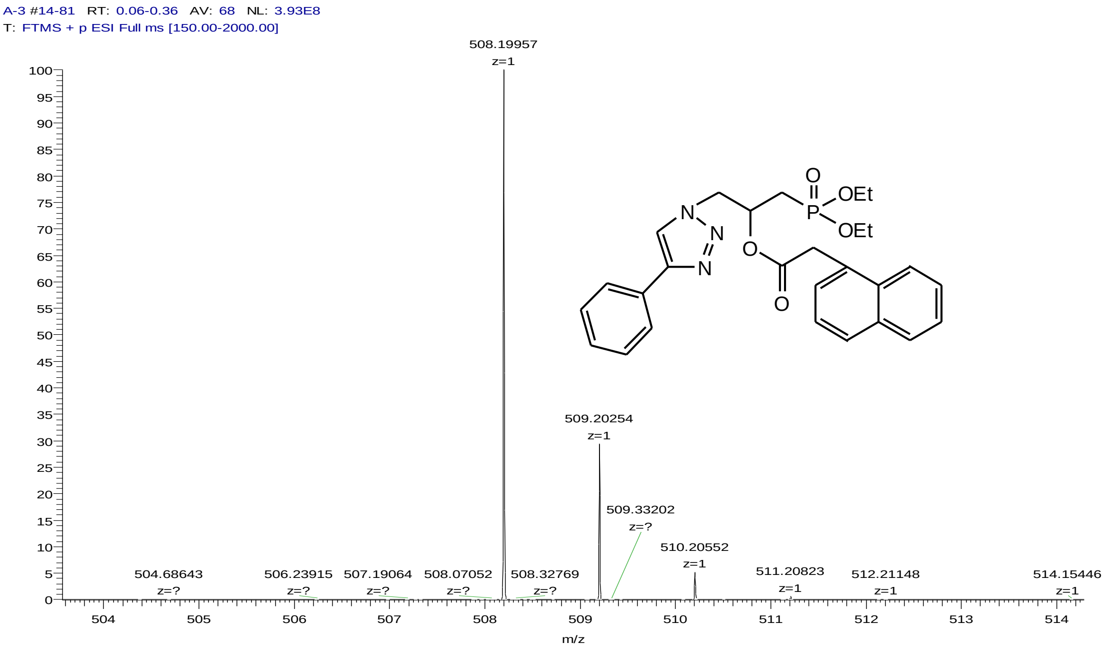

Figure S16. ^{1}H-NMR spectrum of compound **5-A4**.

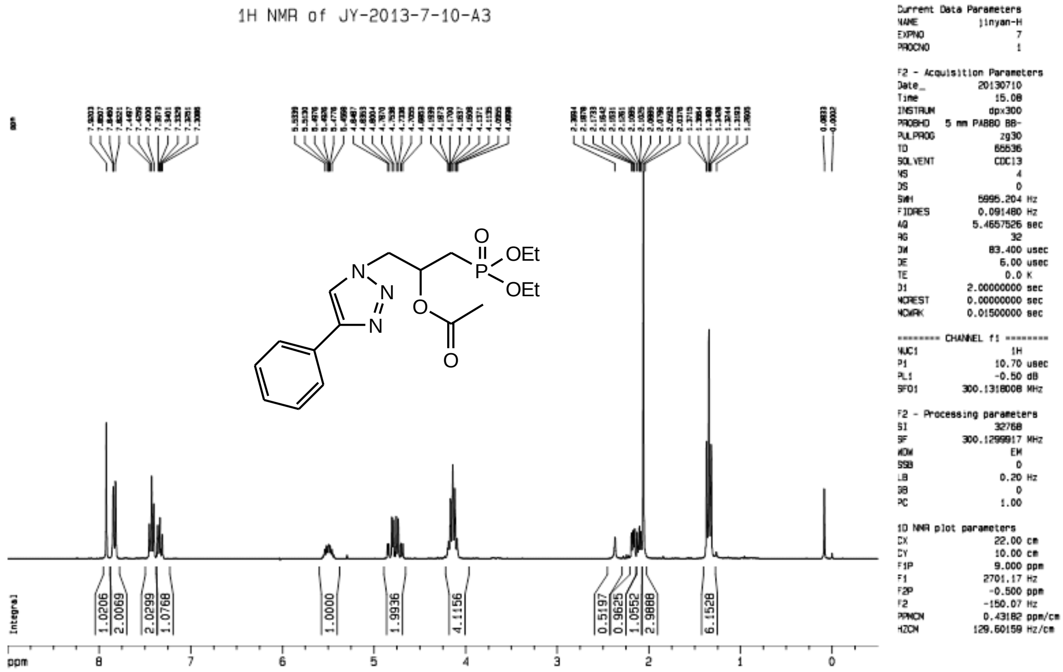

Figure S17. ^{13}C-NMR spectrum of compound **5-A4**.

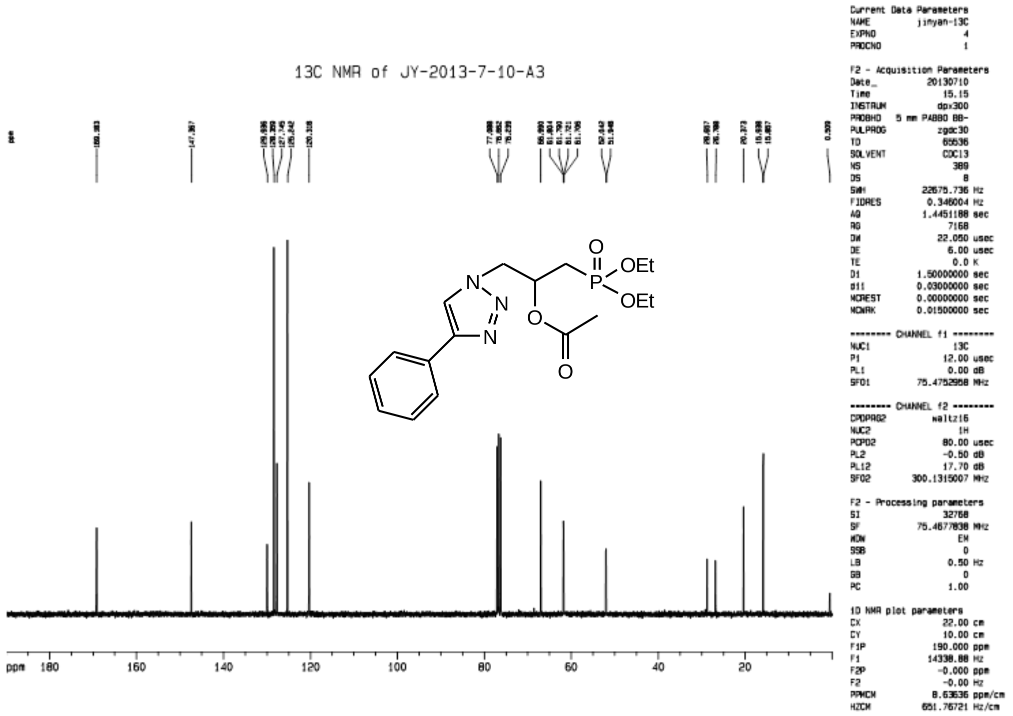

Figure S18. ^{31}P-NMR spectrum of compound **5-A4**.

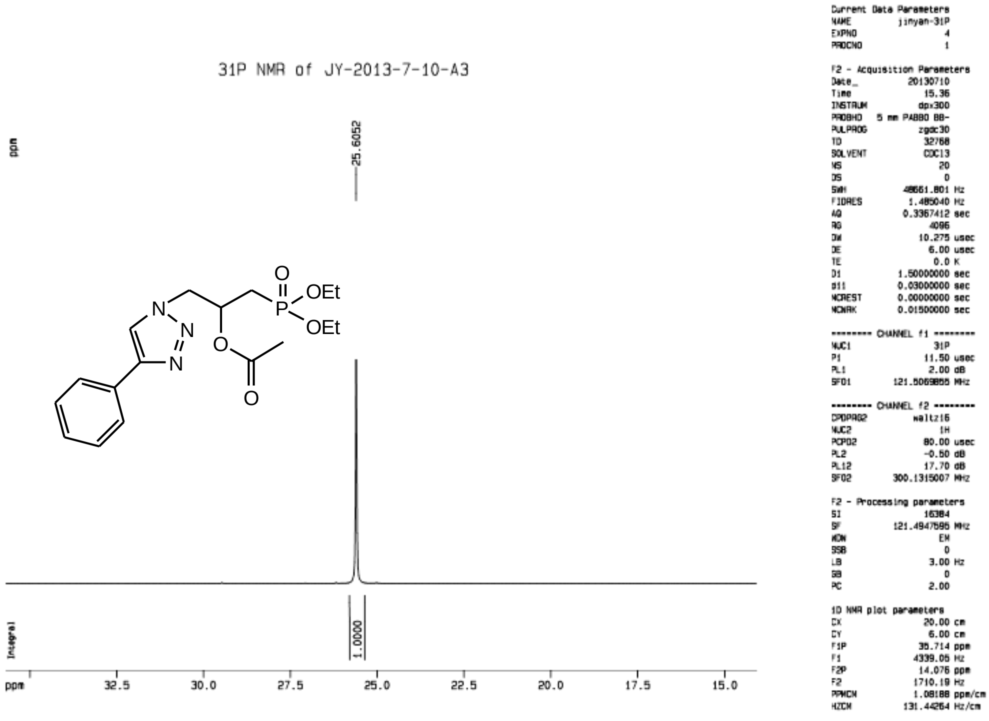

Figure S19. HRMS spectrum of compound **5-A4**.

5-A4 [M+H]382.1526338

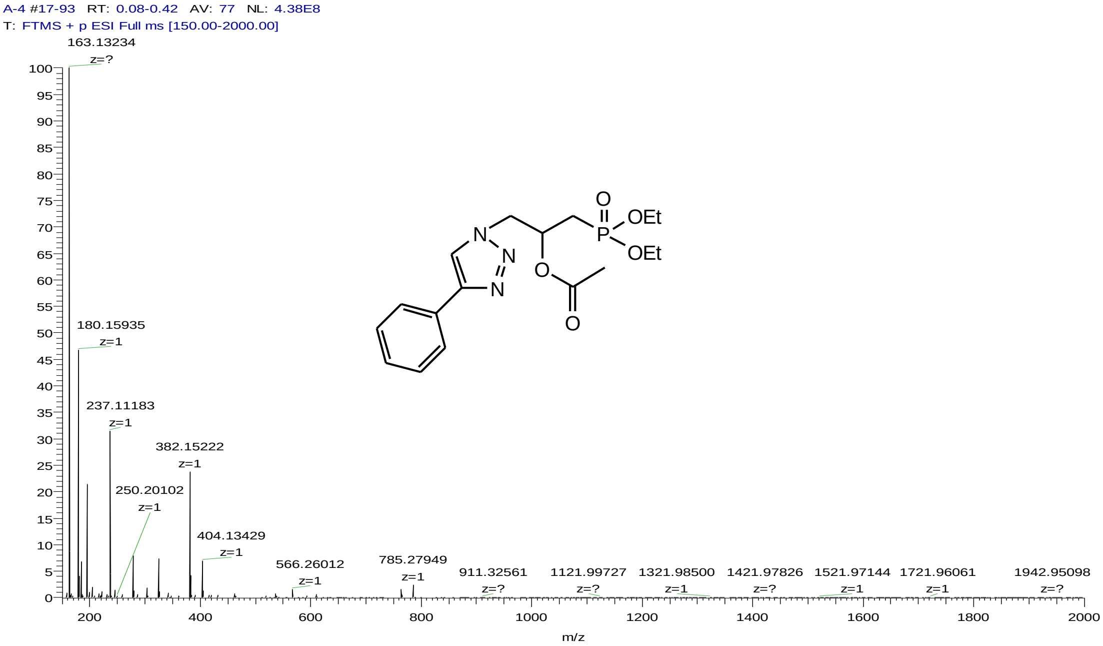

Figure S20. HRMS spectrum of compound **5-A4**.

5-A4 [M+H]382.1526338

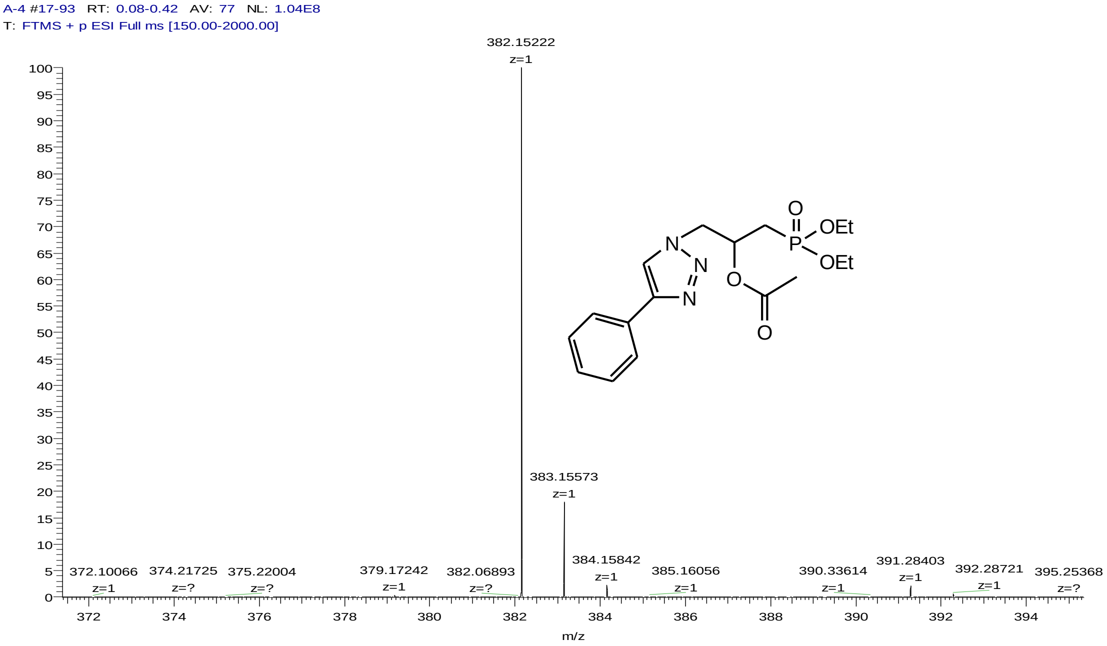

Figure S21. ^{1}H-NMR spectrum of compound **5-A5**.

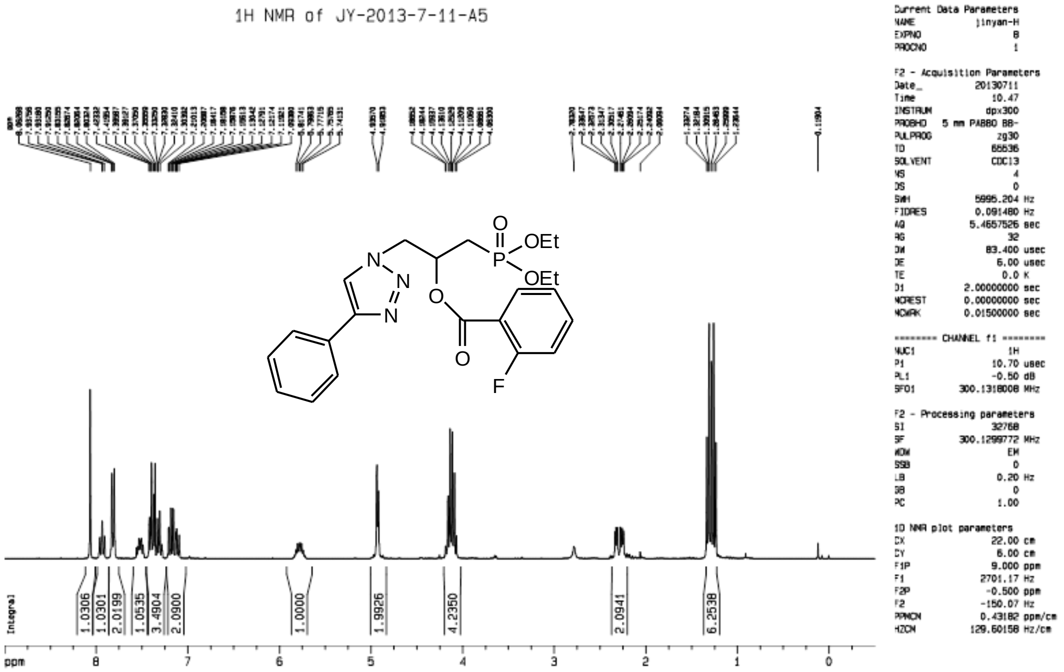

Figure S22. ^{13}C-NMR spectrum of compound **5-A5**.

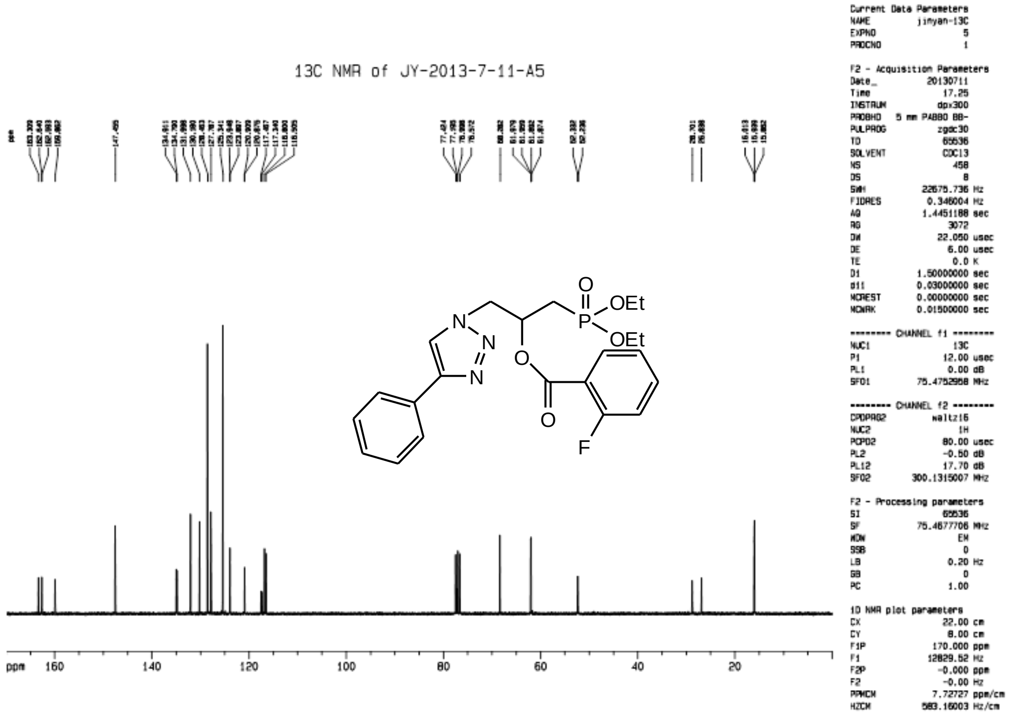

Figure S23. ^{31}P-NMR spectrum of compound **5-A5**.

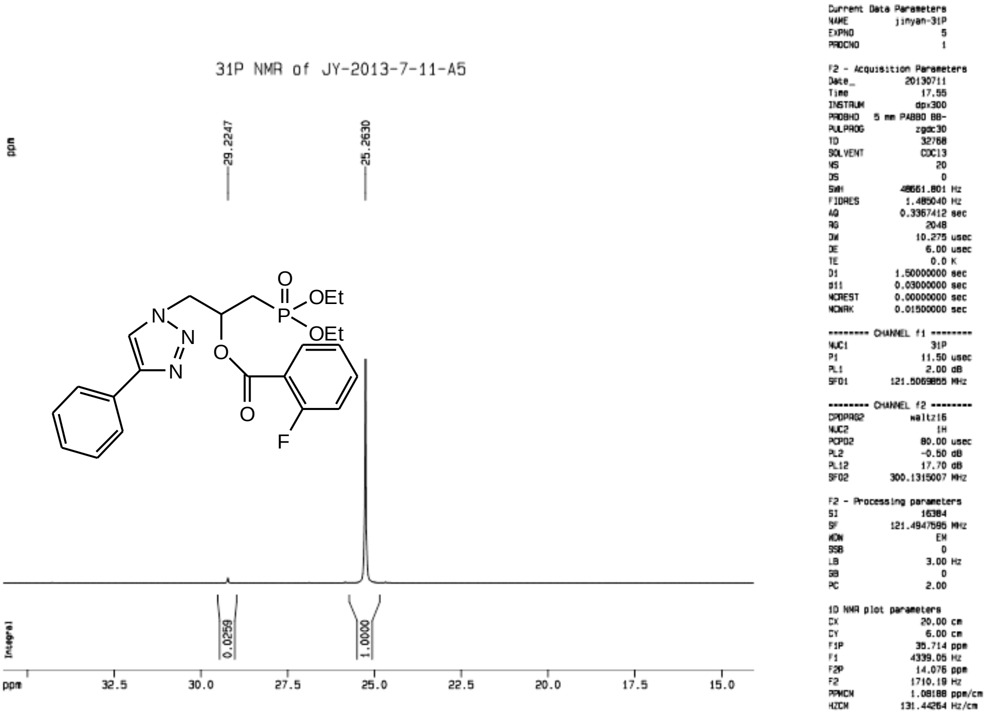

Figure S24. HRMS spectrum of compound **5-A5**.

5-A5 [M+H]462.1588621

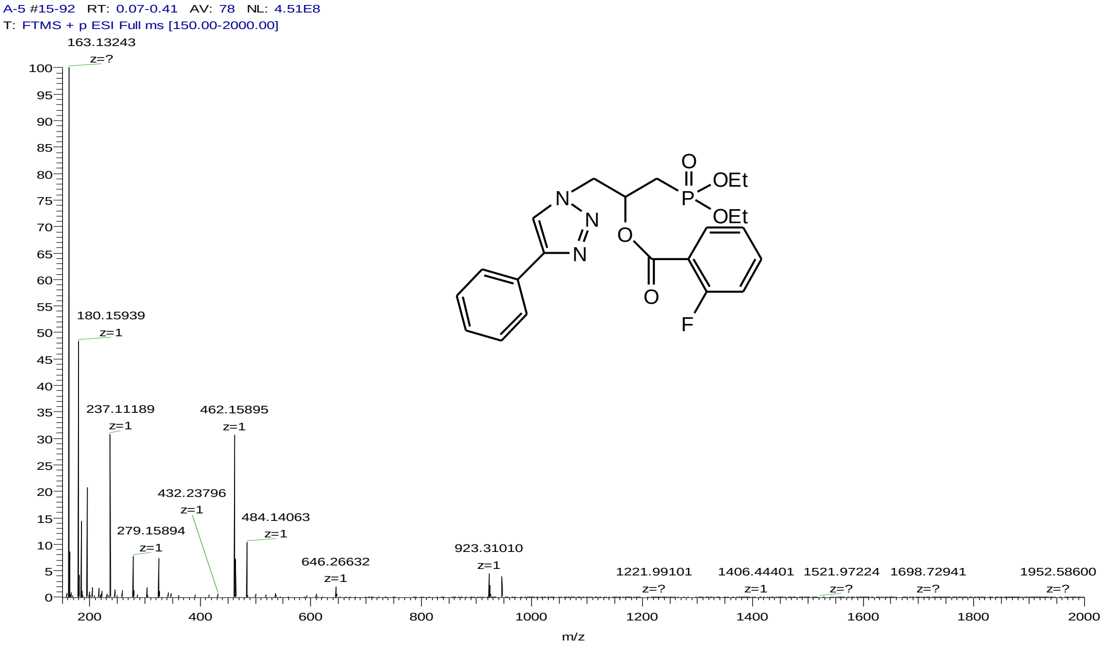

Figure S25. HRMS spectrum of compound **5-A5**.

5-A5 [M+H]462.1588621

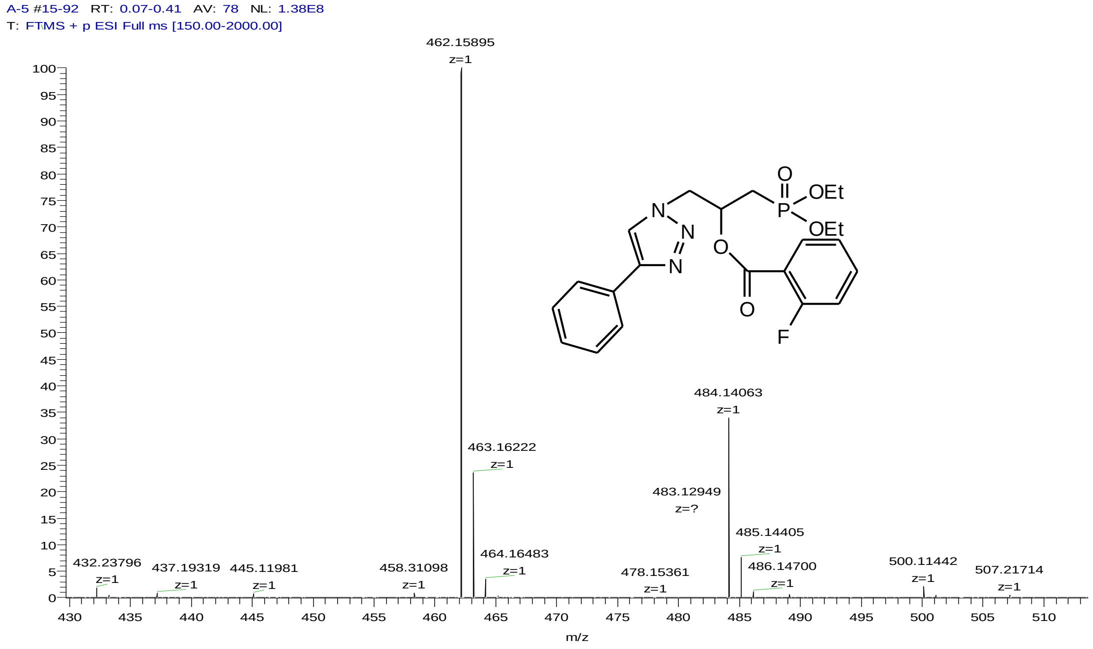

Figure S26. ^{1}H-NMR spectrum of compound **5-A6**.

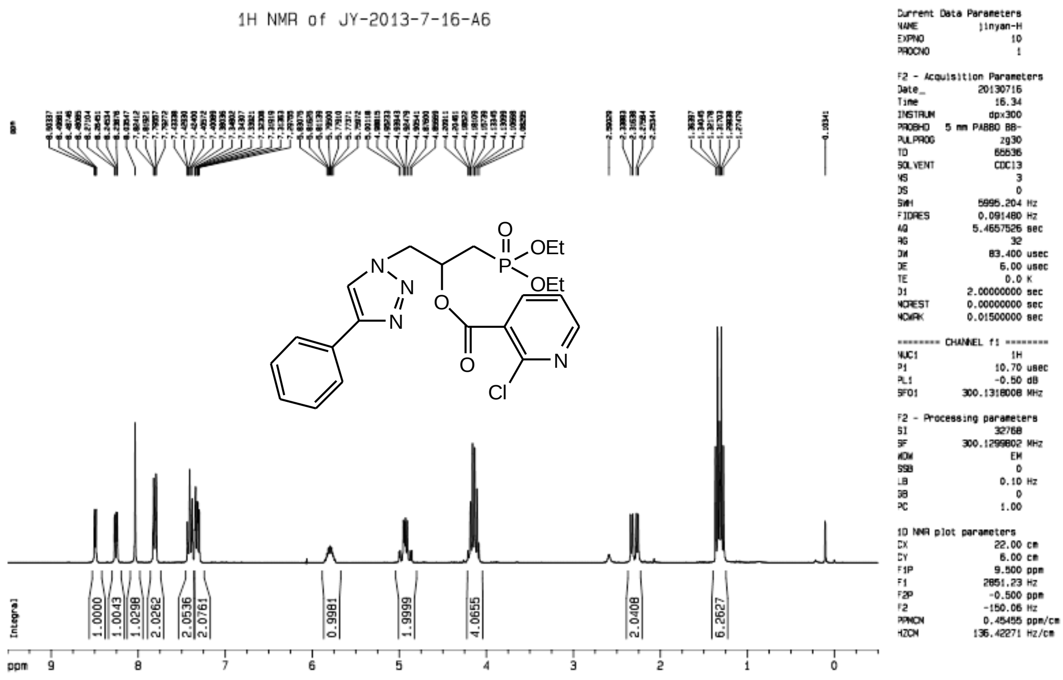

Figure S27. ^{13}C-NMR spectrum of compound **5-A6**.

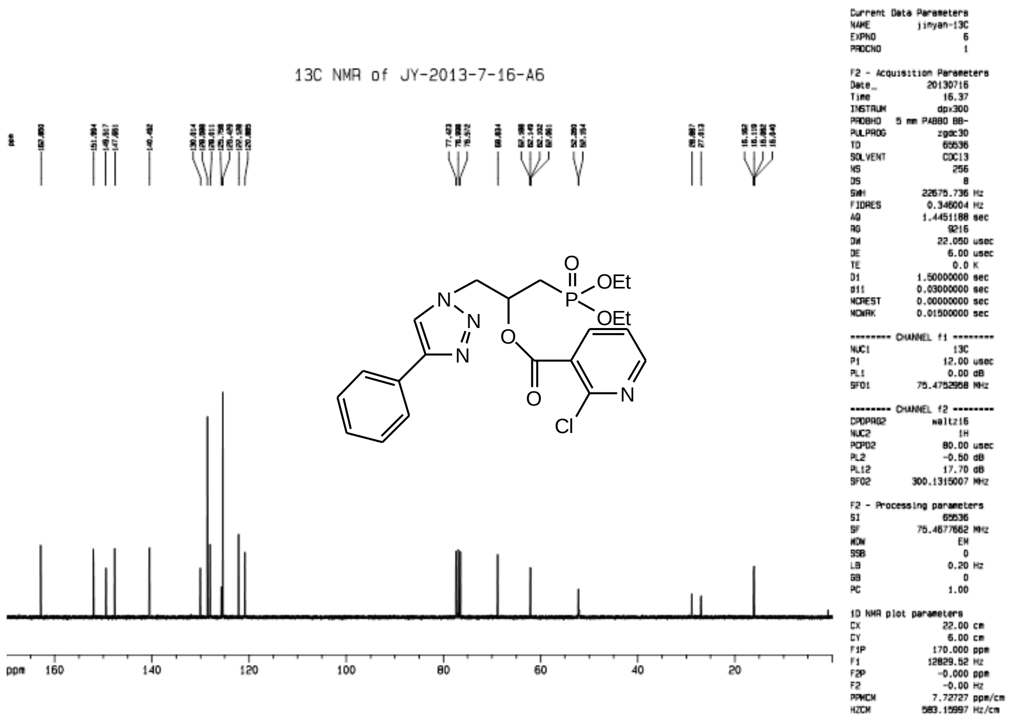

Figure S28. ^{31}P-NMR spectrum of compound **5-A6**.

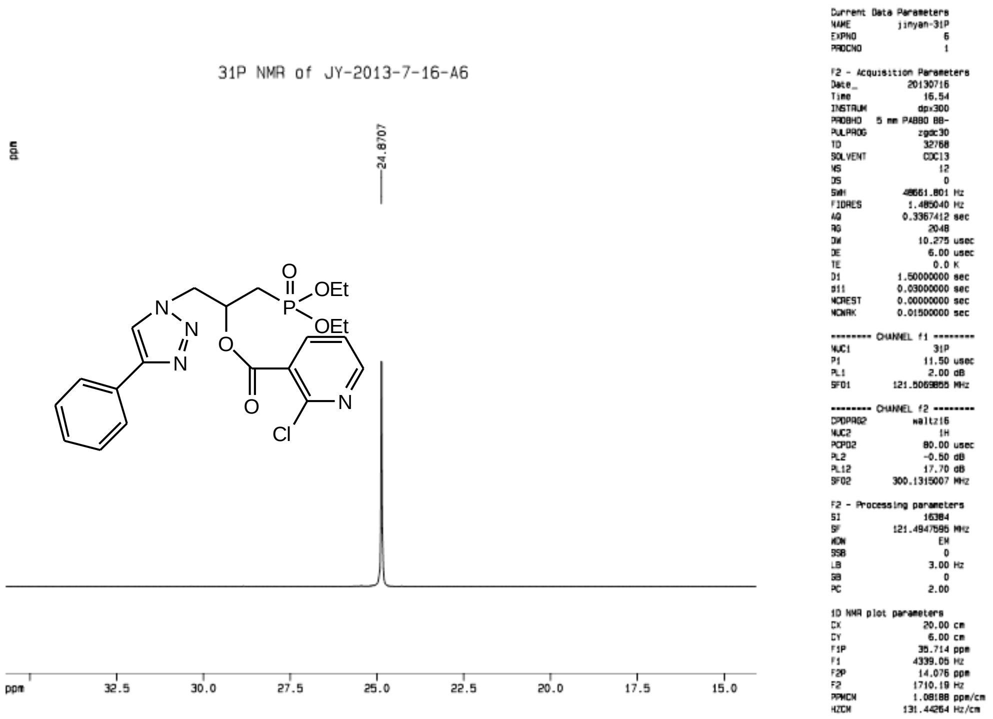

Figure S29. HRMS spectrum of compound **5-A6**.

5-A6 [M+H]479.1245606

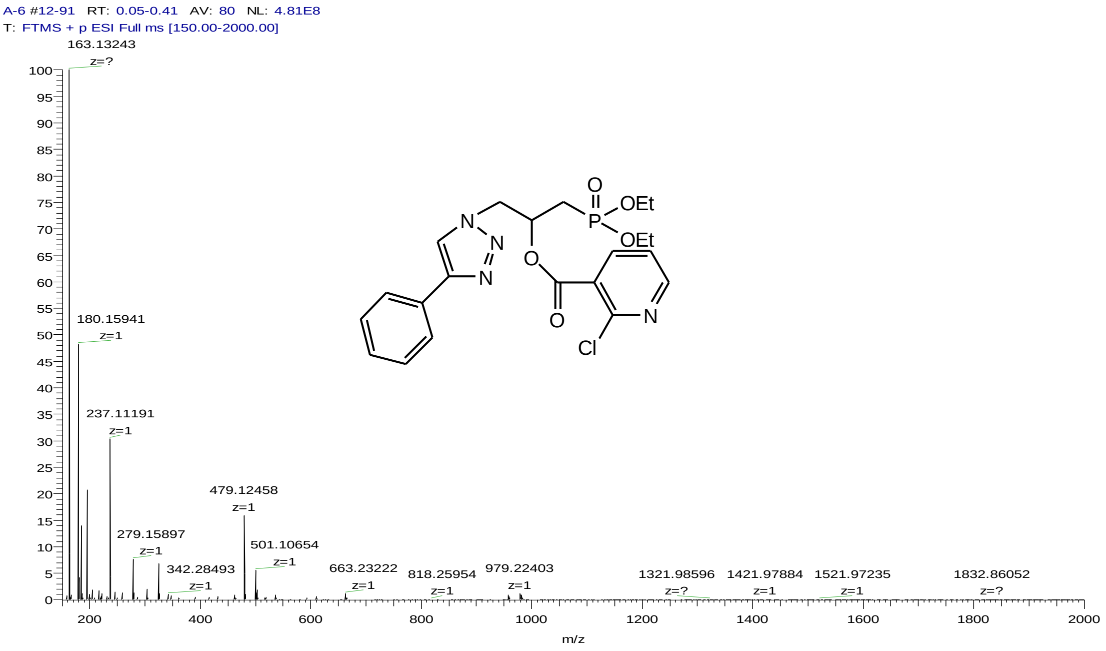

Figure S30. HRMS spectrum of compound **5-A6**.

5-A6 [M+H]479.1245606

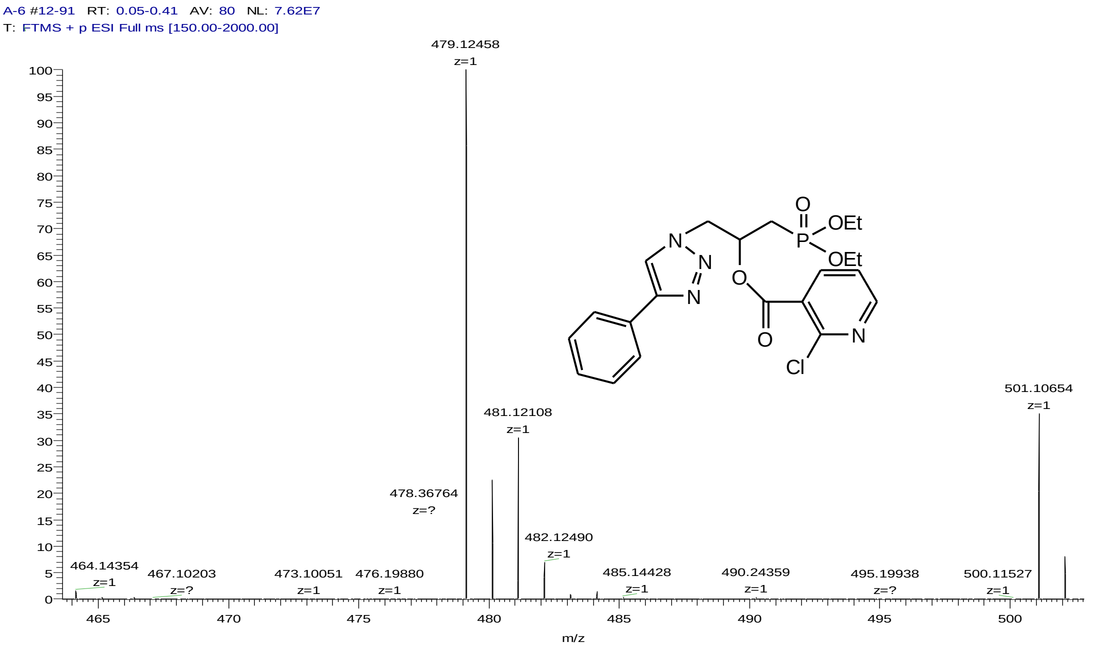

Figure S31. ^{1}H-NMR spectrum of compound **5-A7**.

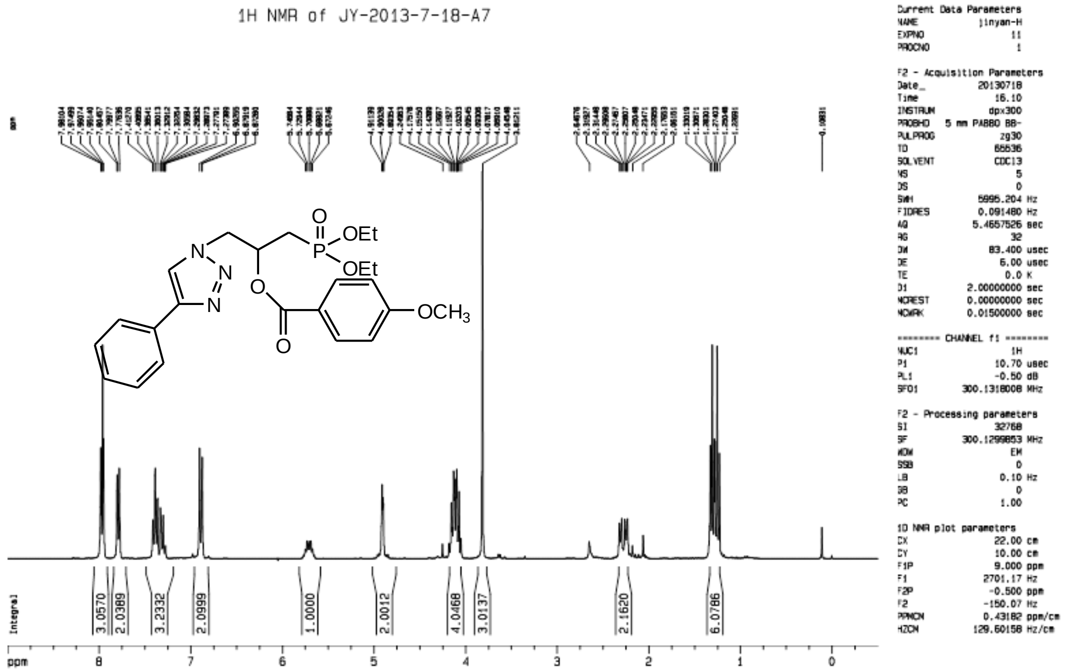

Figure S32. ^{13}C-NMR spectrum of compound **5-A7**.

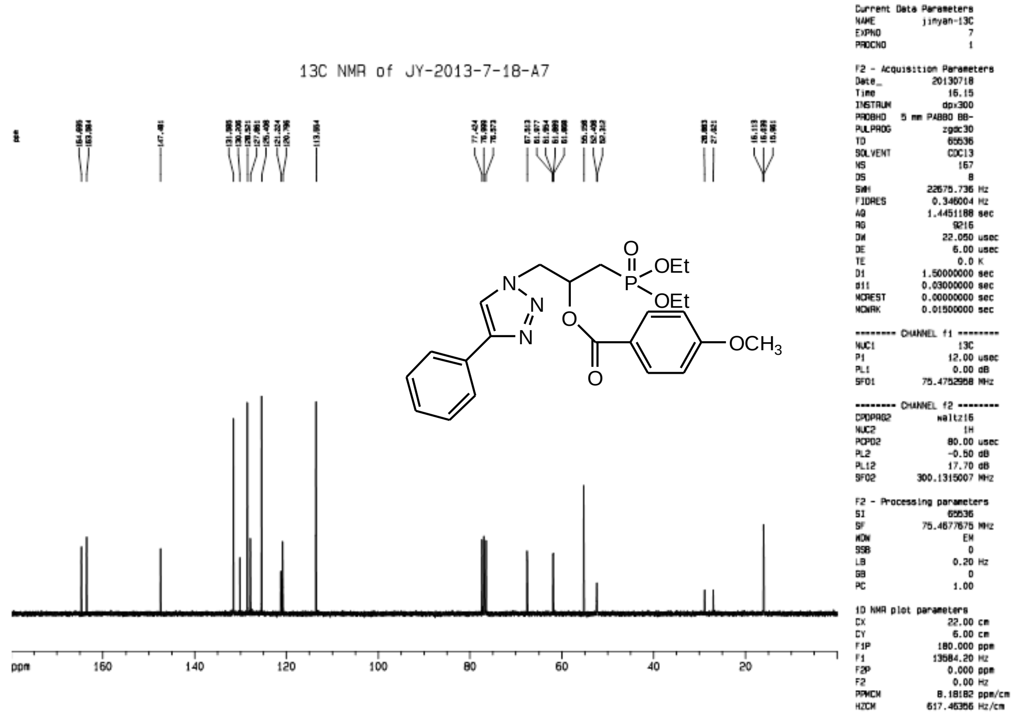

Figure S33. ^{31}P-NMR spectrum of compound **5-A7**.

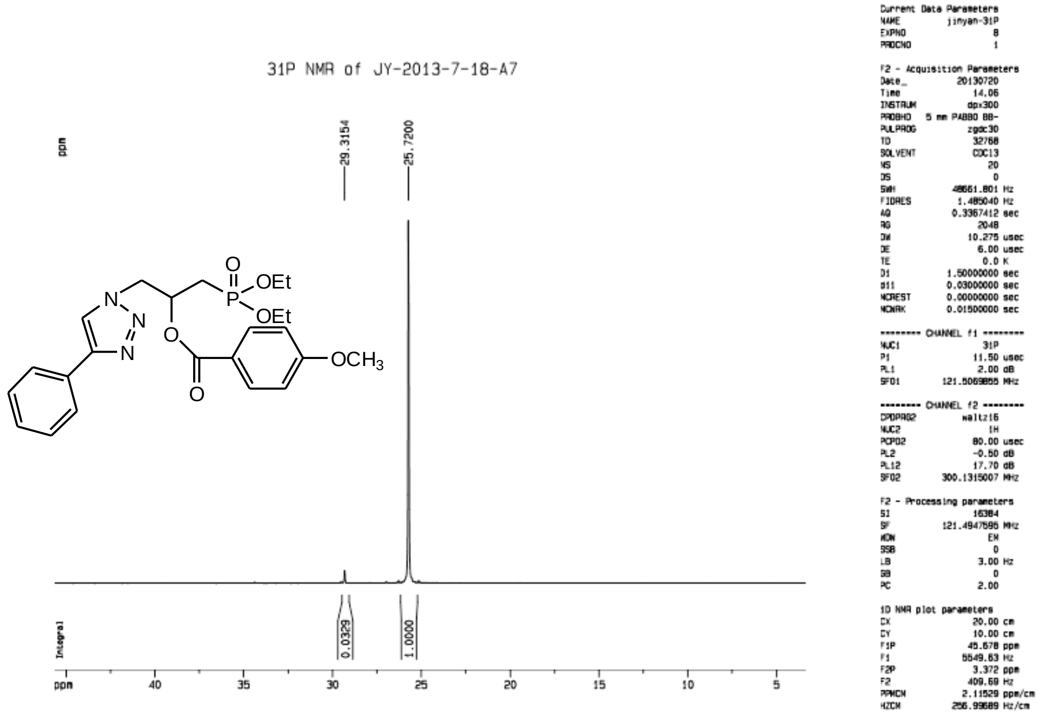

Figure S34. HRMS spectrum of compound **5-A7**.

5-A7 [M+H]474.1788486

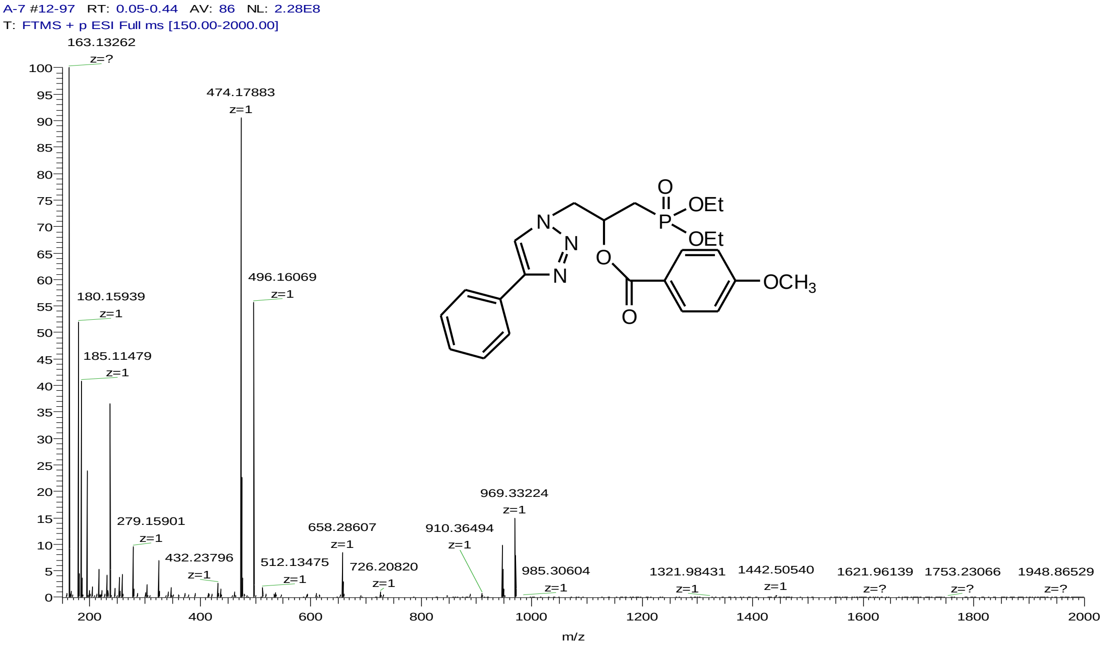

Figure S35. HRMS spectrum of compound **5-A7**.

5-A7 [M+H]474.1788486

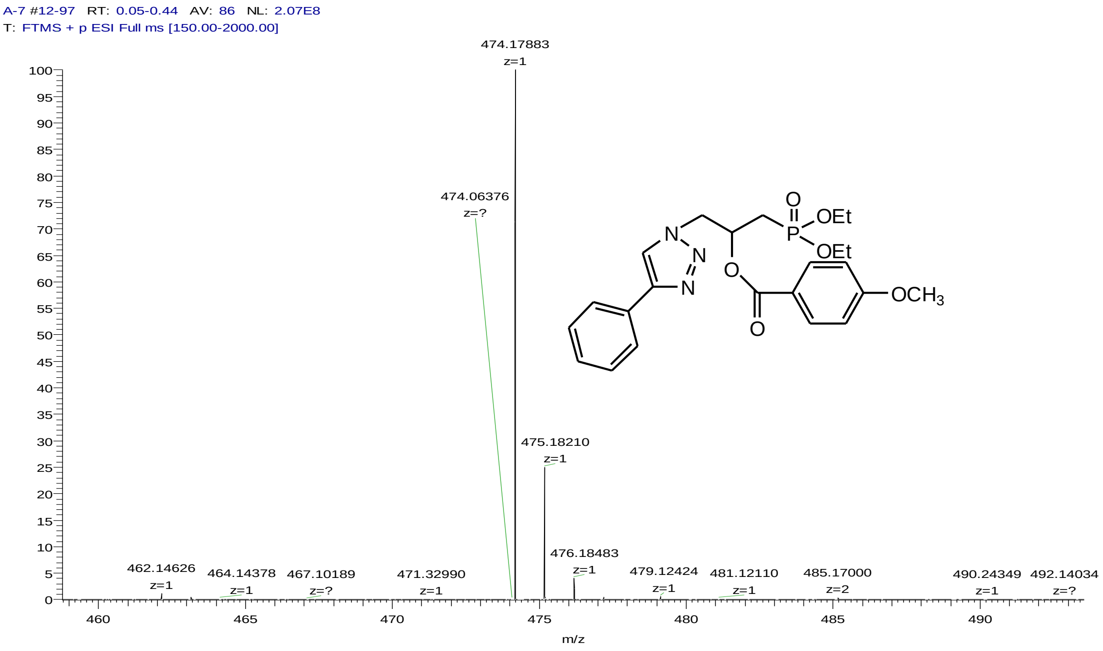

Figure S36. ^{1}H-NMR spectrum of compound **5-A8**.

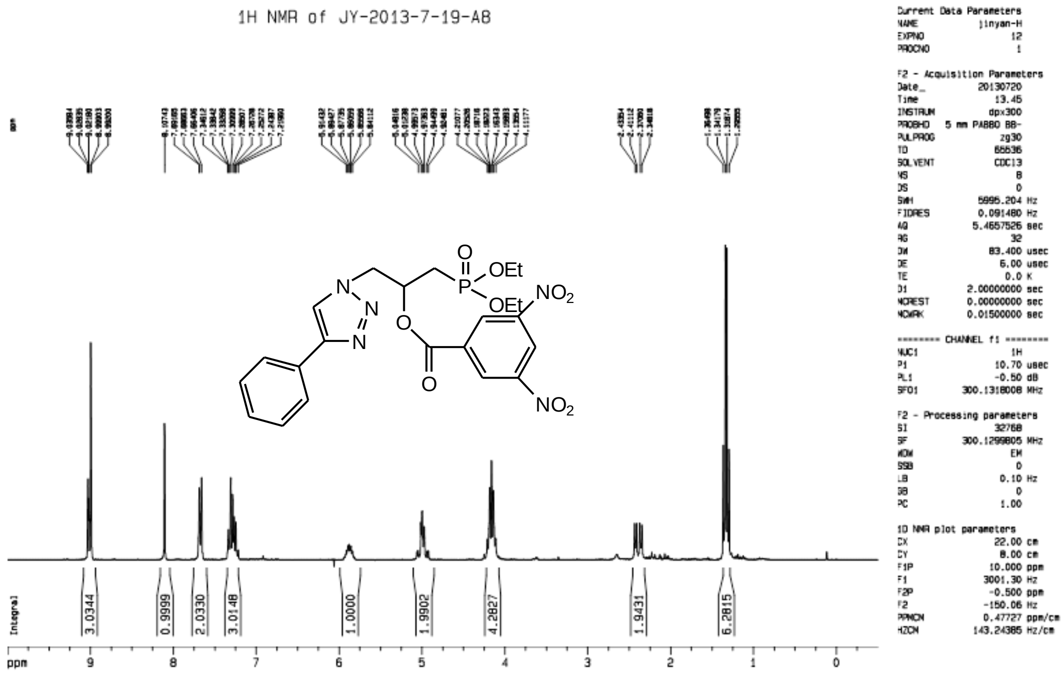

Figure S37. ^{13}C-NMR spectrum of compound **5-A8**.

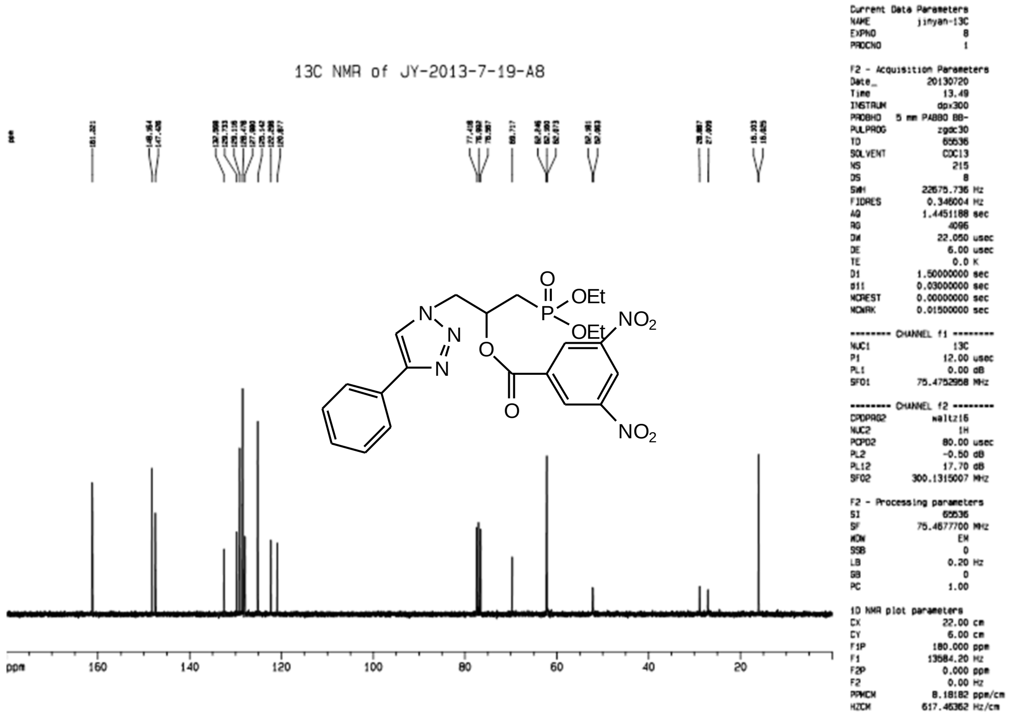

Figure S38. ^{31}P-NMR spectrum of compound **5-A8**.

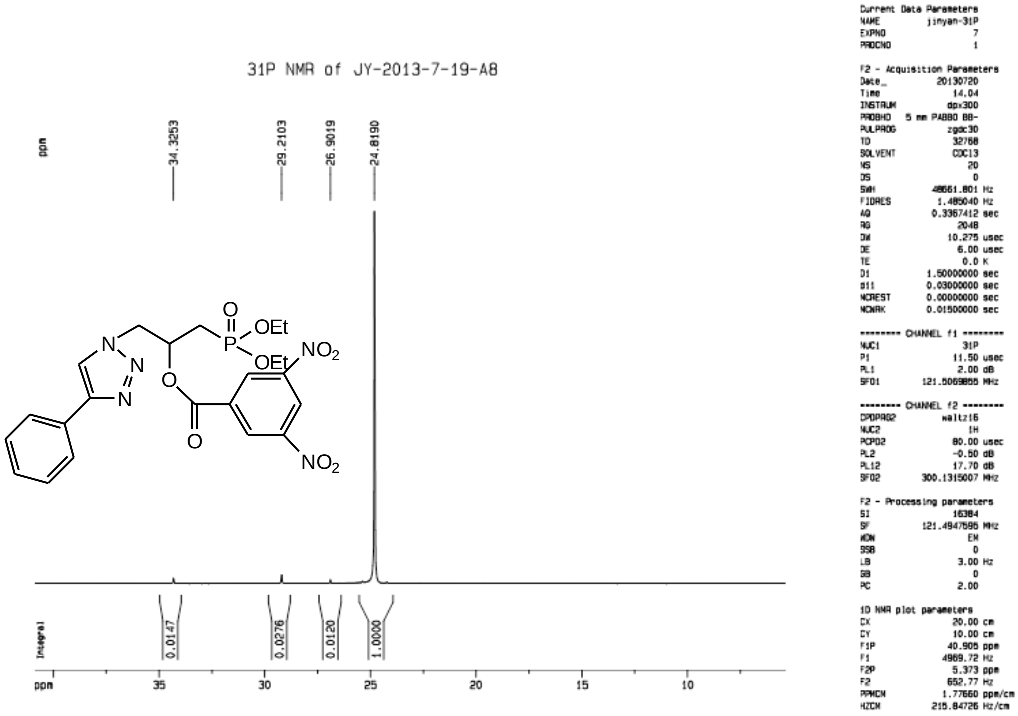

Figure S39. HRMS spectrum of compound **5-A8**

5-A8 [M+H]534.1384403

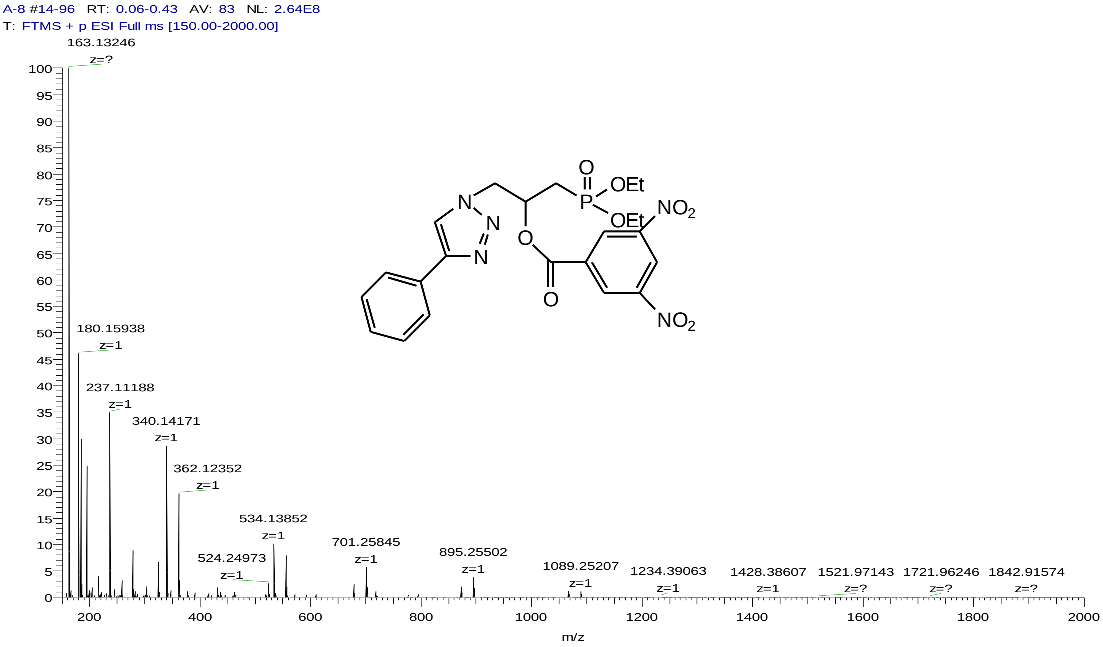

Figure S40. HRMS spectrum of compound **5-A8**

5-A8 [M+H]534.1384403

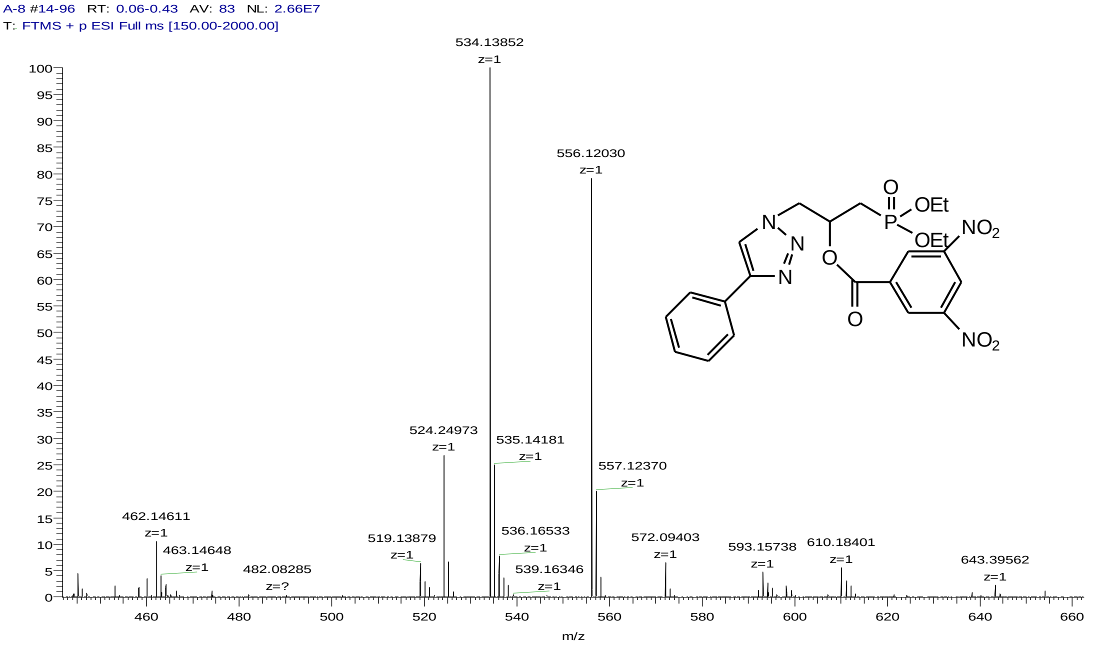

Figure S41. ^{1}H-NMR spectrum of compound **5-B1**.

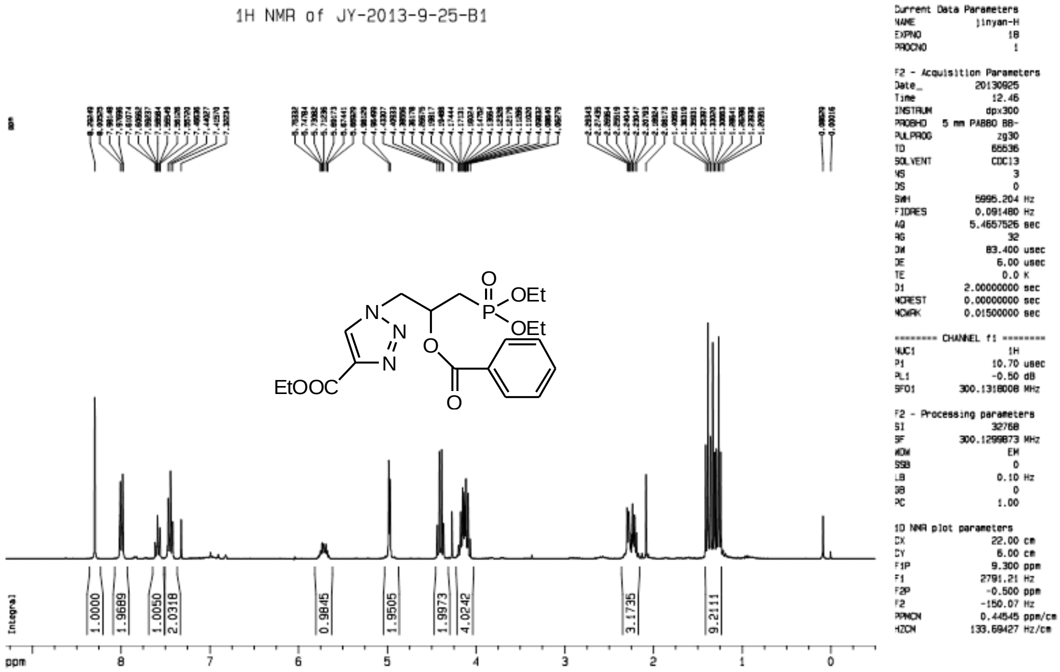

Figure S42. ^{13}C-NMR spectrum of compound **5-B1**.

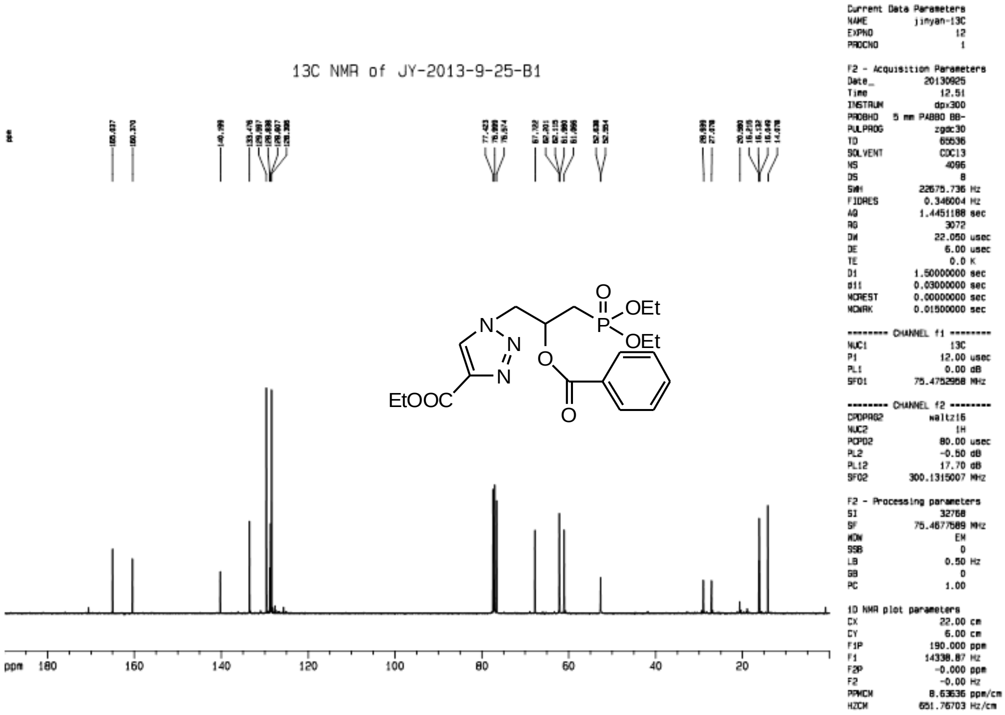

Figure S43. ^{31}P-NMR spectrum of compound **5-B1**.

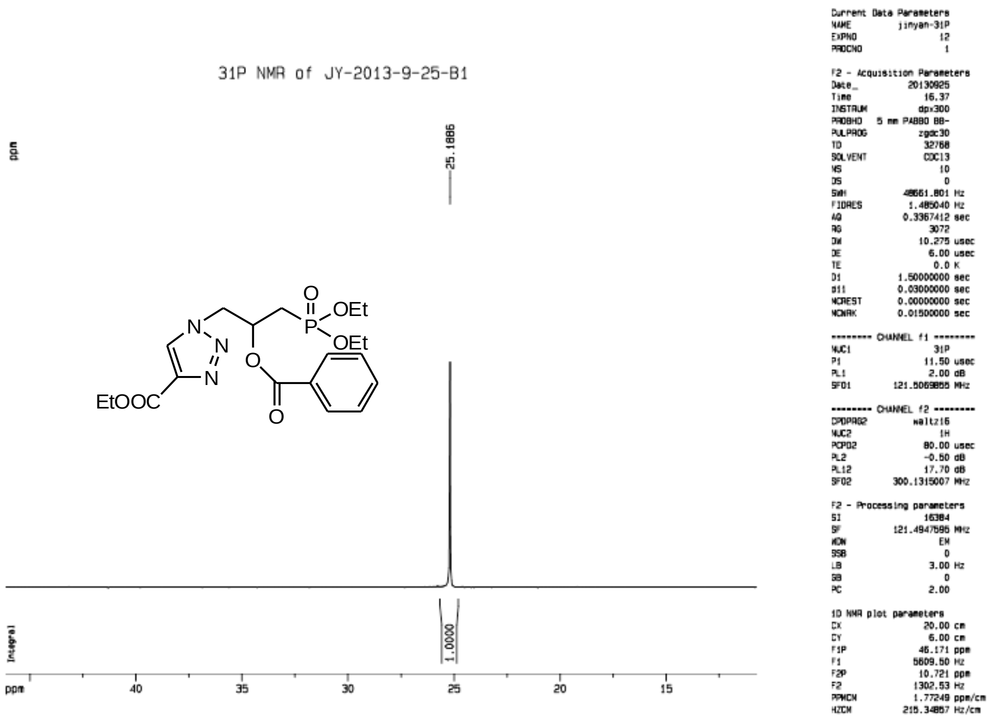

Figure S44. HRMS spectrum of compound **5-B1**.

5-B1 [M+H]440.1581131

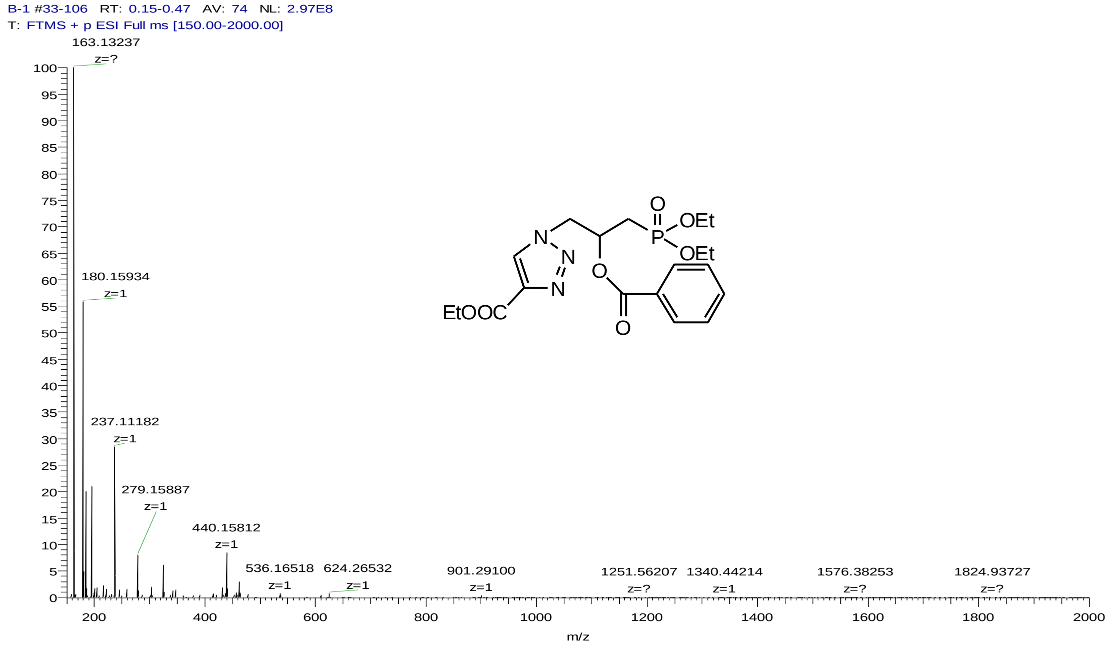

Figure S45. HRMS spectrum of compound **5-B1**.

5-B1 [M+H]440.1581131

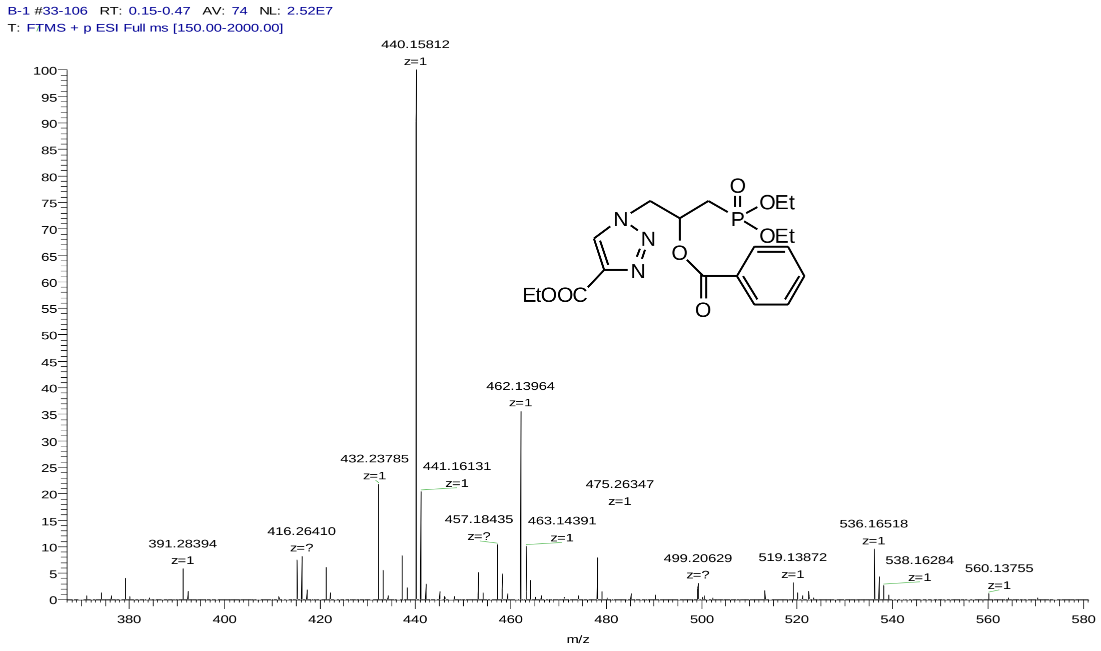

Figure S46. ^{1}H-NMR spectrum of compound **5-B2**.

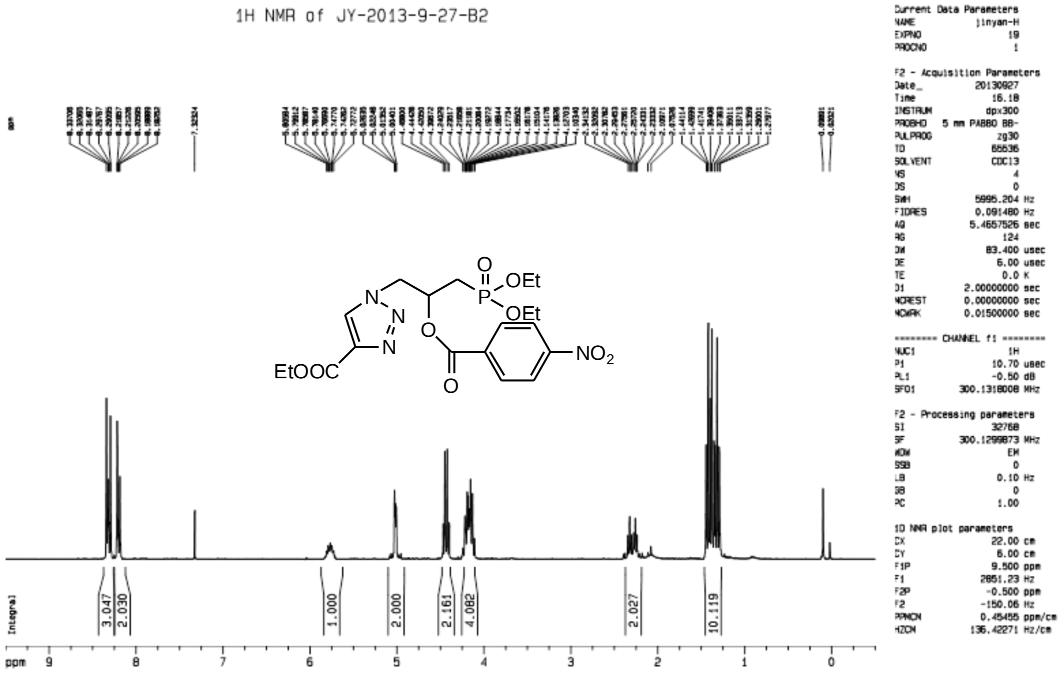

Figure S47. ^{13}C-NMR spectrum of compound **5-B2**.

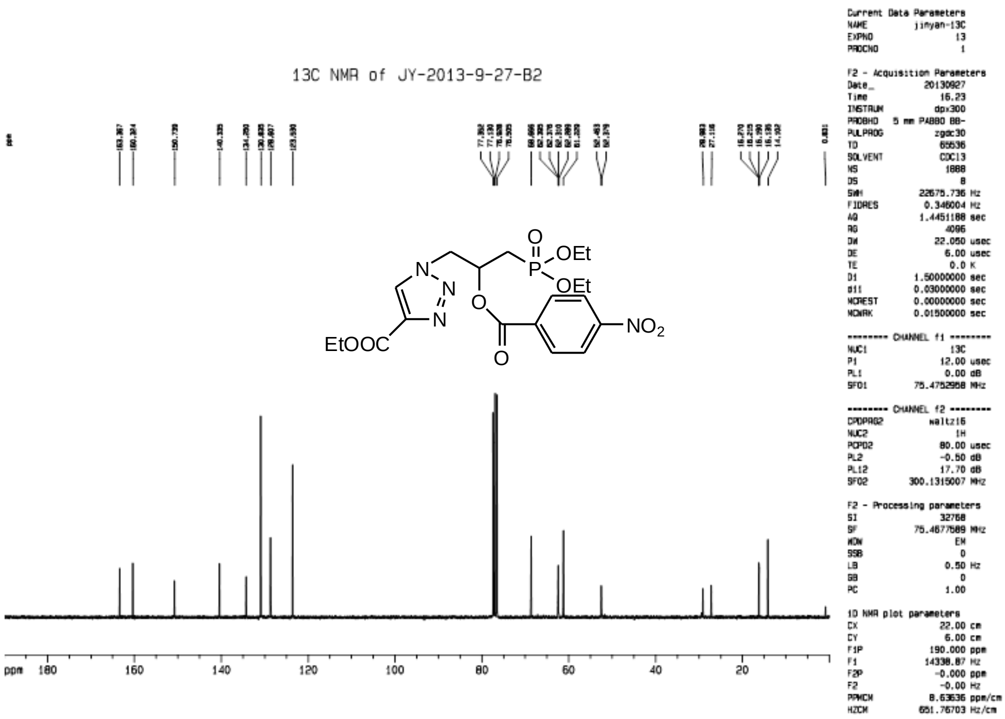

Figure S48. ^{31}P-NMR spectrum of compound **5-B2**.

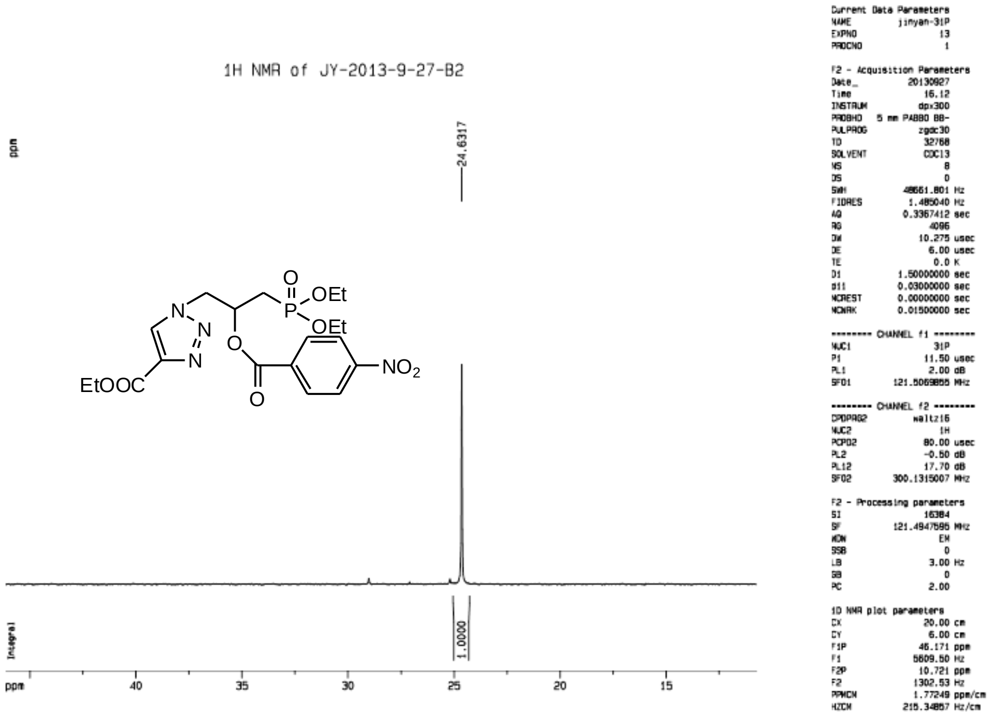

Figure S49. HRMS spectrum of compound **5-B2**.

5-B2 [M+H] 485.1431913

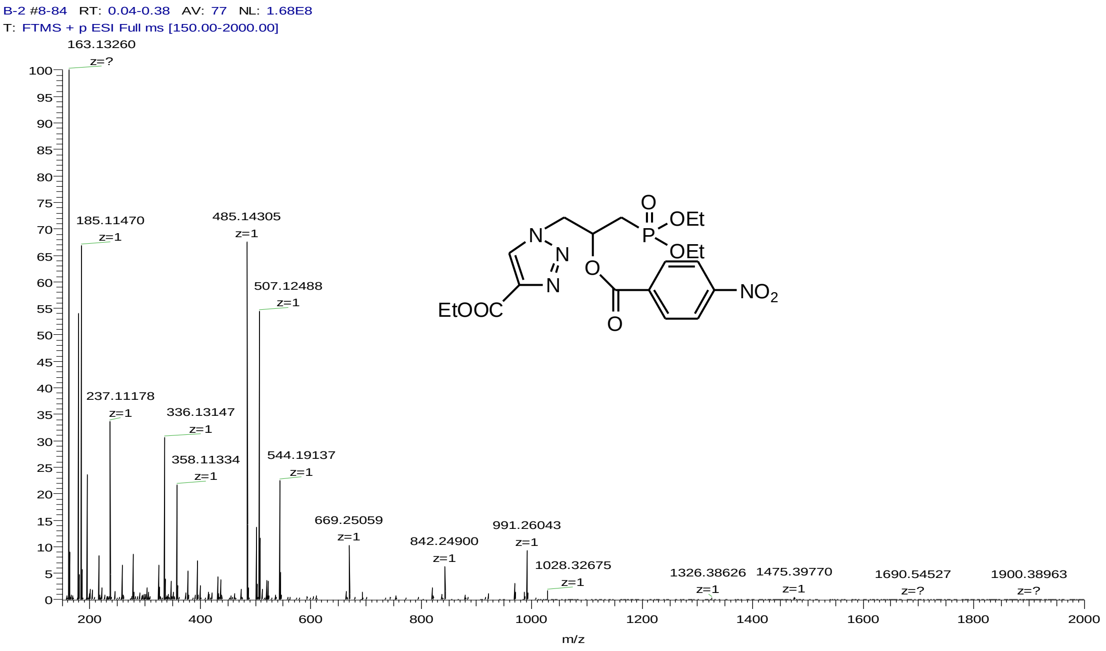

Figure S50. HRMS spectrum of compound **5-B2**.

5-B2 [M+H] 485.1431913

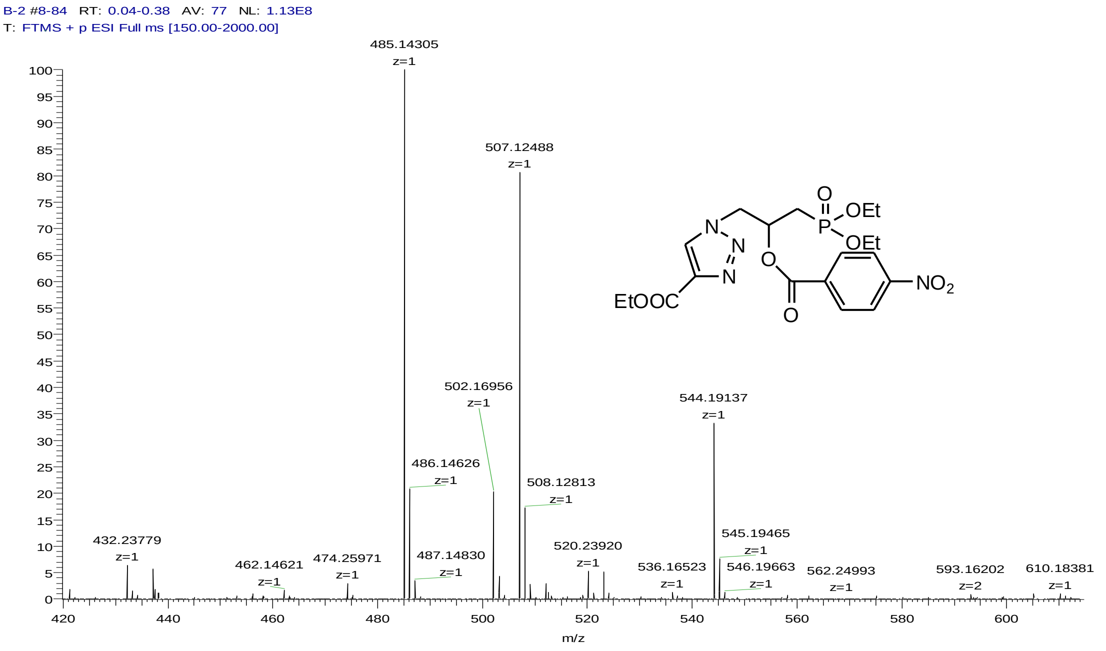

Figure S51. ^{1}H-NMR spectrum of compound **5-B3.**

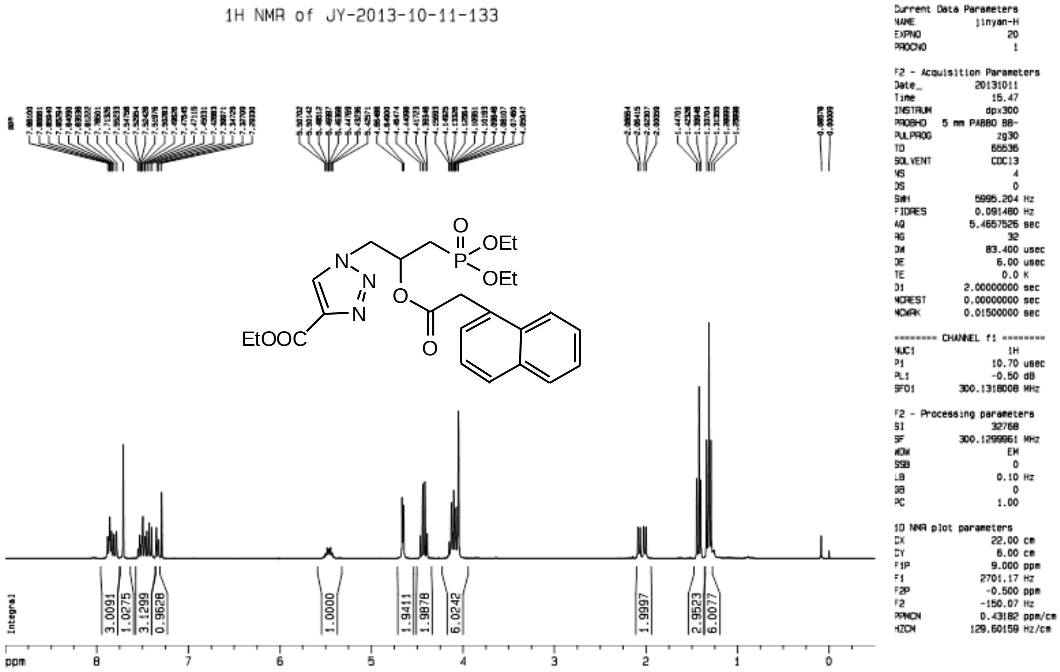

Figure S52. ^{13}C-NMR spectrum of compound **5-B3**.

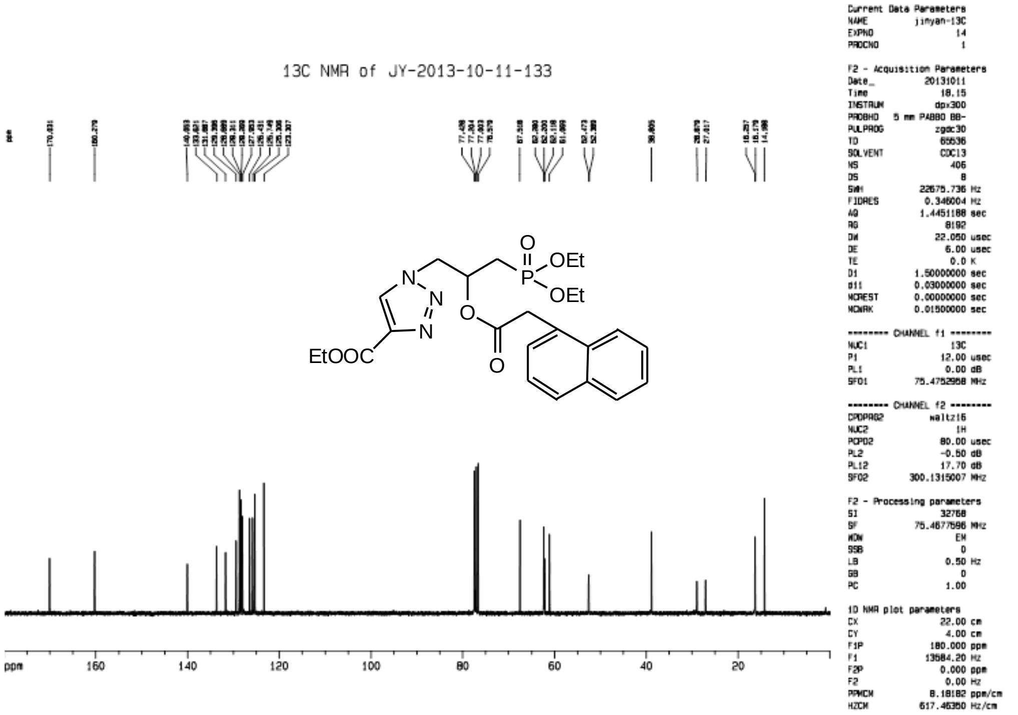

Figure S53. ^{31}P-NMR spectrum of compound **5-B3**.

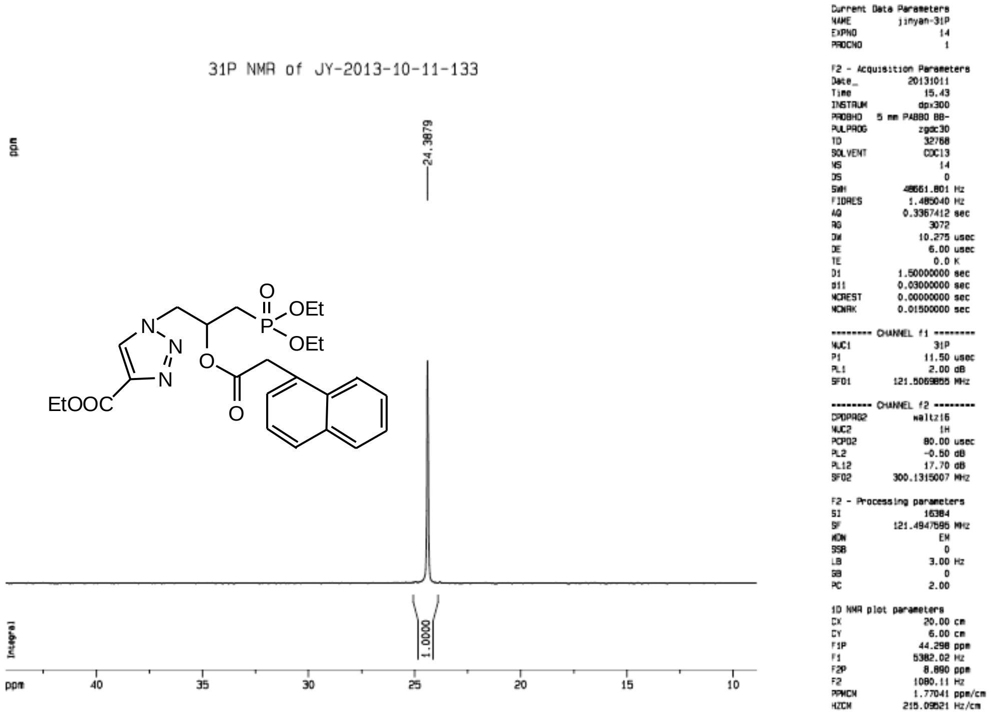

Figure S54. HRMS spectrum of compound **5-B3**.

5-B3 [M+H]504.1894132

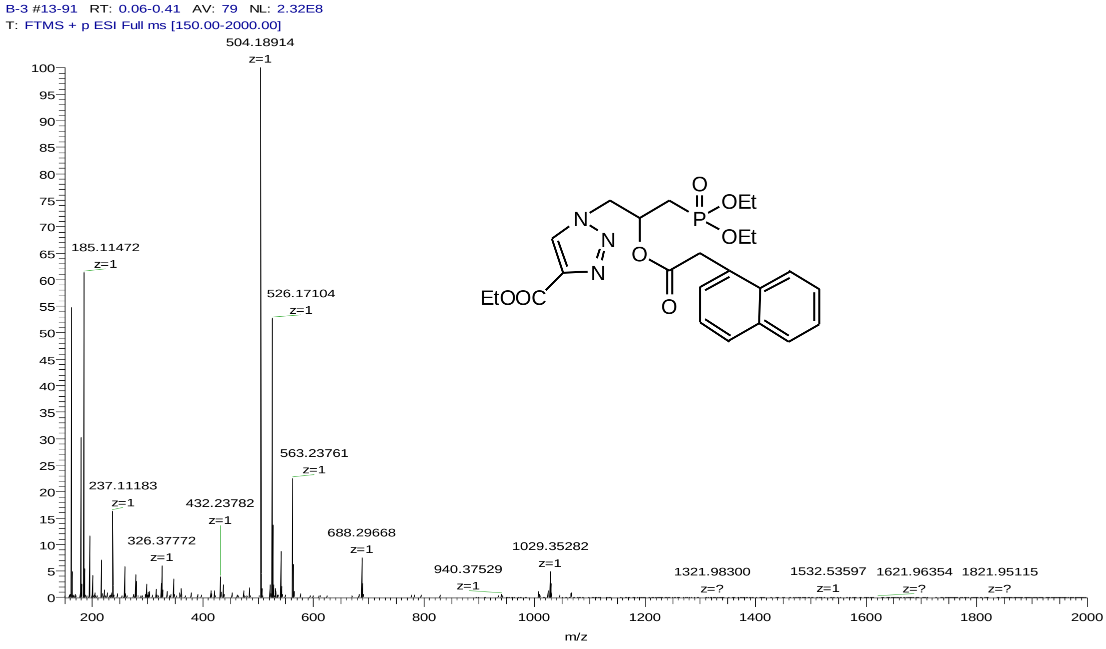

Figure S55. HRMS spectrum of compound **5-B3**.

5-B3 [M+H]504.1894132

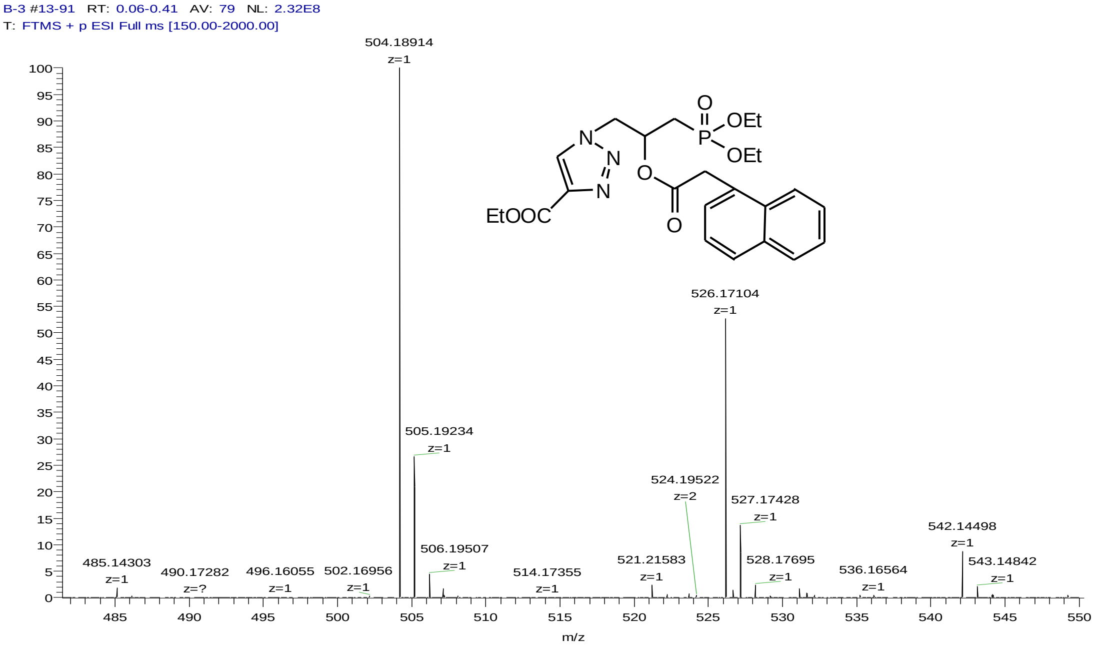

Figure S56. ^{1}H-NMR spectrum of compound **5-B4**.

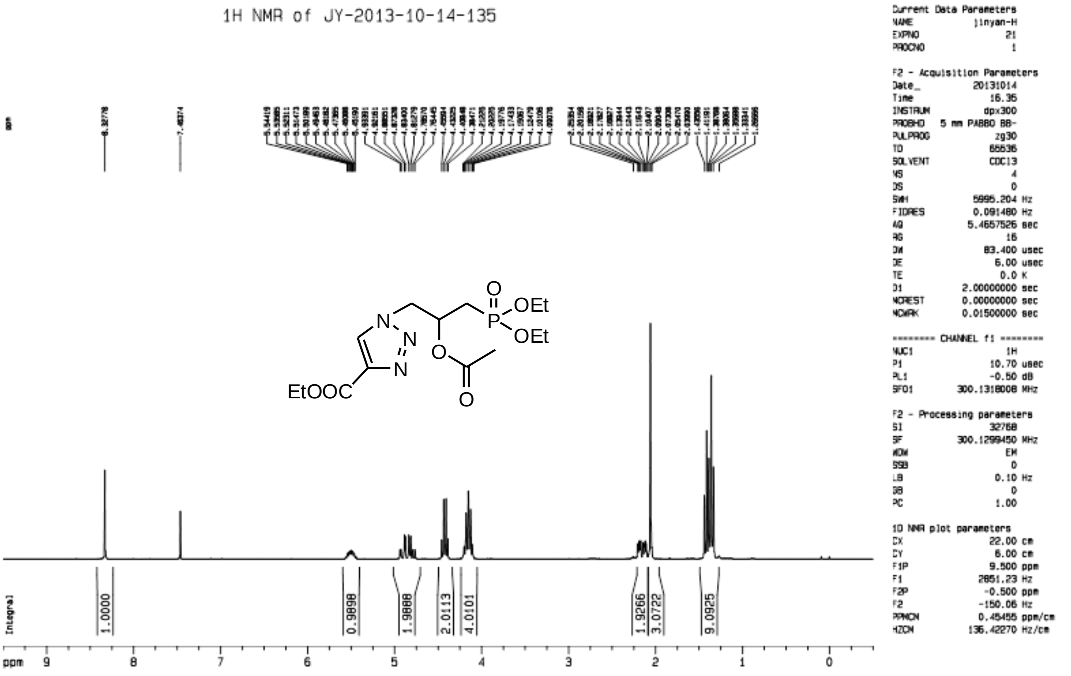

Figure S57. ^{13}C-NMR spectrum of compound **5-B4**.

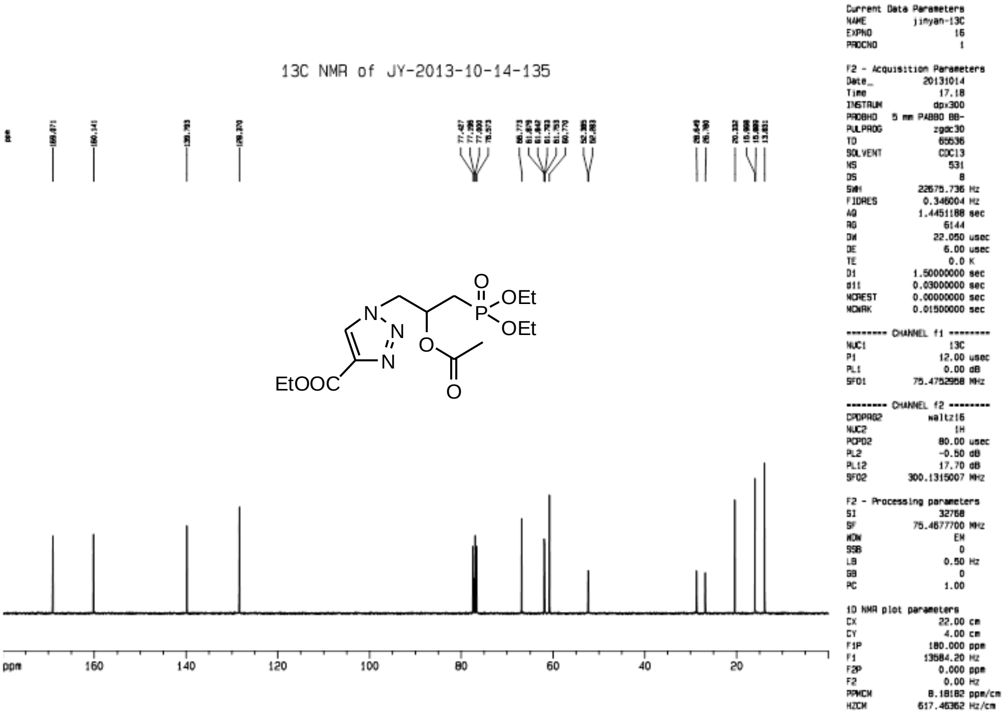

Figure S58. ^{31}P-NMR spectrum of compound **5-B4**.

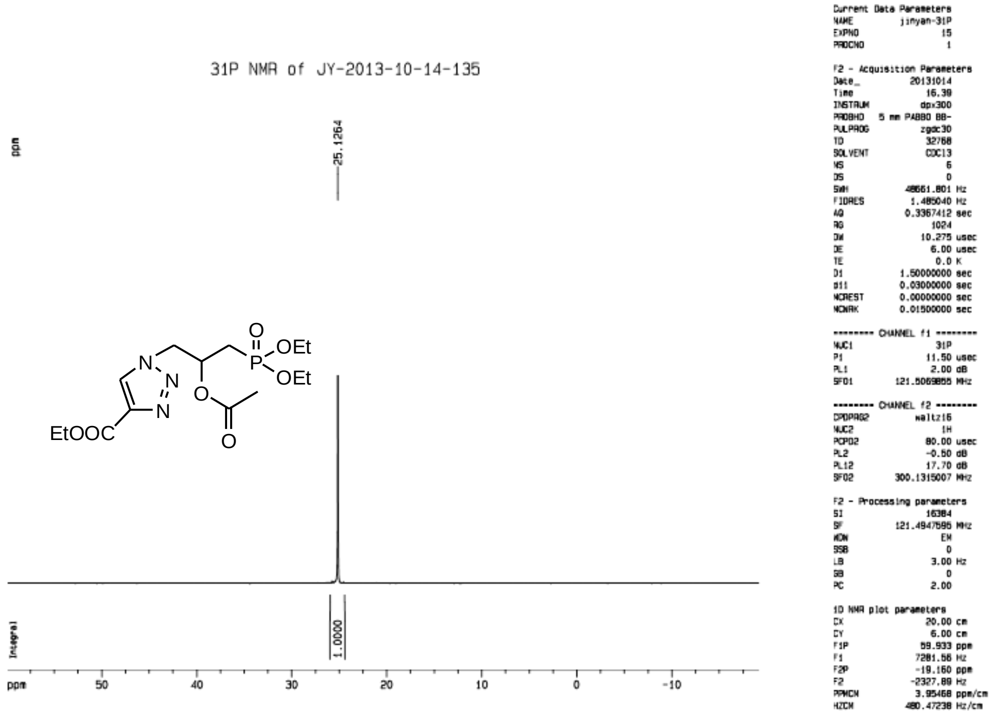

Figure S59. HRMS spectrum of compound **5-B4**

5-B4 [M+H]378.1424631

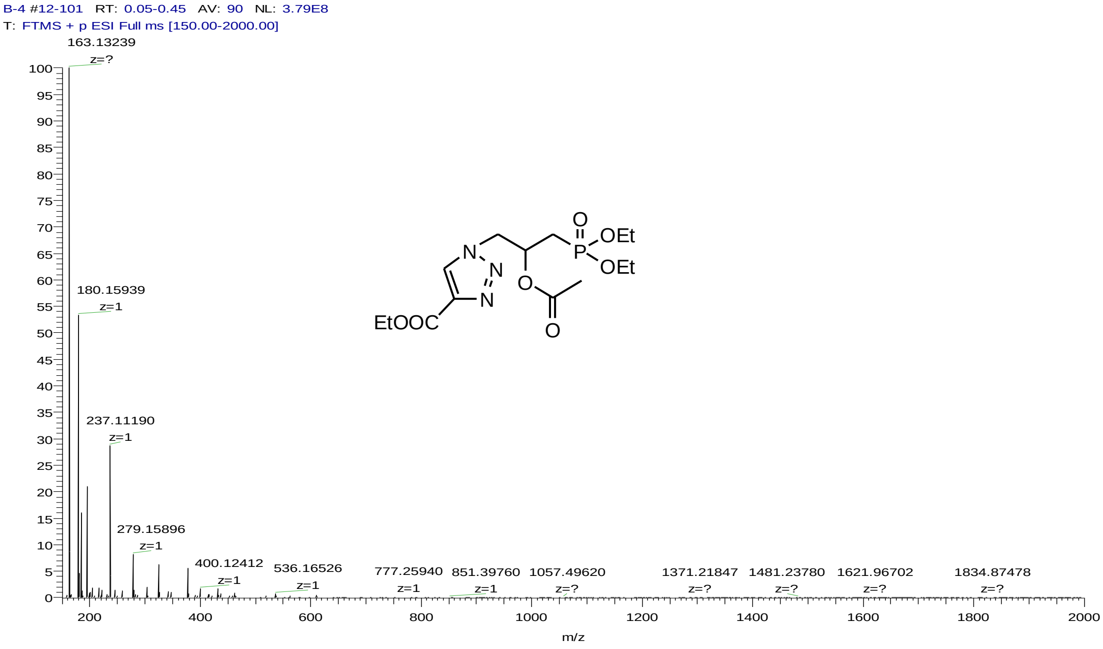

Figure S60. HRMS spectrum of compound **5-B4**.

5-B4 [M+H]378.1424631

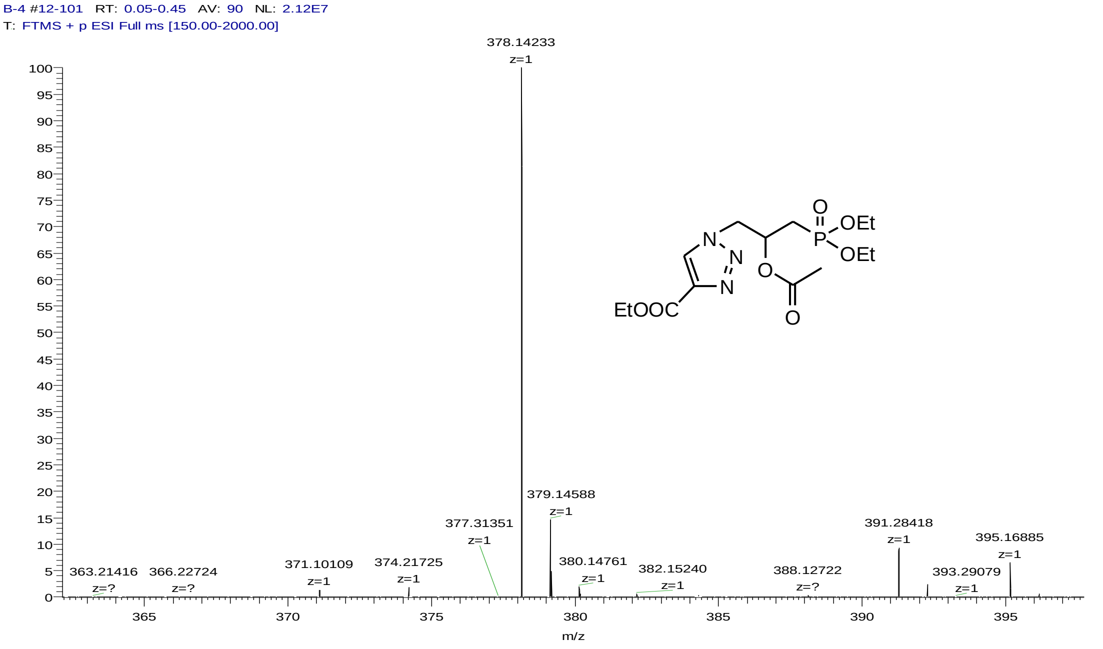

Figure S61. ^{1}H-NMR spectrum of compound **5-B5**.

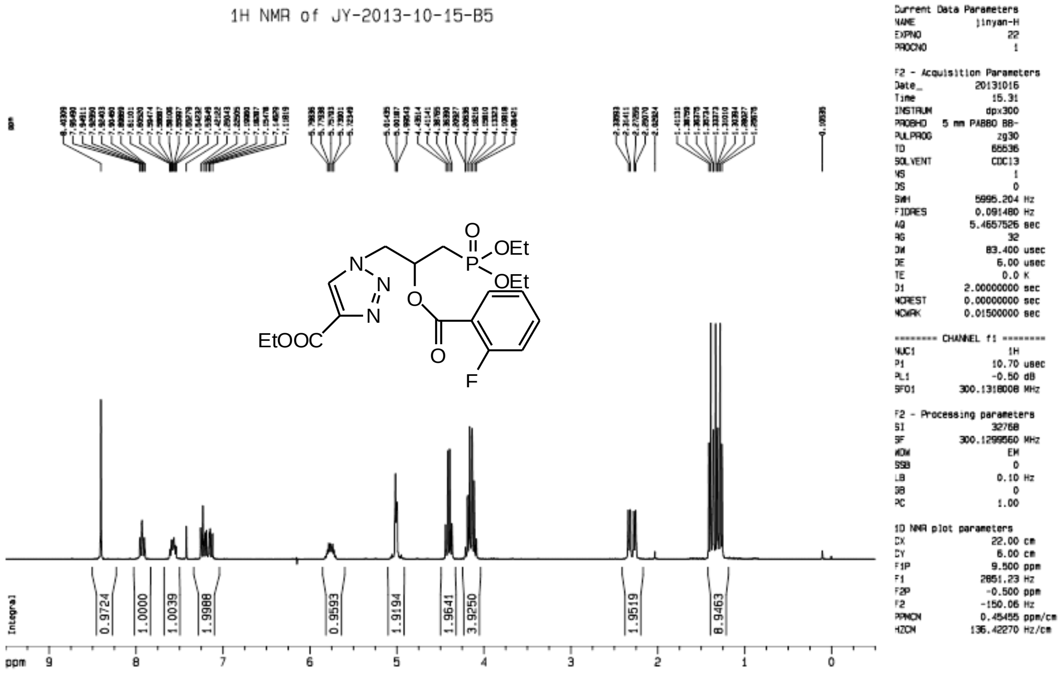

Figure S62. ^{13}C-NMR spectrum of compound **5-B5**.

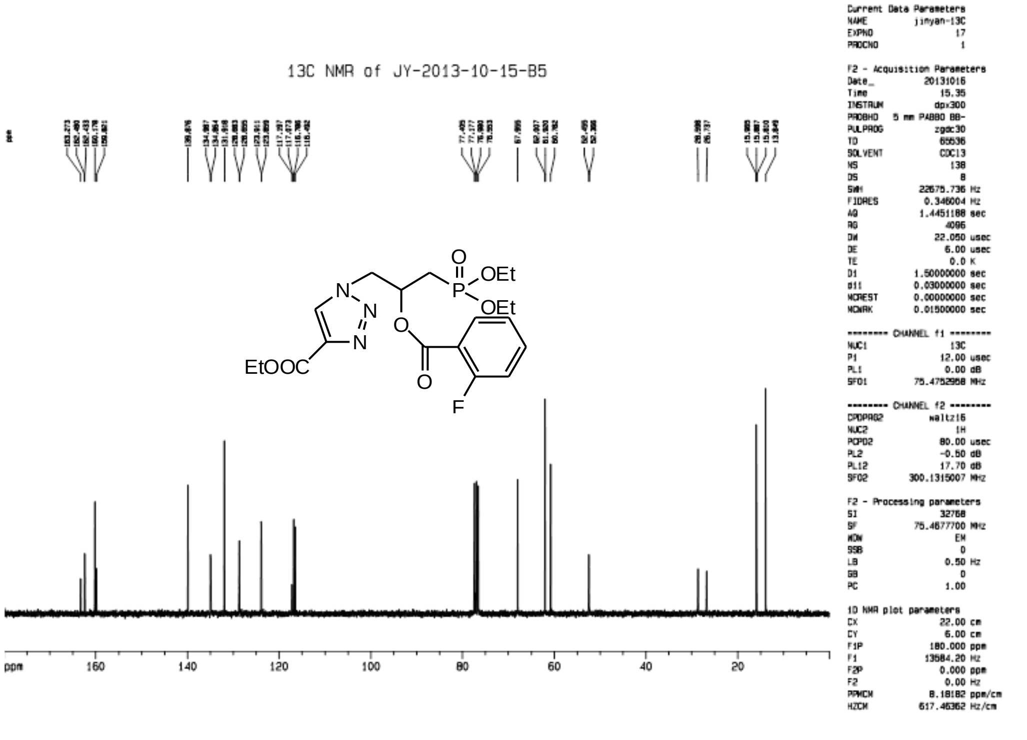

Figure S63. ^{31}P-NMR spectrum of compound **5-B5**.

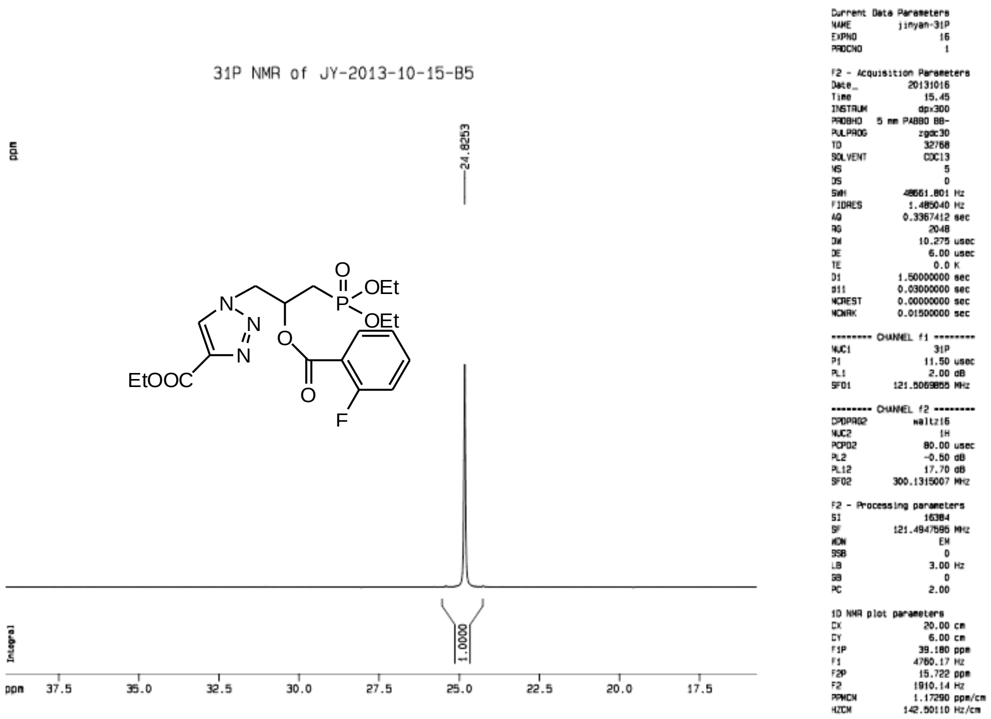

Figure S64. HRMS spectrum of compound **5-B5**.

5-B5 [M+H]458.1486913

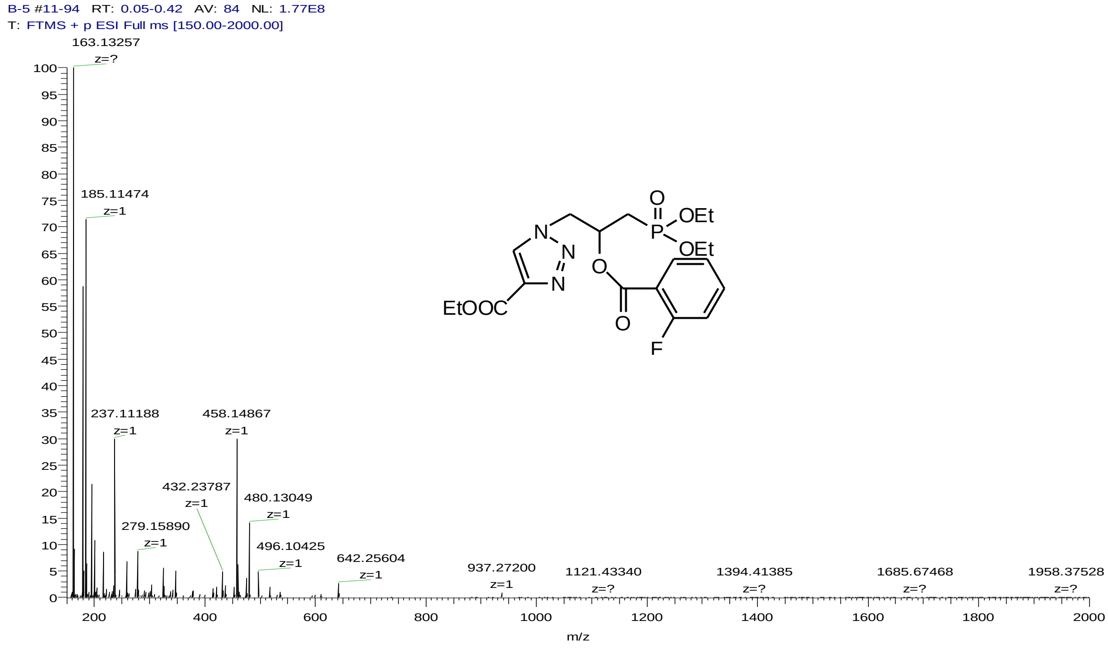

Figure S65. HRMS spectrum of compound **5-B5**.

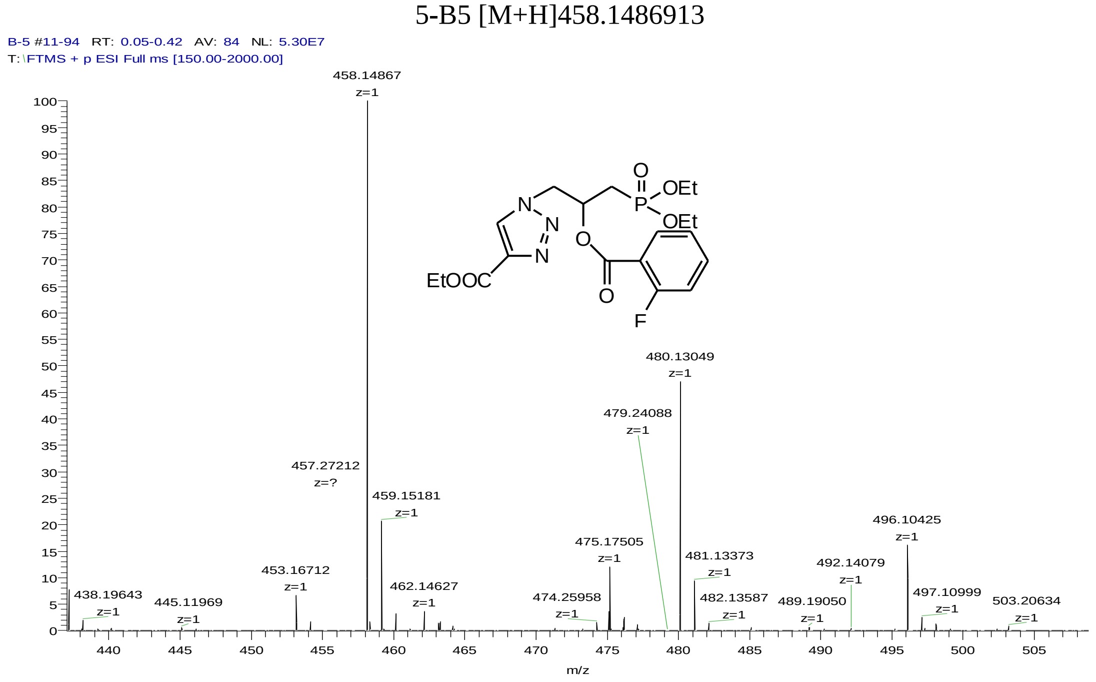

Figure S66. ^{1}H-NMR spectrum of compound **5-B6**.

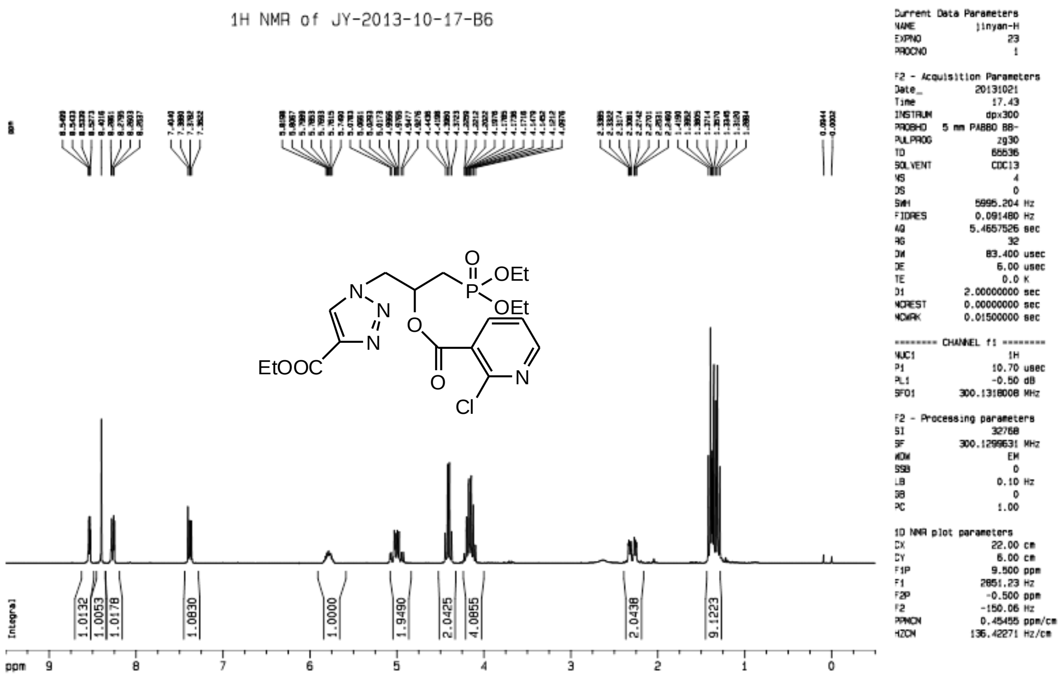

Figure S67. ^{13}C-NMR spectrum of compound **5-B6**.

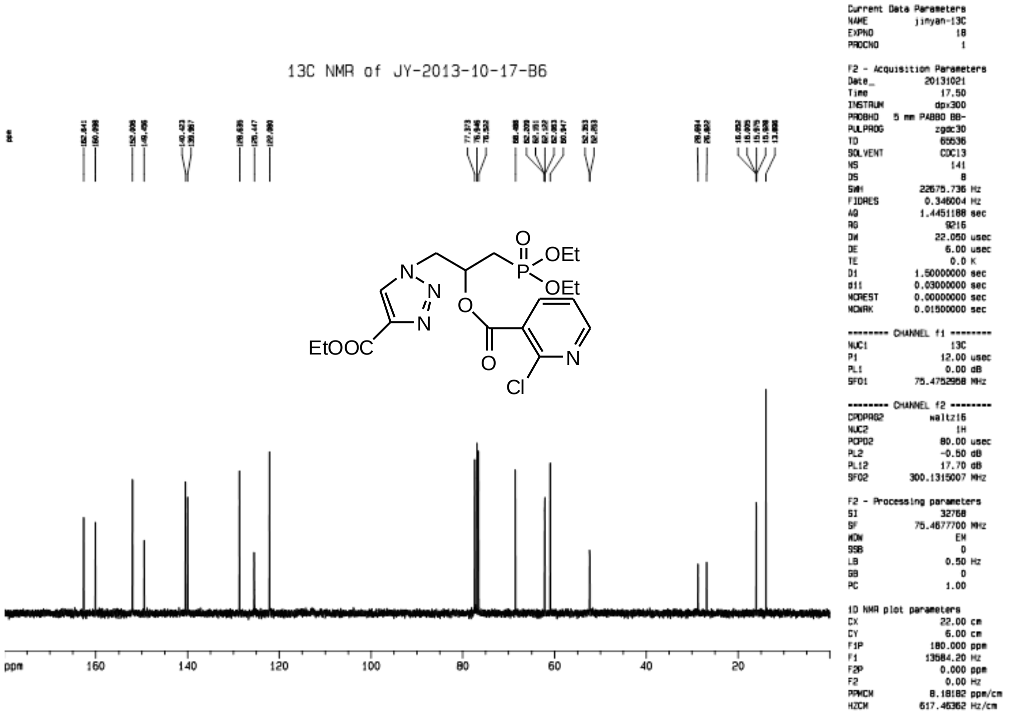

Figure S68. ^{31}P-NMR spectrum of compound **5-B6**.

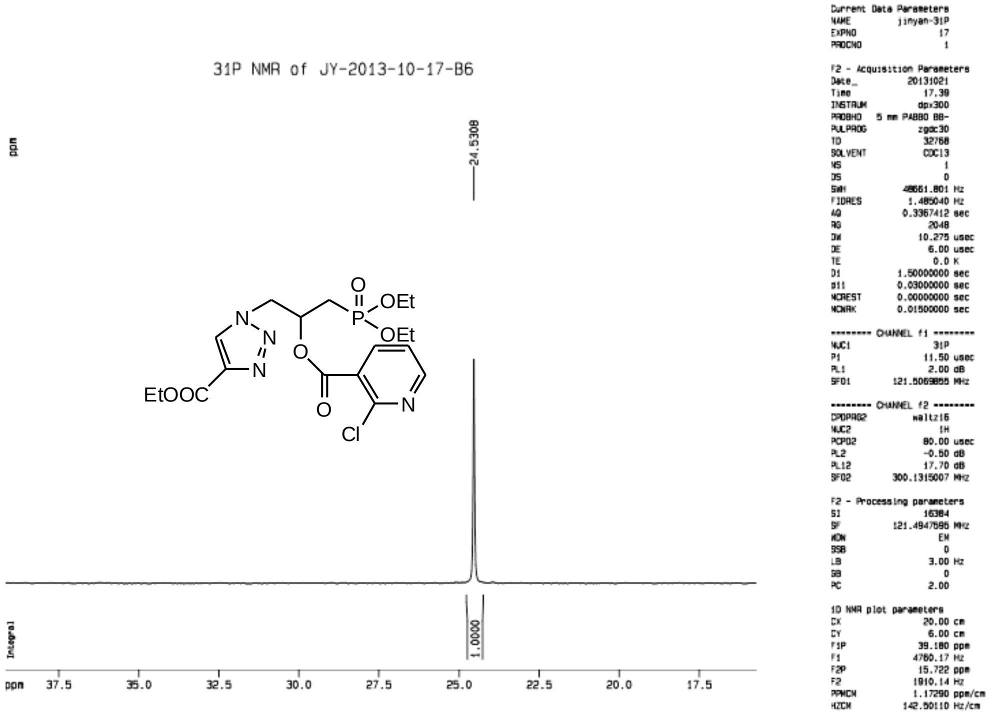

Figure S69. HRMS spectrum of compound **5-B6**.

5-B6 [M+H] 475.1143897

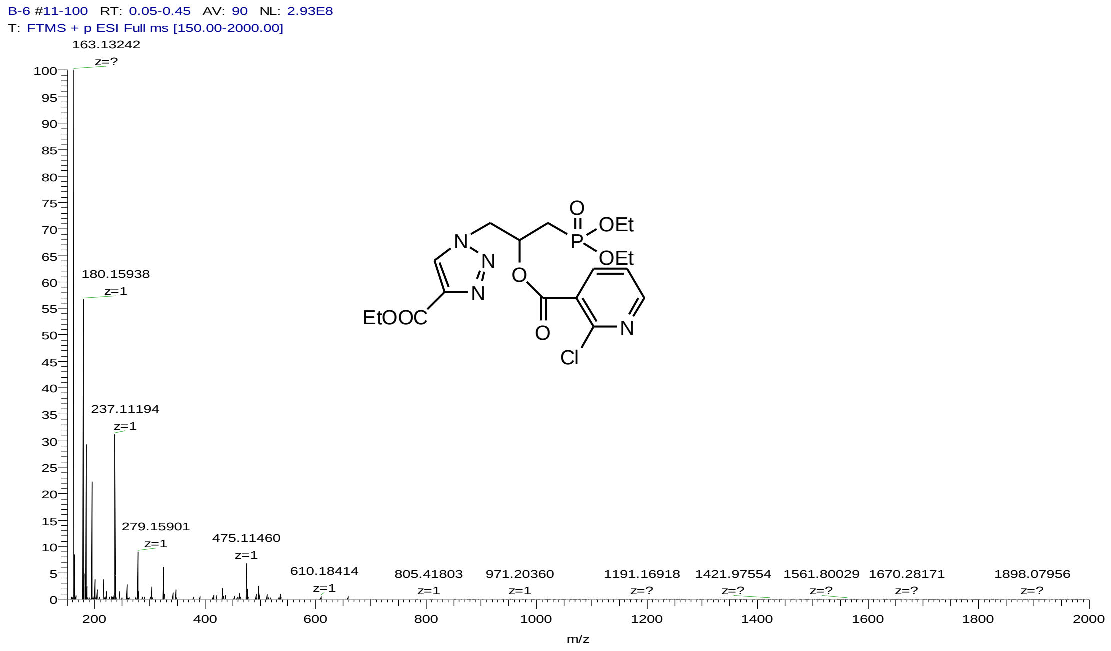

Figure S70. HRMS spectrum of compound **5-B6**.

5-B6 [M+H] 475.1143897

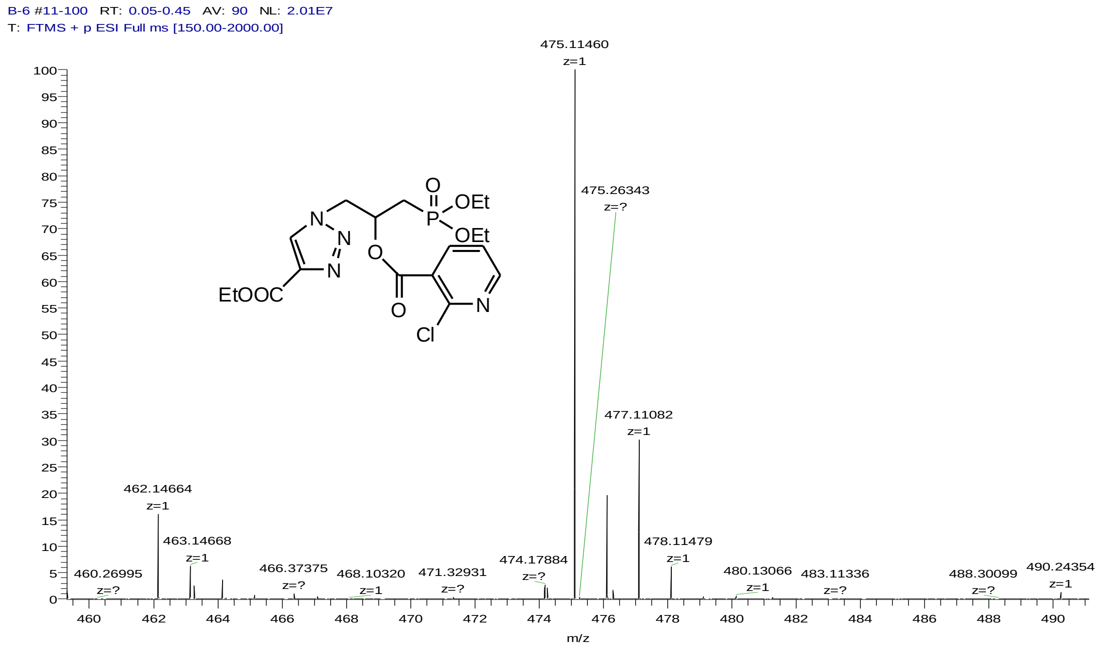

Figure S71. ^{1}H-NMR spectrum of compound **5-B7**.

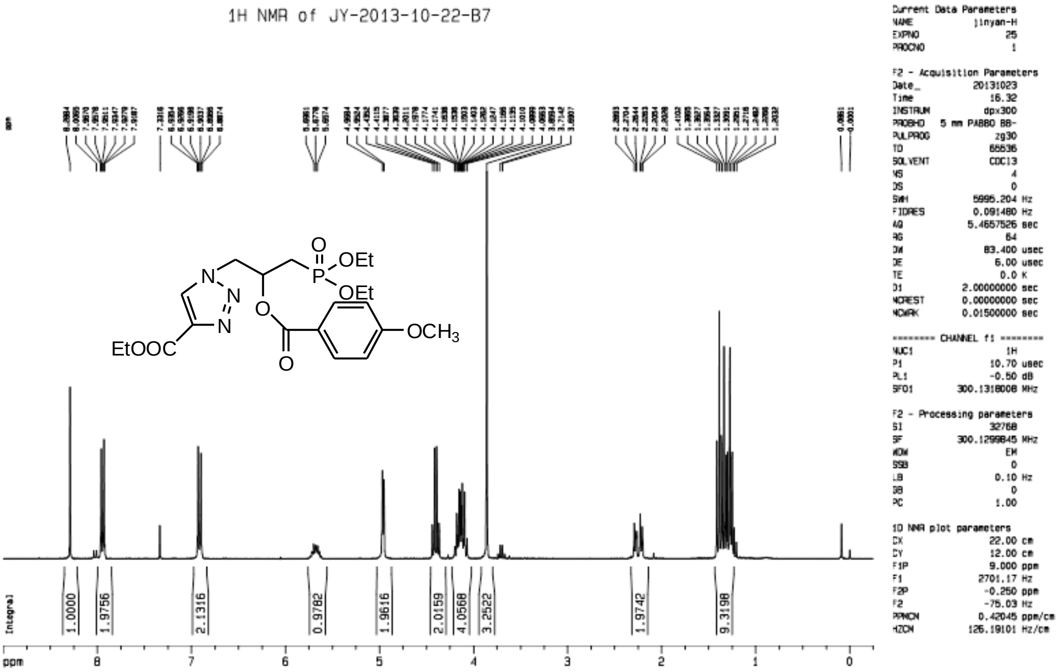

Figure S72. ^{13}C-NMR spectrum of compound **5-B7**.

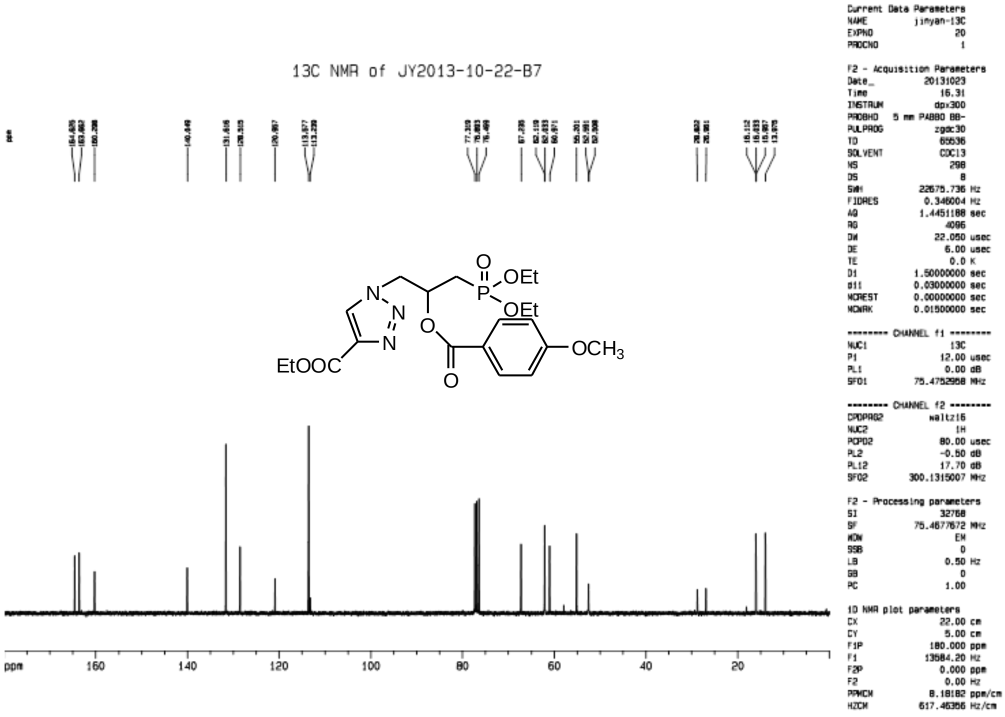

Figure S73. ^{31}P-NMR spectrum of compound **5-B7**.

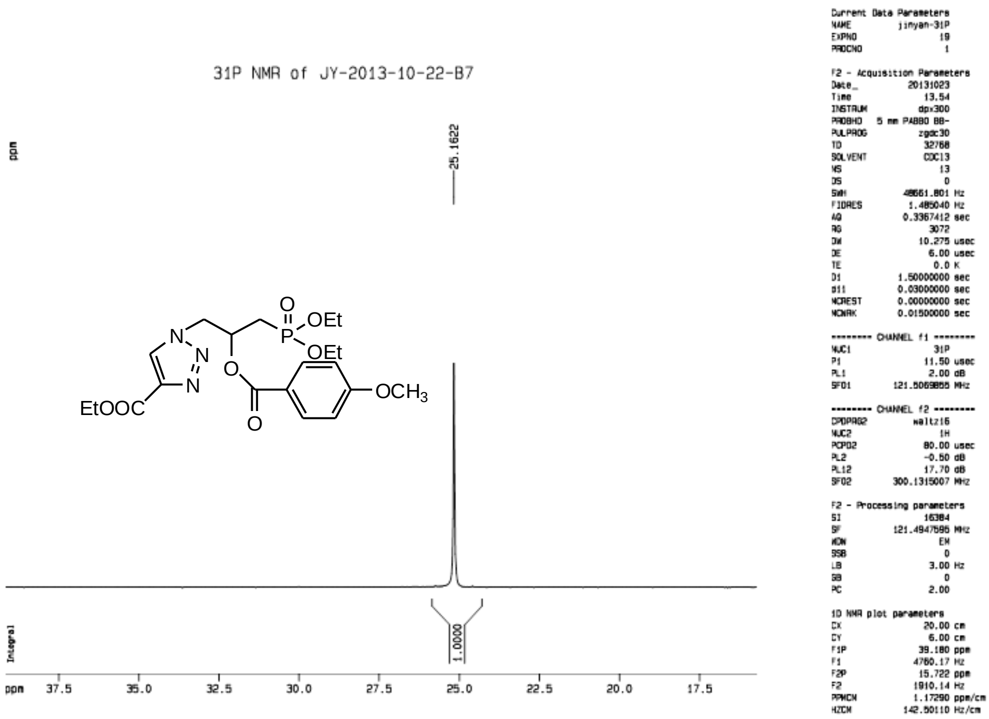

Figure S74. HRMS spectrum of compound **5-B7**.

5-B7 [M+H] 470.1686778

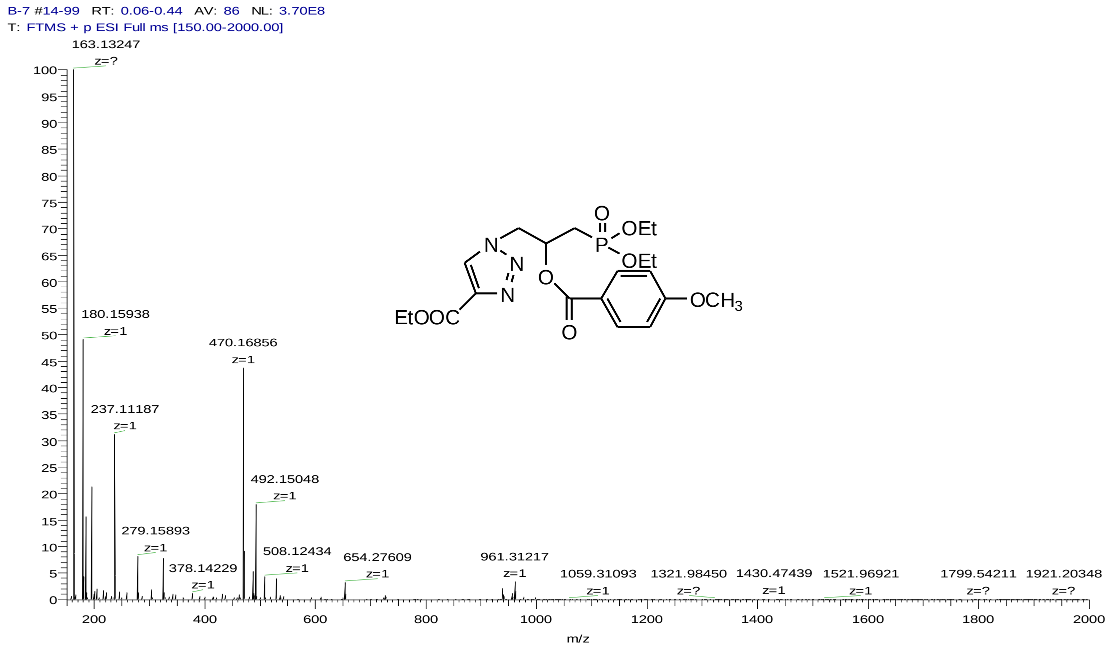

Figure S75. HRMS spectrum of compound **5-B7**.

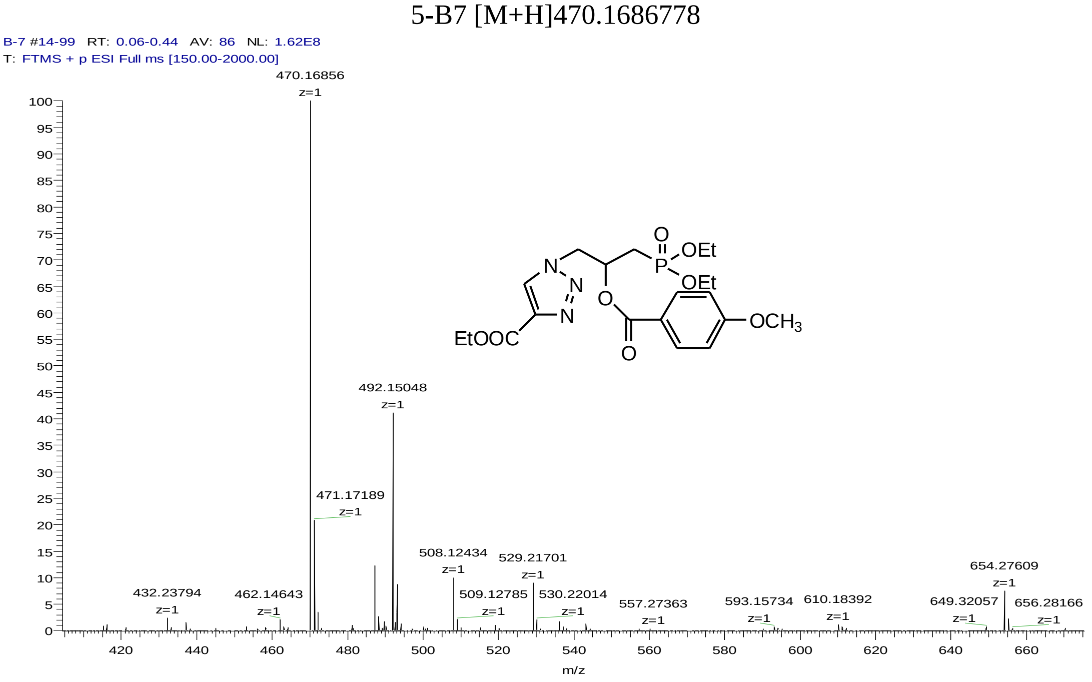

Figure S76. ^{1}H-NMR spectrum of compound **5-B8**.

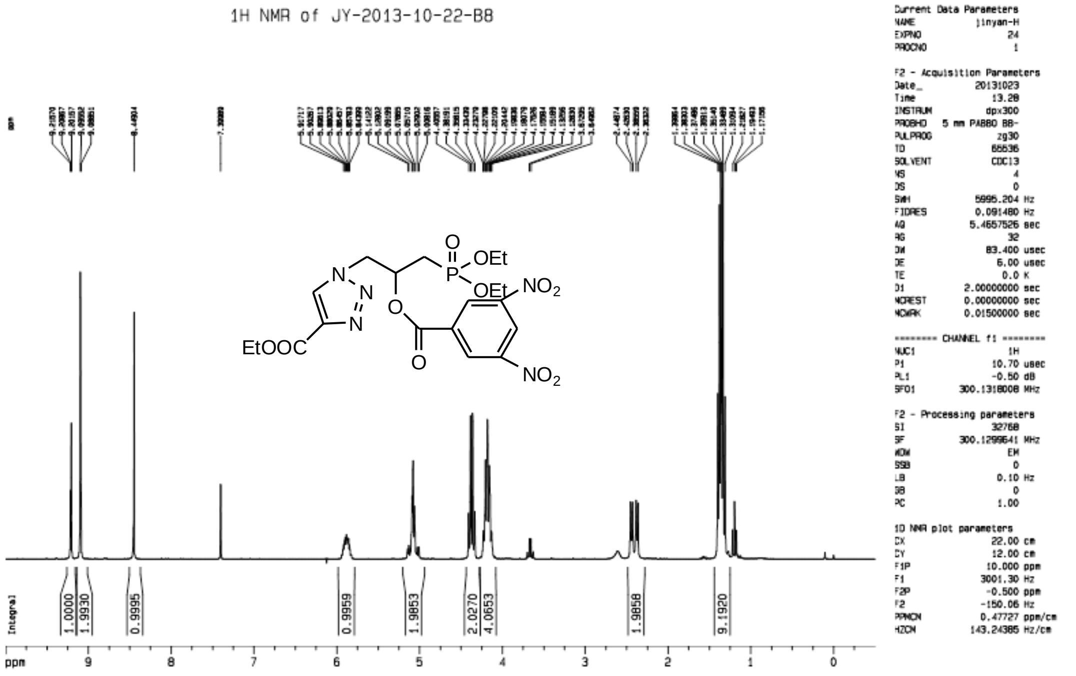

Figure S77. ^{13}C-NMR spectrum of compound **5-B8**.

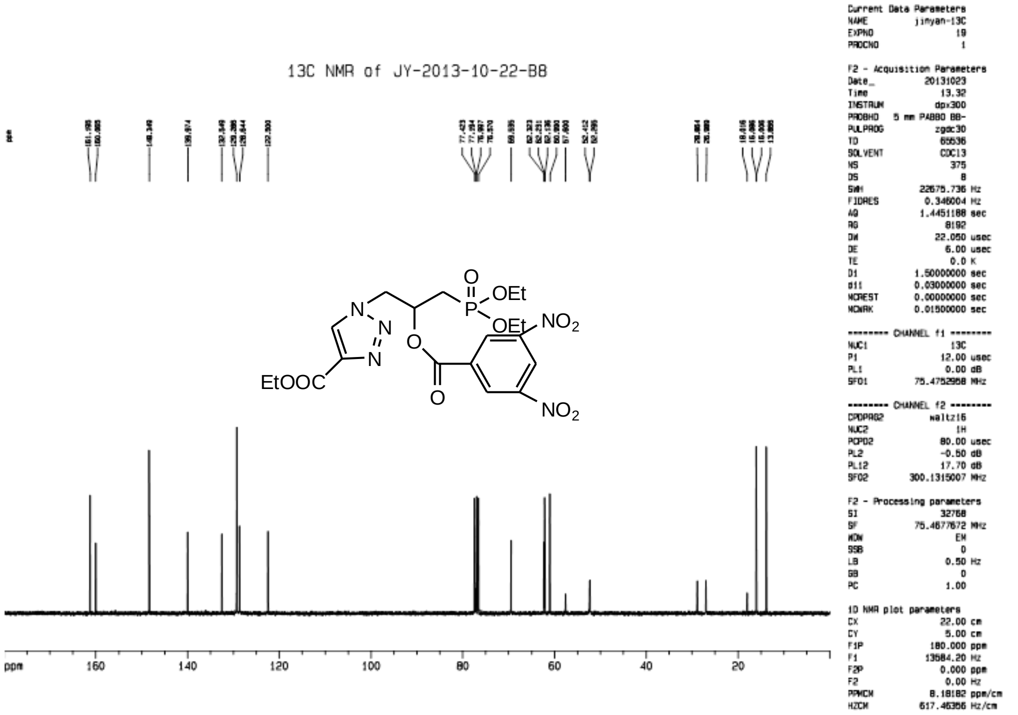

Figure S78. ^{31}P-NMR spectrum of compound **5-B8**.

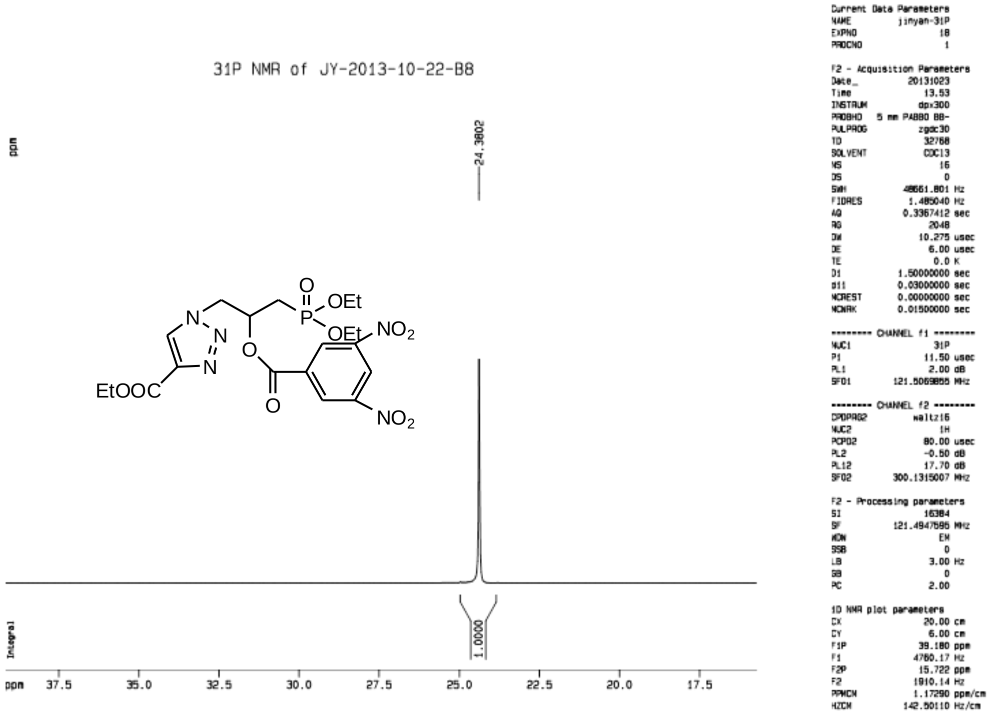

Figure S79. HRMS spectrum of compound **5-B8**.

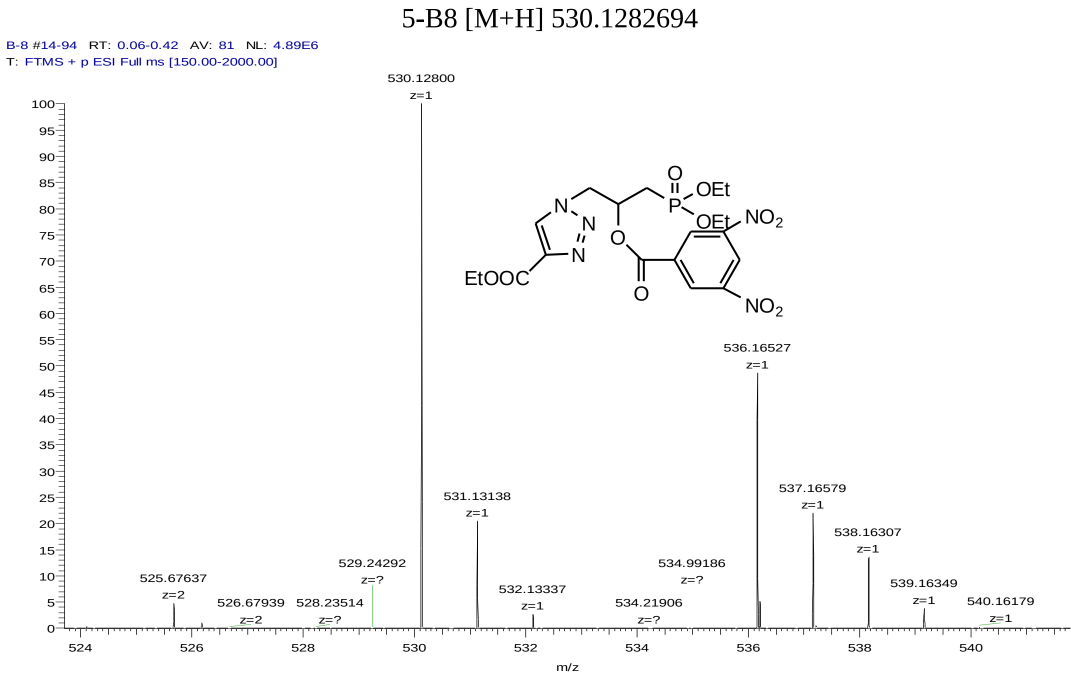

Figure S80. ^{1}H-NMR spectrum of compound **5-C1**.

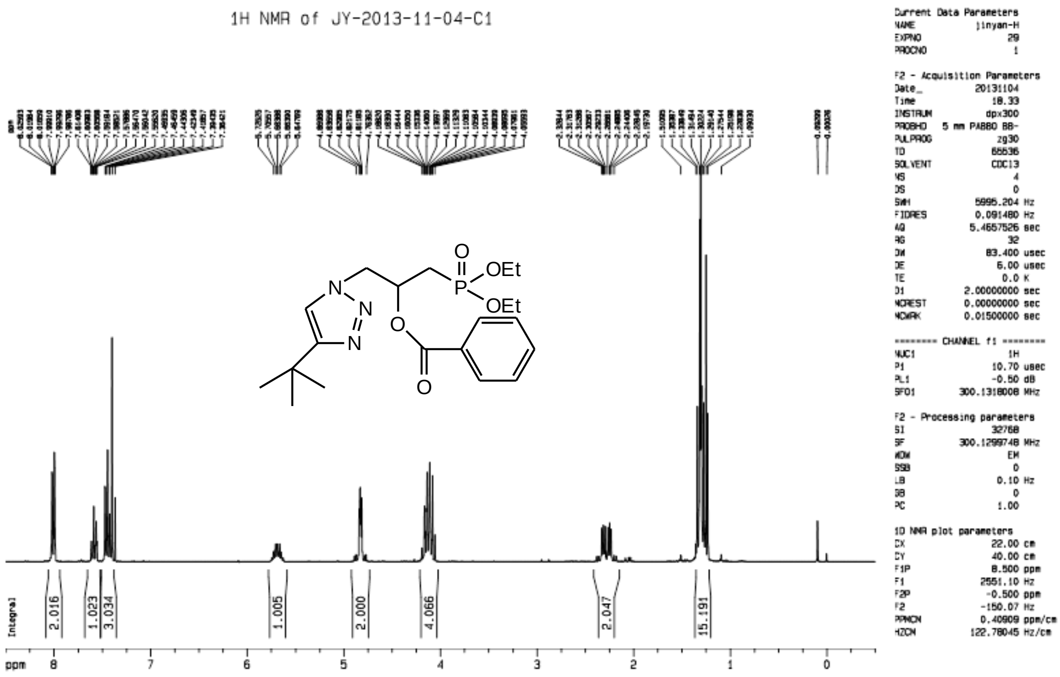

Figure S81. ^{13}C-NMR spectrum of compound **5-C1**.

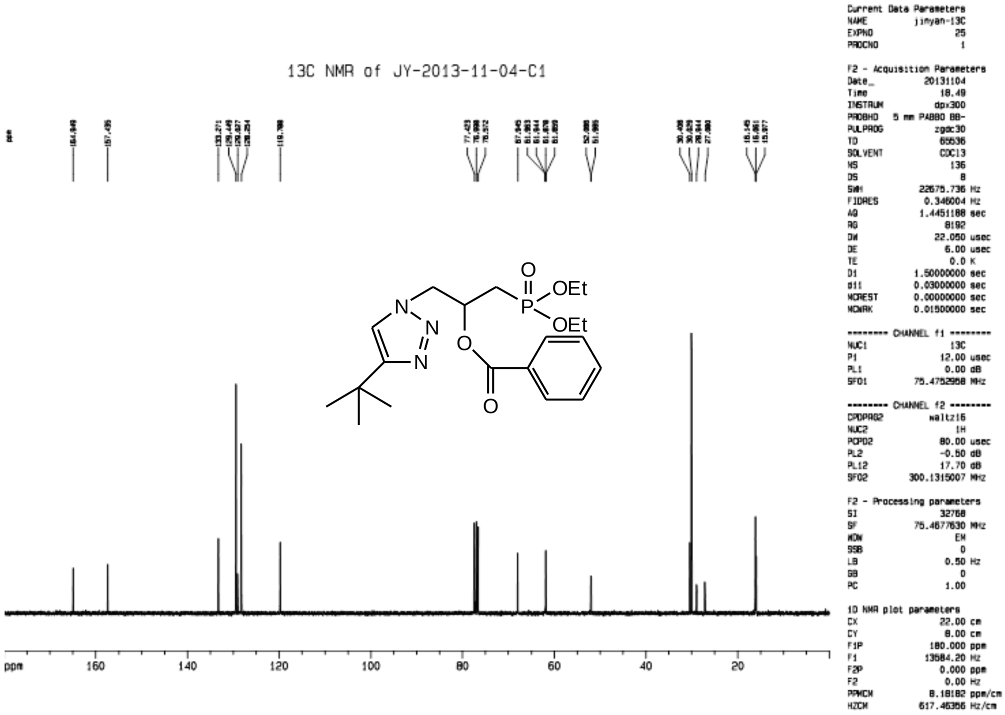

Figure S82. ^{31}P-NMR spectrum of compound **5-C1**.

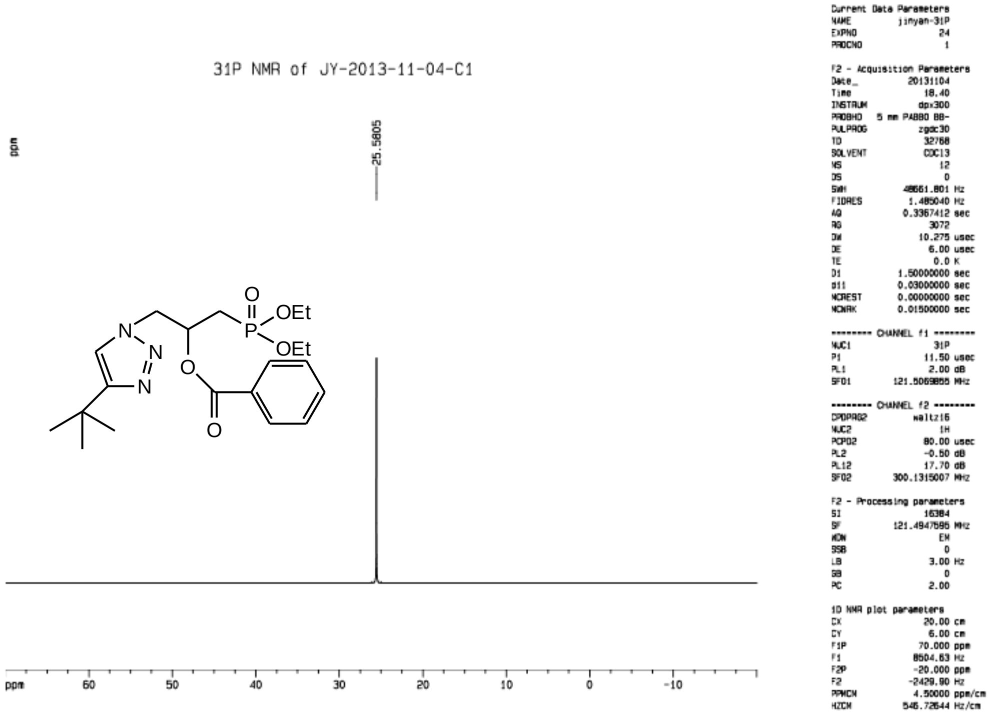

Figure S83. HRMS spectrum of compound **5-C1**.

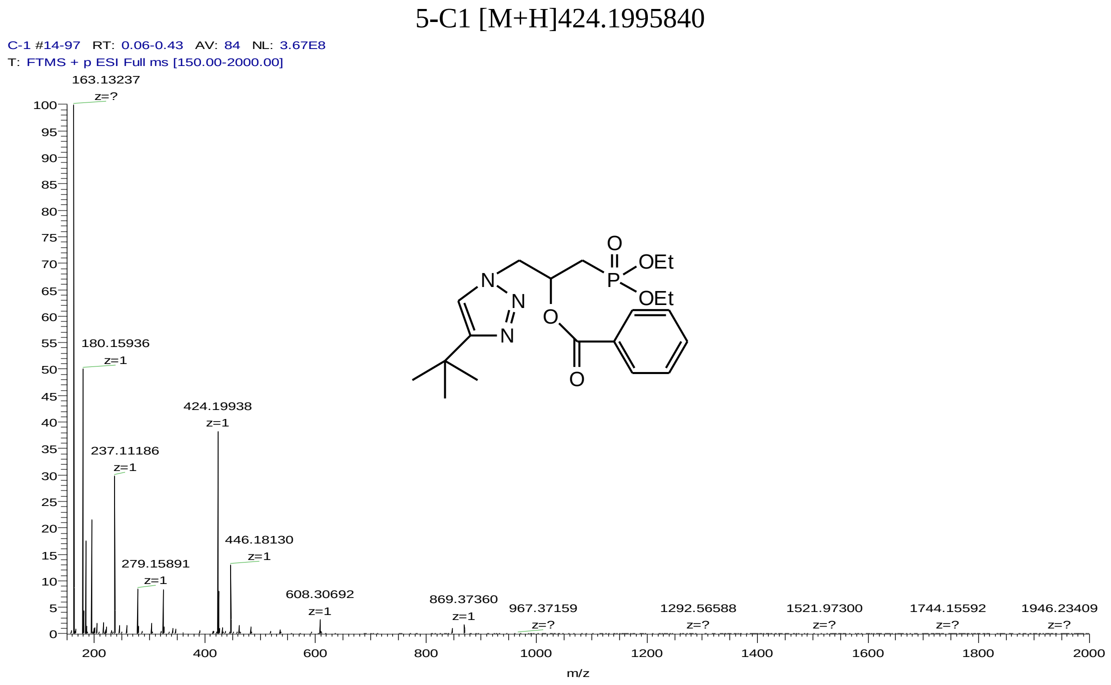

Figure S84. HRMS spectrum of compound **5-C1**.

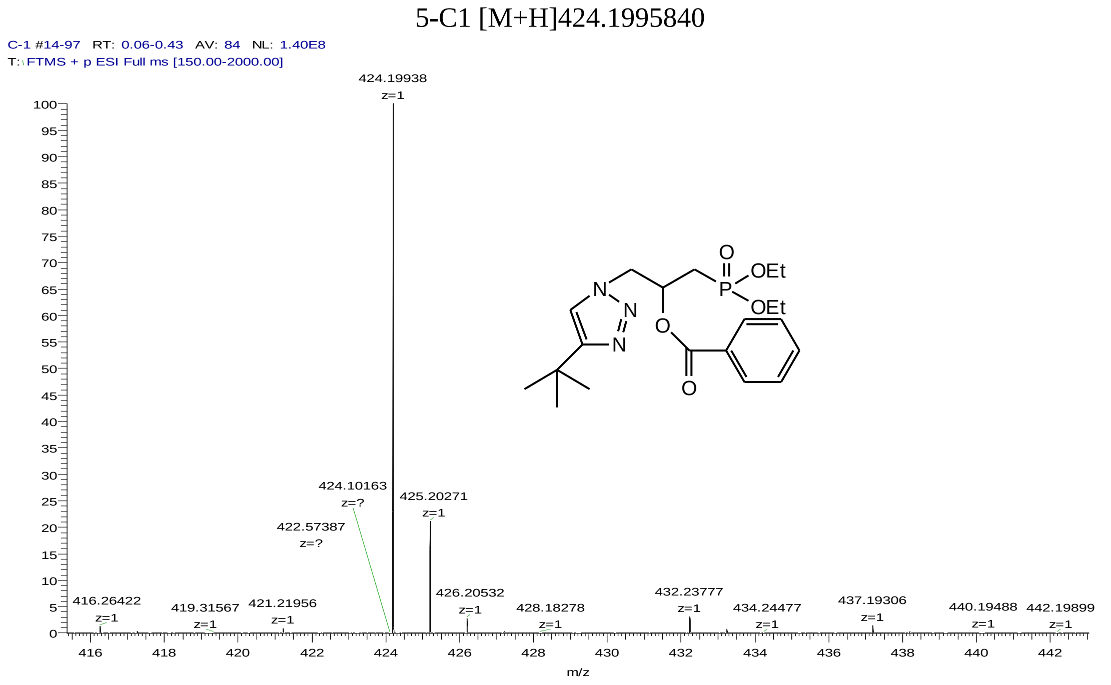

Figure S85. ^{1}H-NMR spectrum of compound **5-C2**.

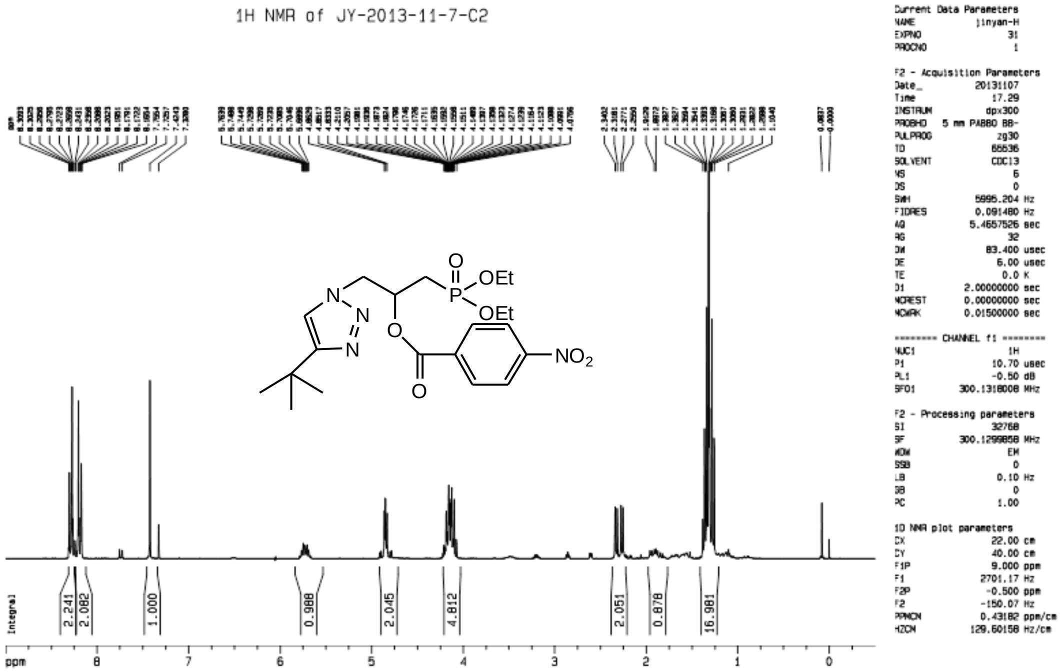

Figure S86. ^{13}C-NMR spectrum of compound **5-C2**.

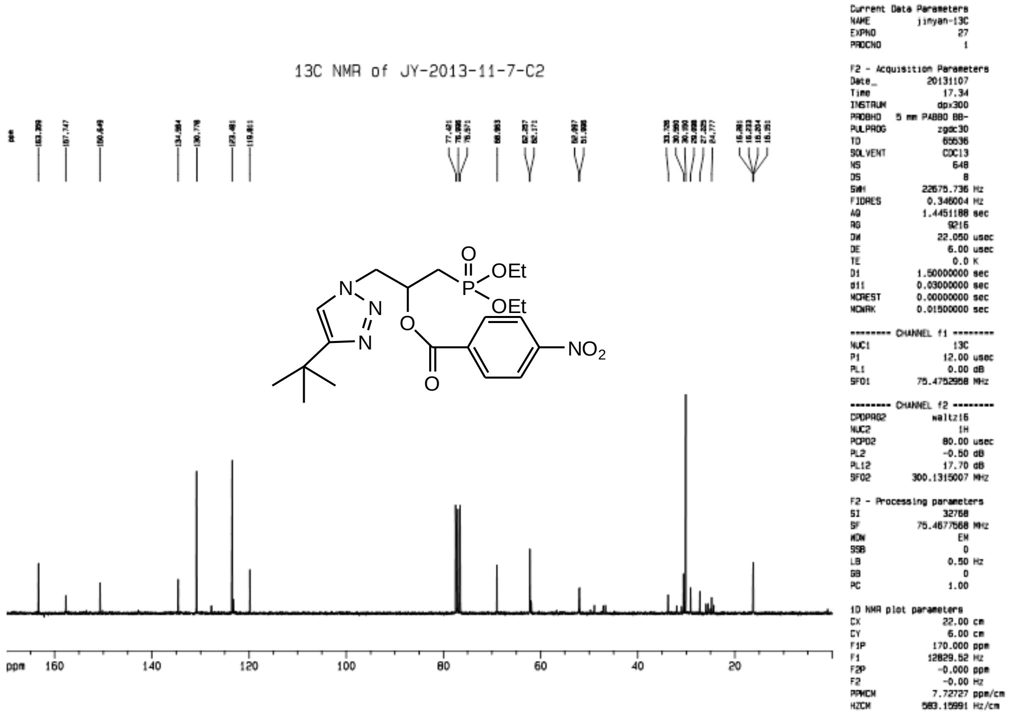

Figure S87. ^{31}P-NMR spectrum of compound **5-C2**.

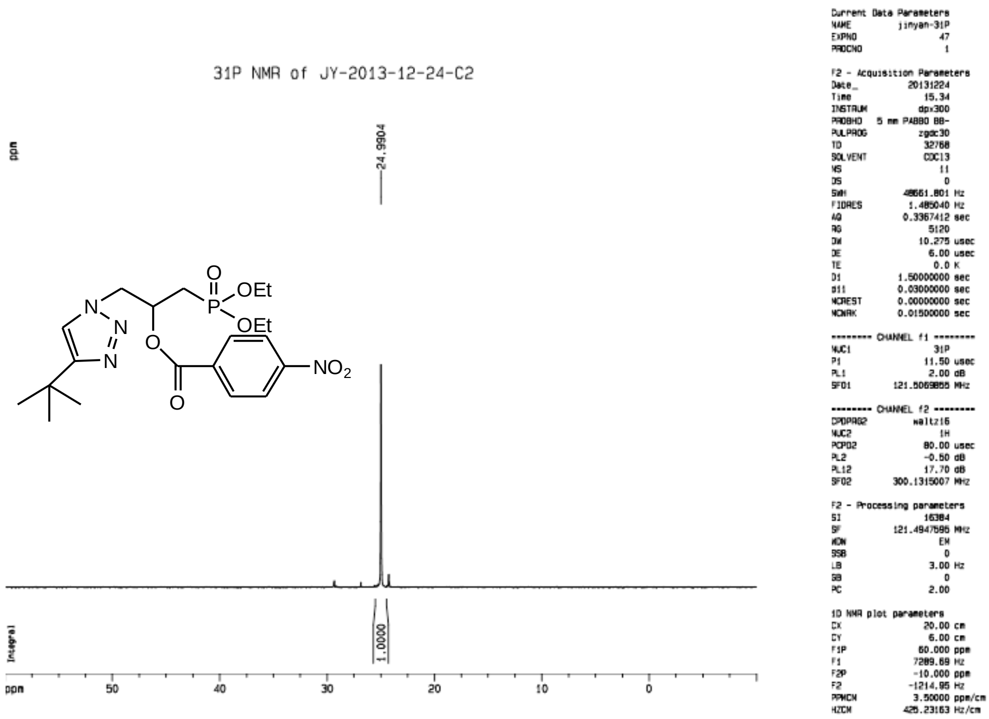

Figure S88. HRMS spectrum of compound **5-C2**.

5-C2 [M+H] 469.1846622

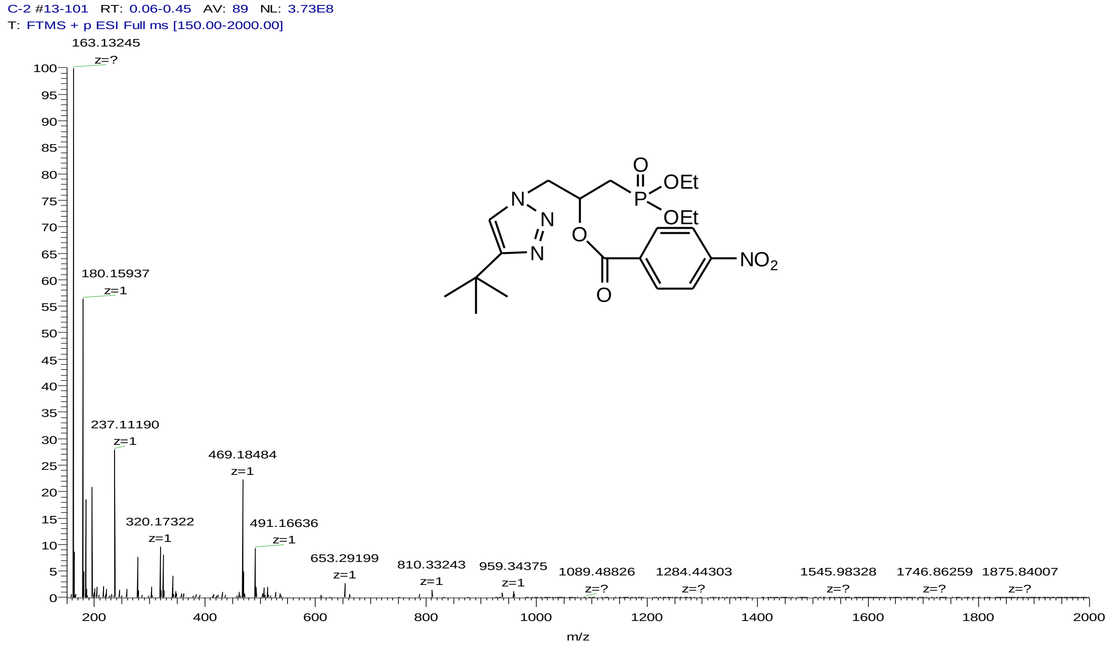

Figure S89. HRMS spectrum of compound **5-C2**.

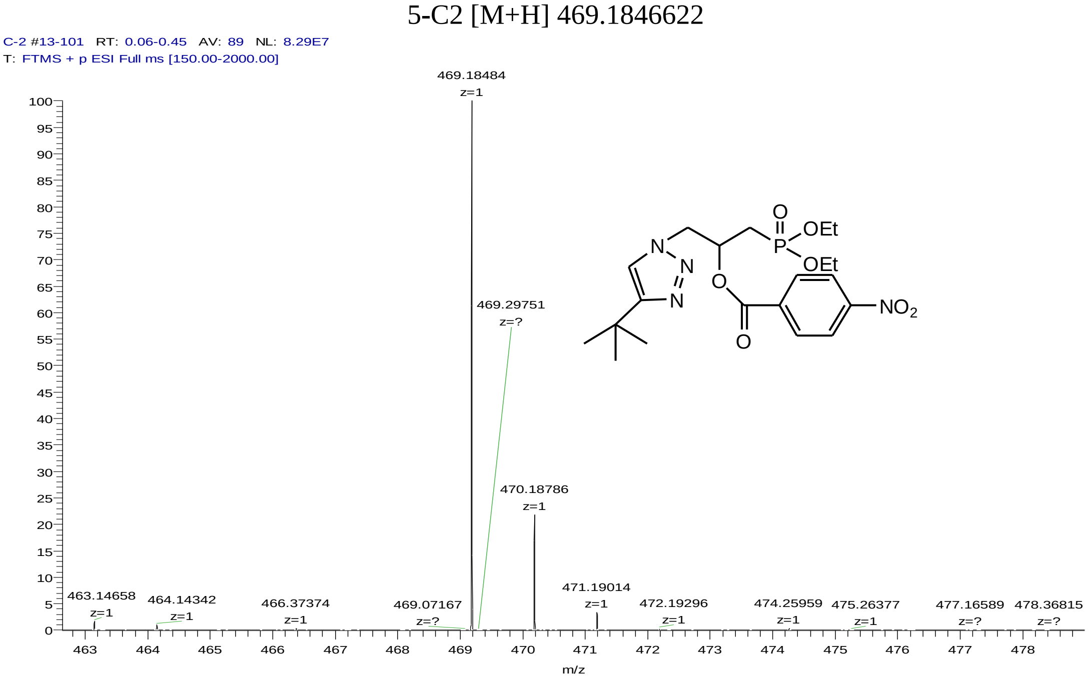

Figure S90. ^{1}H-NMR spectrum of compound **5-C3**.

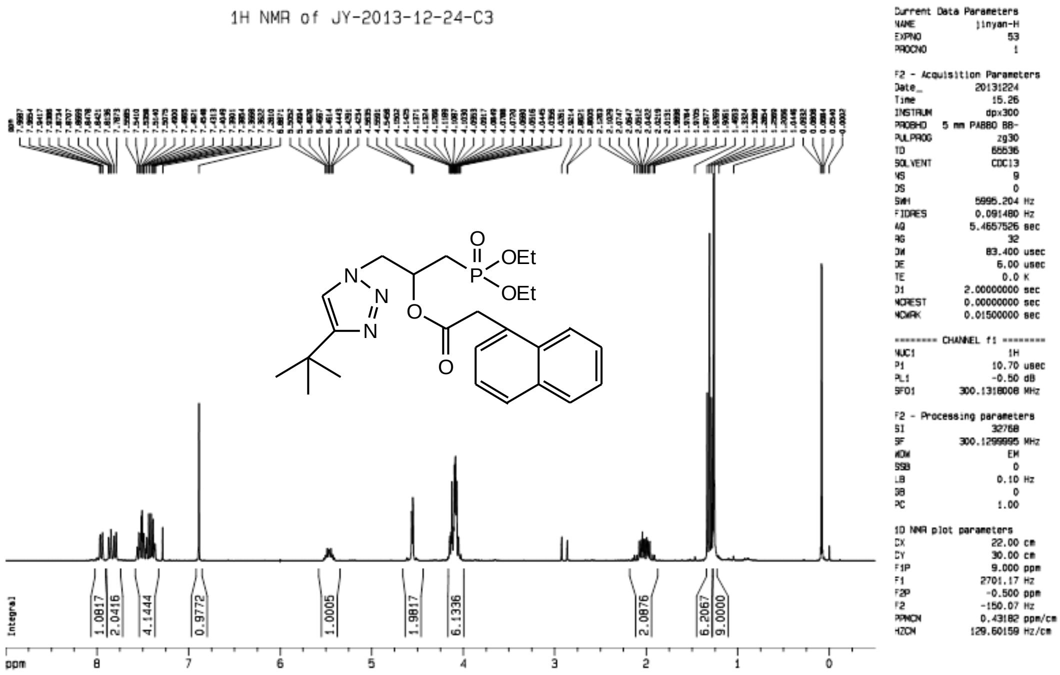

Figure S91. ^{13}C-NMR spectrum of compound **5-C3**.

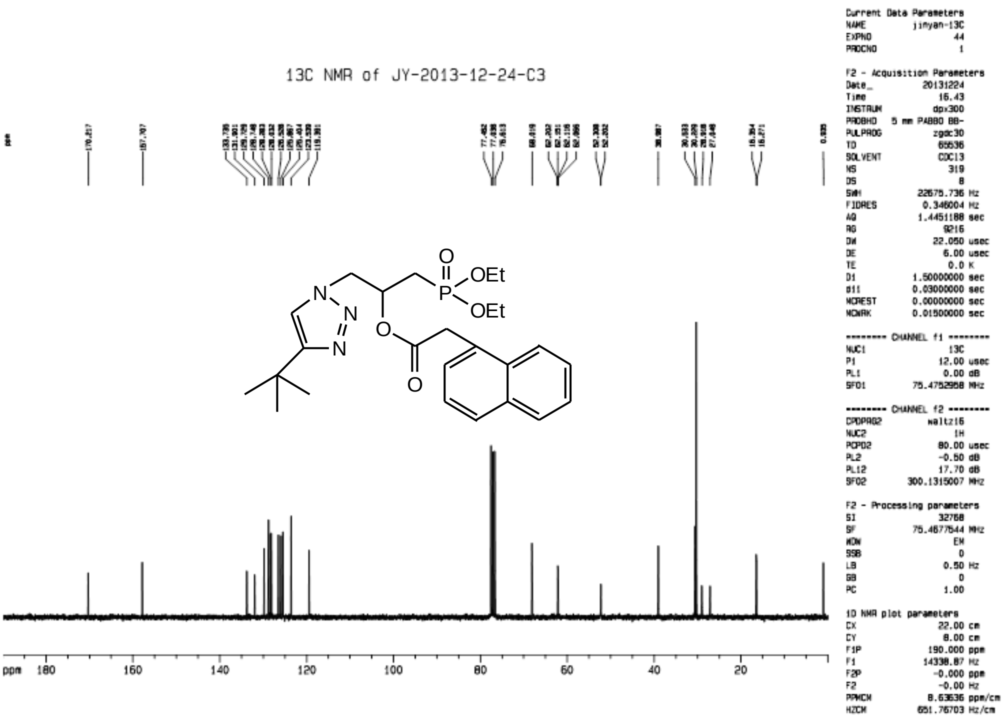

Figure S92. ^{31}P-NMR spectrum of compound **5-C3**.

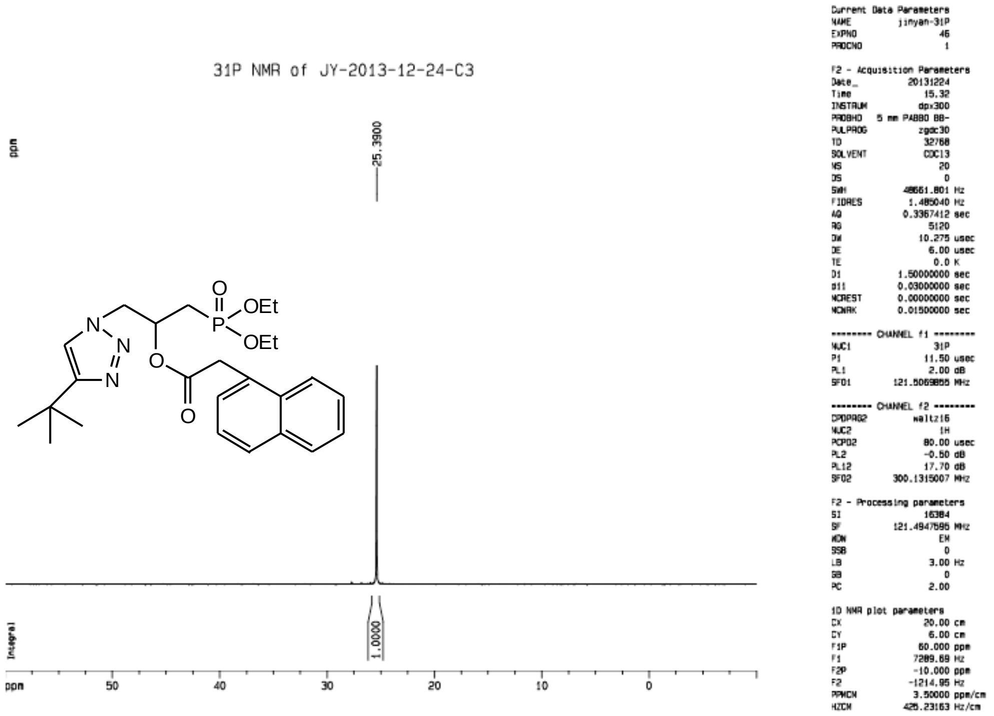

Figure S93. HRMS spectrum of compound **5-C3**.

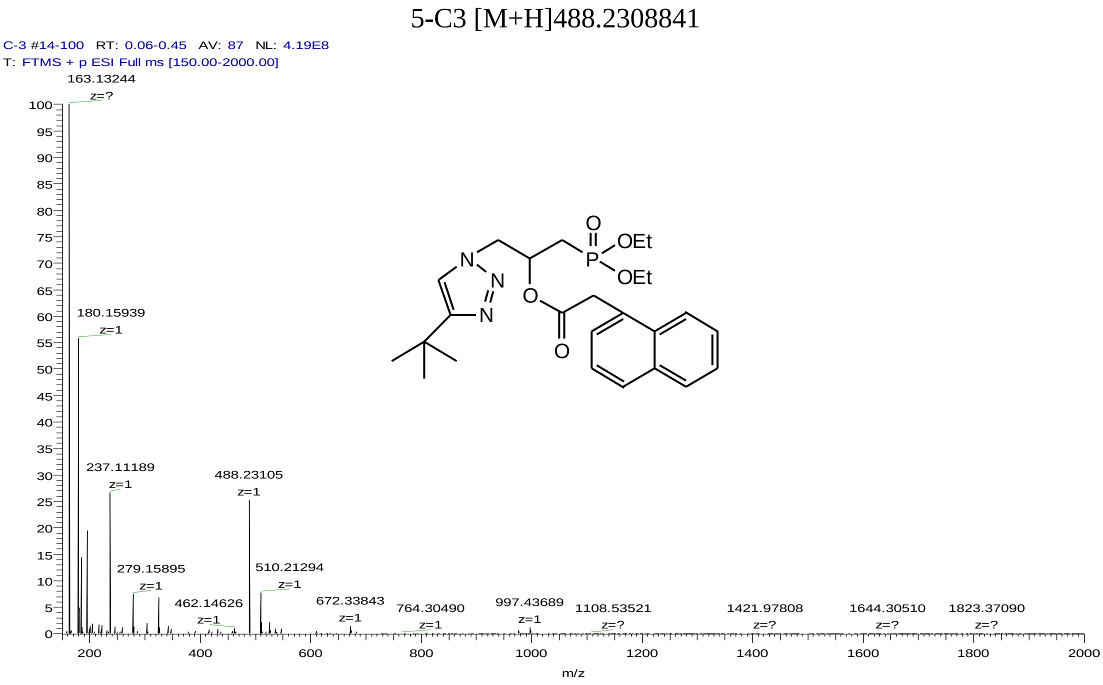

Figure S94. HRMS spectrum of compound **5-C3**.

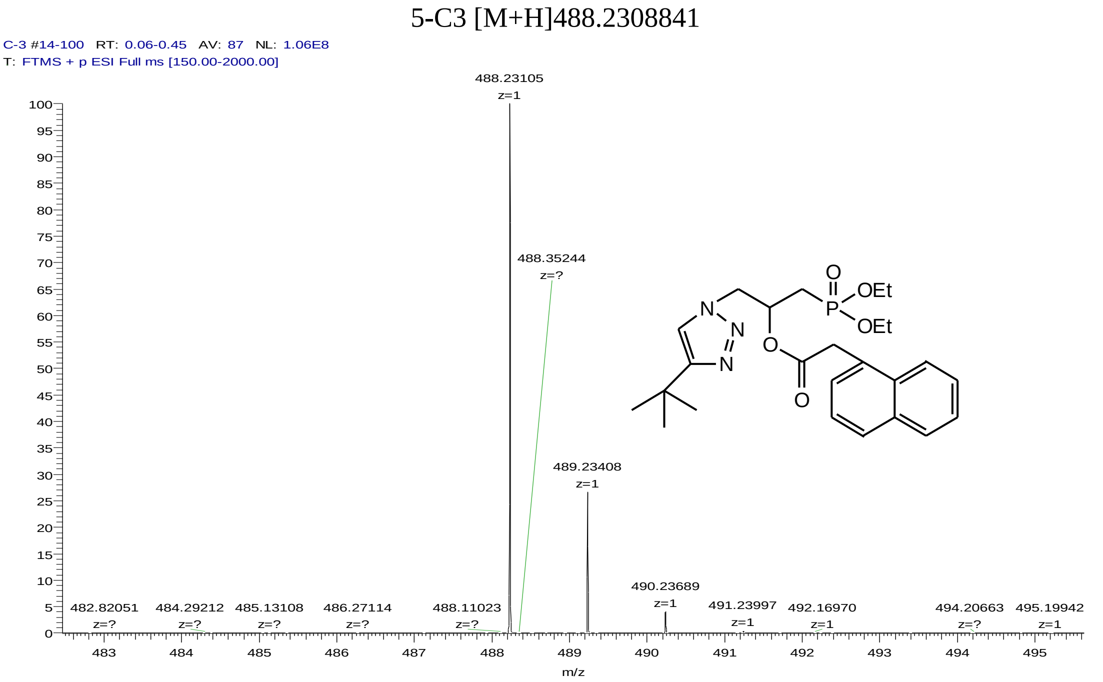

Figure S95. ^{1}H-NMR spectrum of compound **5-C4**.

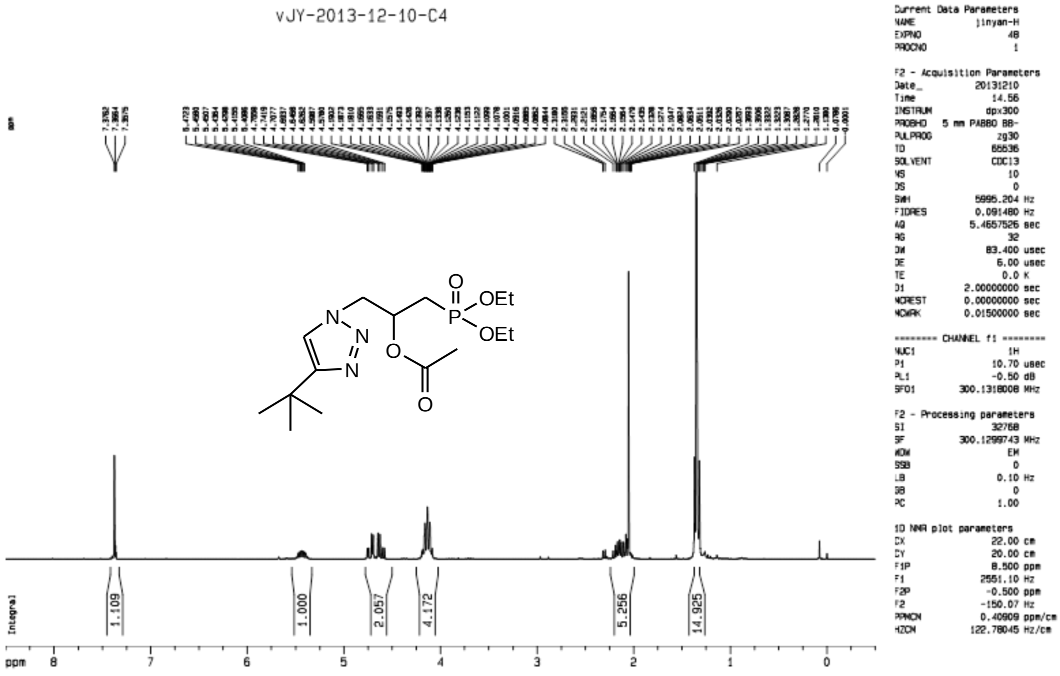

Figure S96. ^{13}C-NMR spectrum of compound **5-C4**.

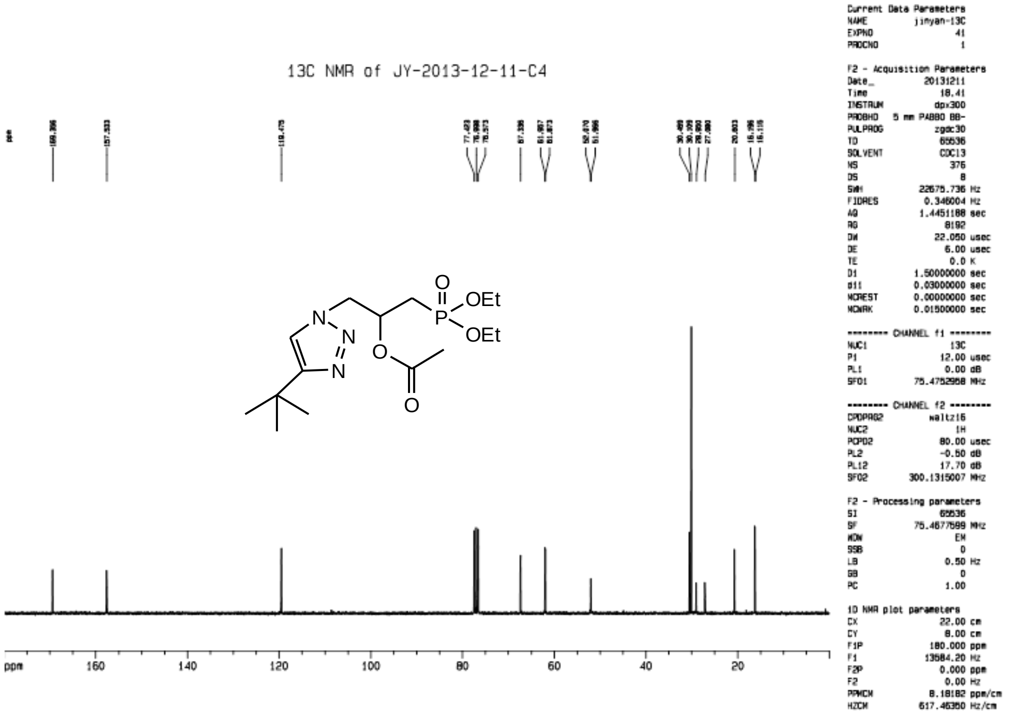

Figure S97. ^{31}P-NMR spectrum of compound **5-C4.**

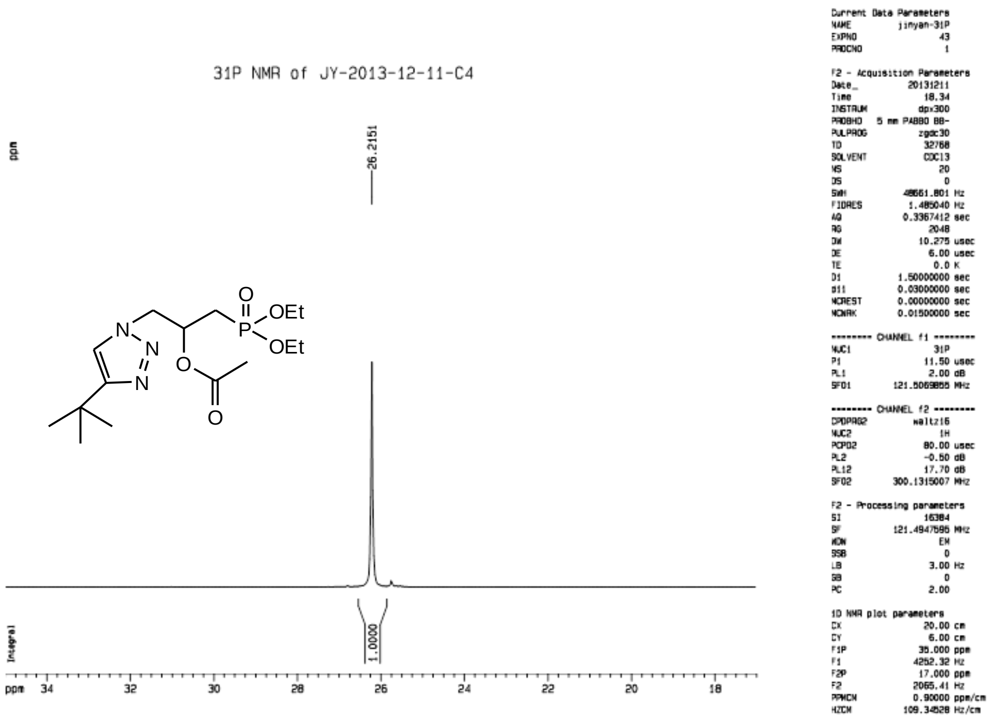

Figure S98. HRMS spectrum of compound **5-C4**.

5-C4 [M+H] 362.1839340

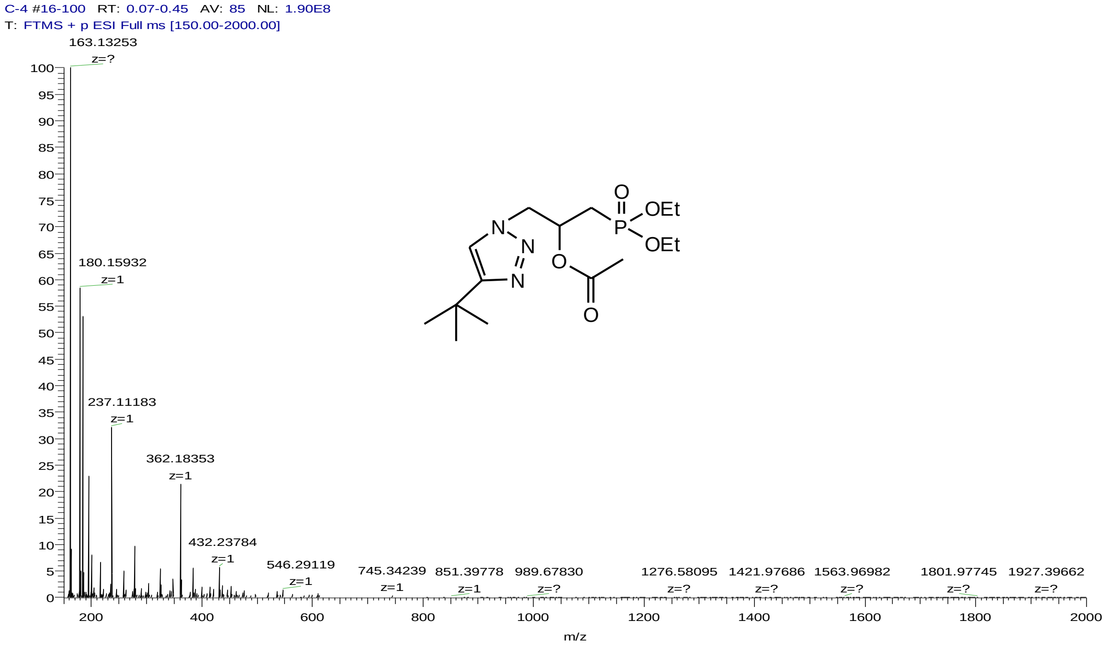

Figure S99. HRMS spectrum of compound **5-C4**.

5-C4 [M+H] 362.1839340

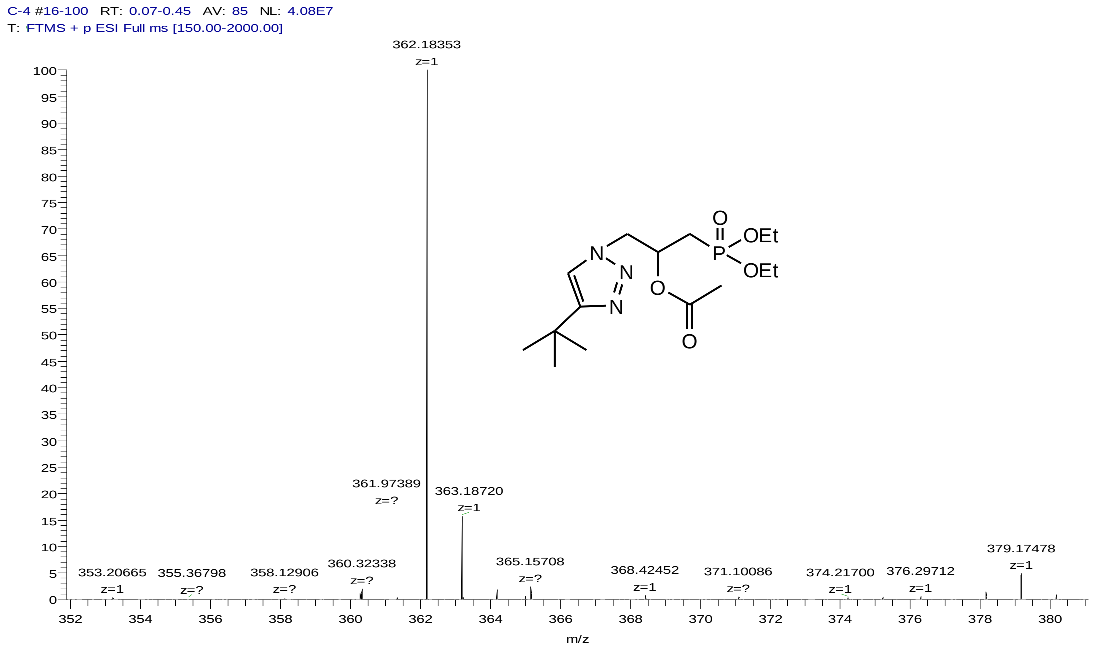

Figure S100. ^{1}H-NMR spectrum of compound **5-C5**.

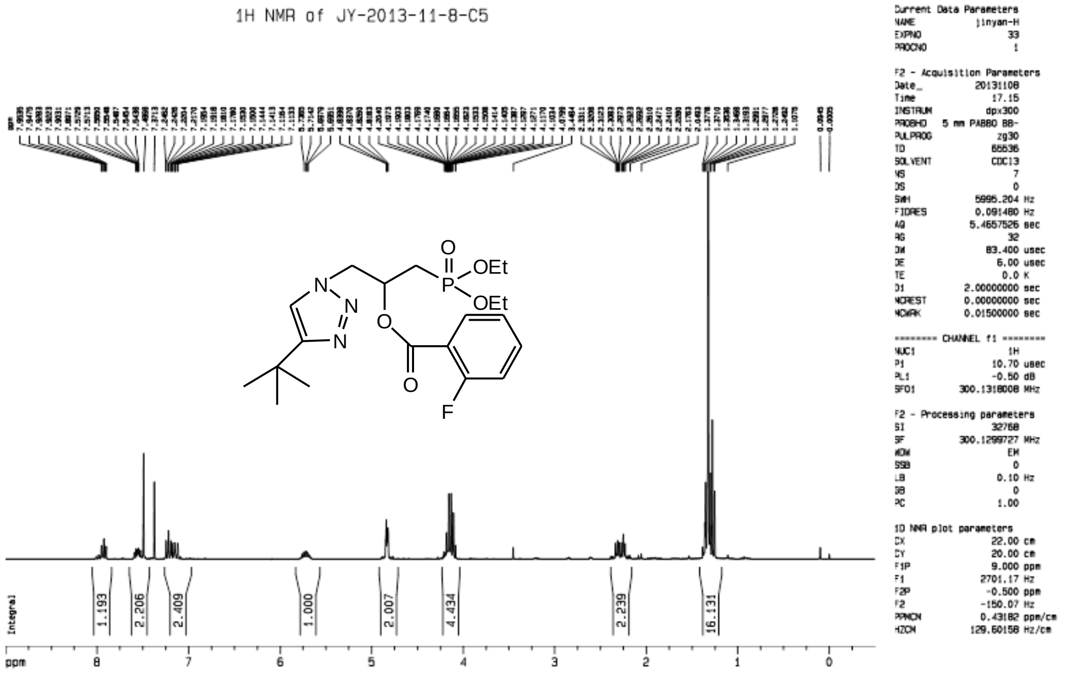

Figure S101. ^{13}C-NMR spectrum of compound **5-C5**.

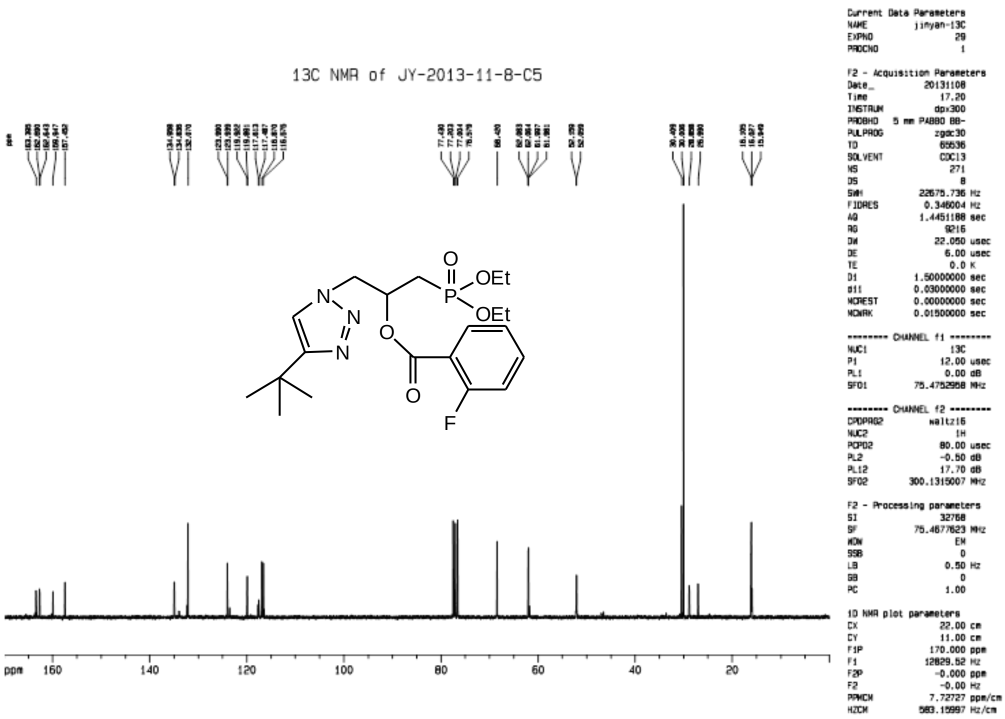

Figure S102. ^{31}P-NMR spectrum of compound **5-C5**.

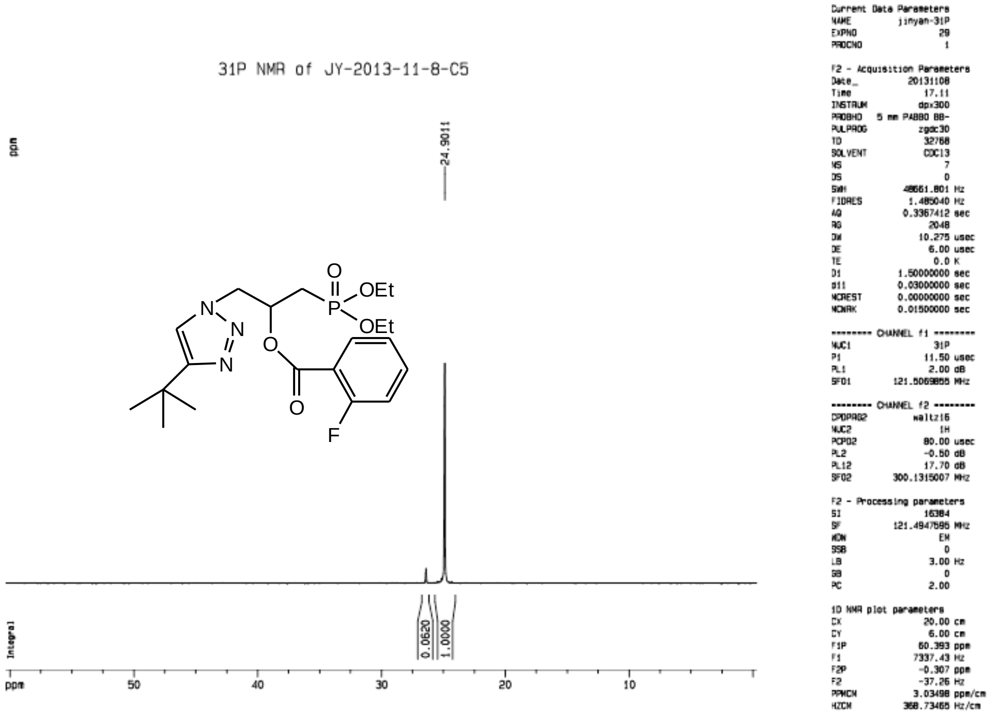

Figure S103. HRMS spectrum of compound **5-C5**.

5-C5 [M+H] 442.1901622

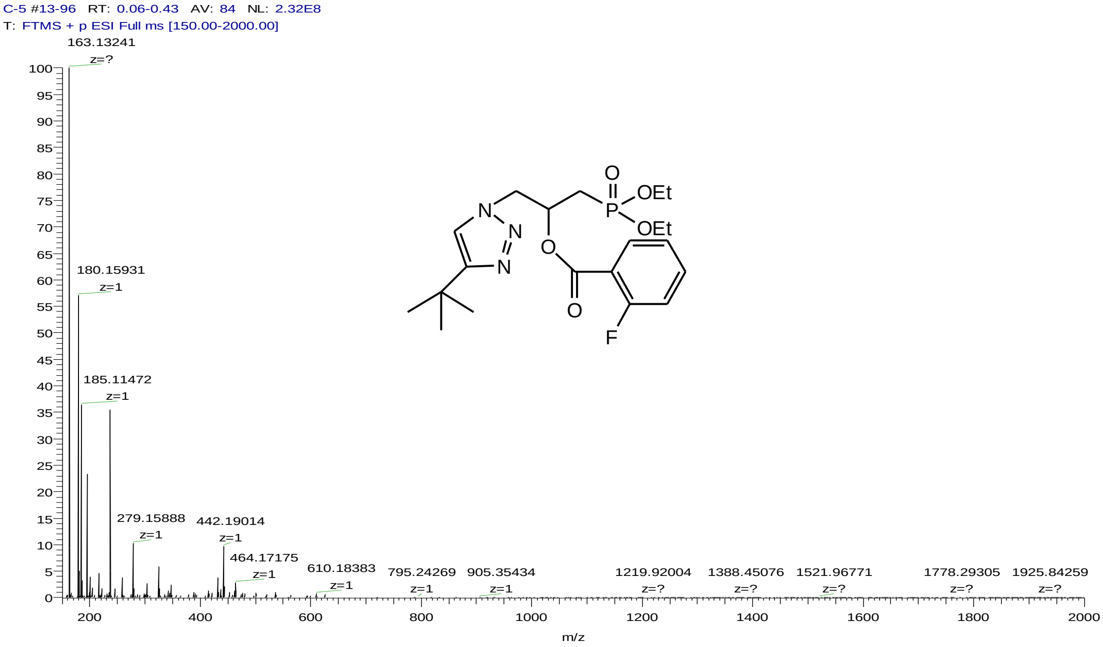

Figure S104. HRMS spectrum of compound **5-C5**.

5-C5 [M+H] 442.1901622

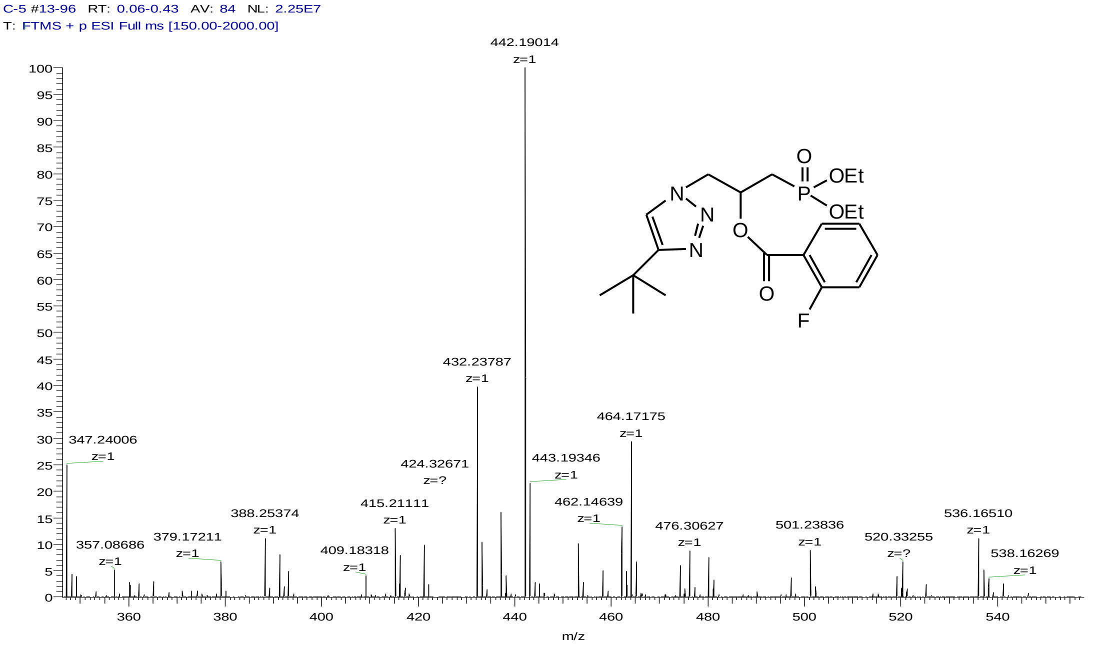

Figure S105. ^{1}H-NMR spectrum of compound **5-C6**.

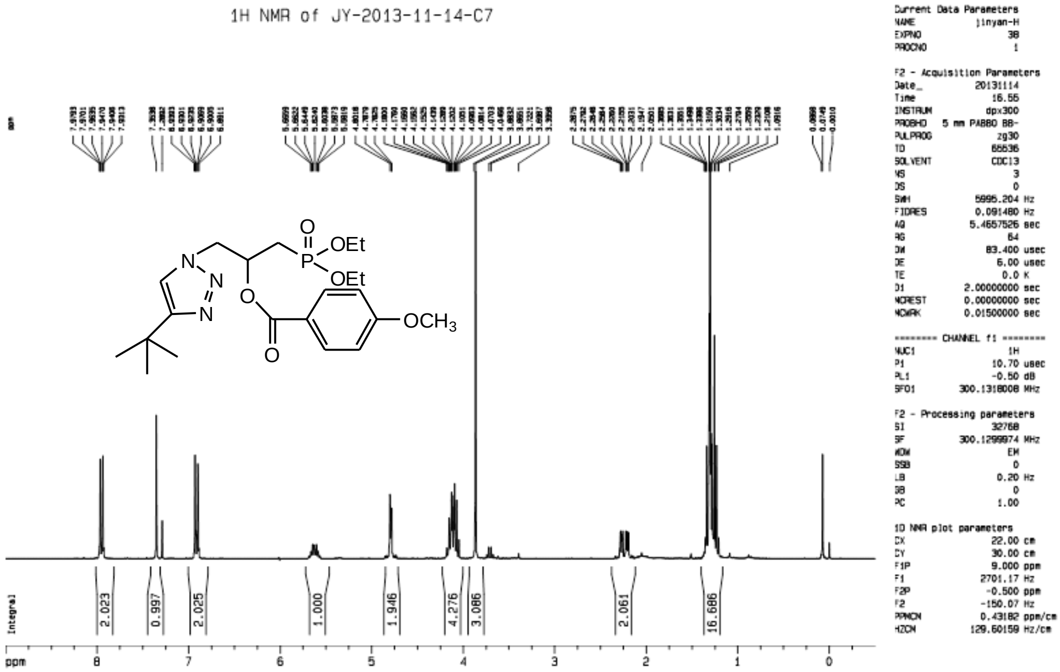

Figure S106. ^{13}C-NMR spectrum of compound **5-C6.**

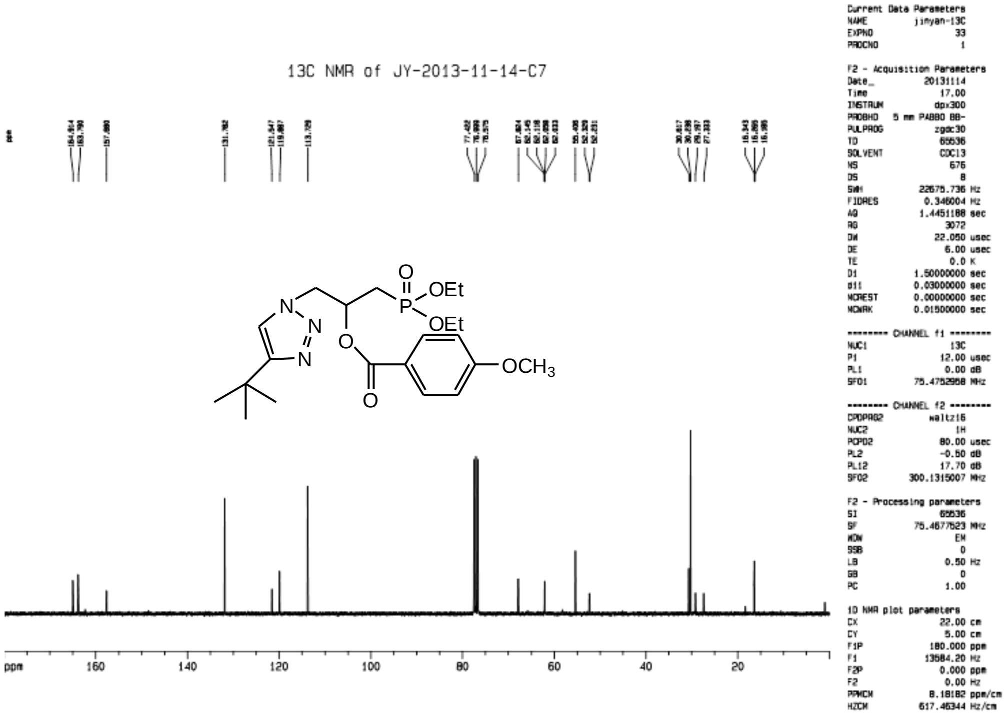

Figure S107. ^{31}P-NMR spectrum of compound **5-C6**.

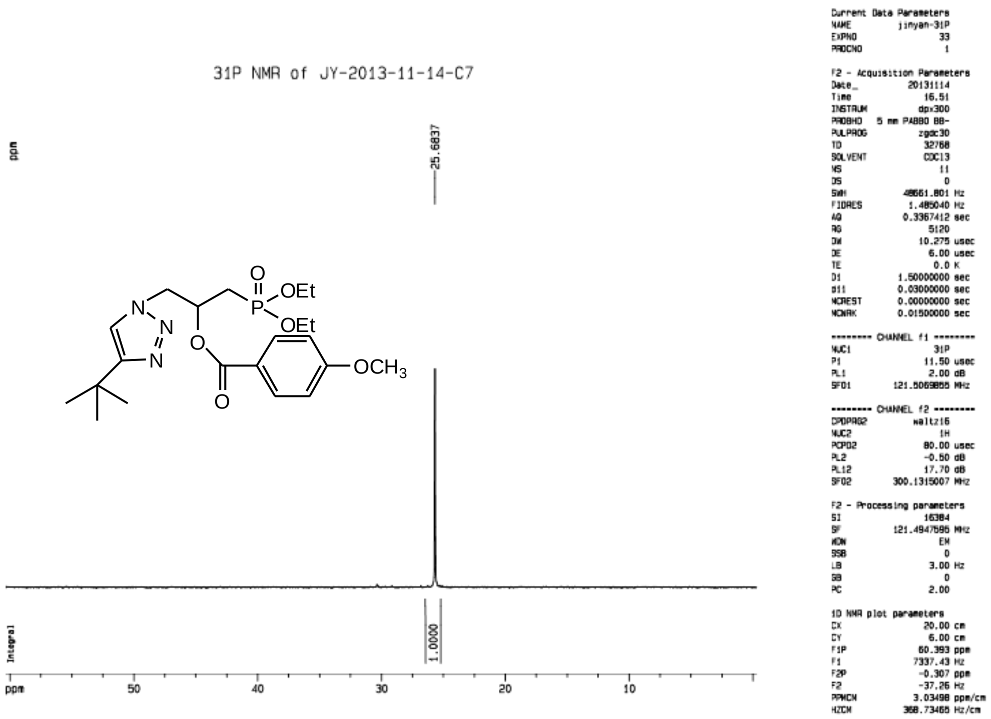

Figure S108. HRMS spectrum of compound **5-C6**.

5-C6 [M+H] 454.2101487

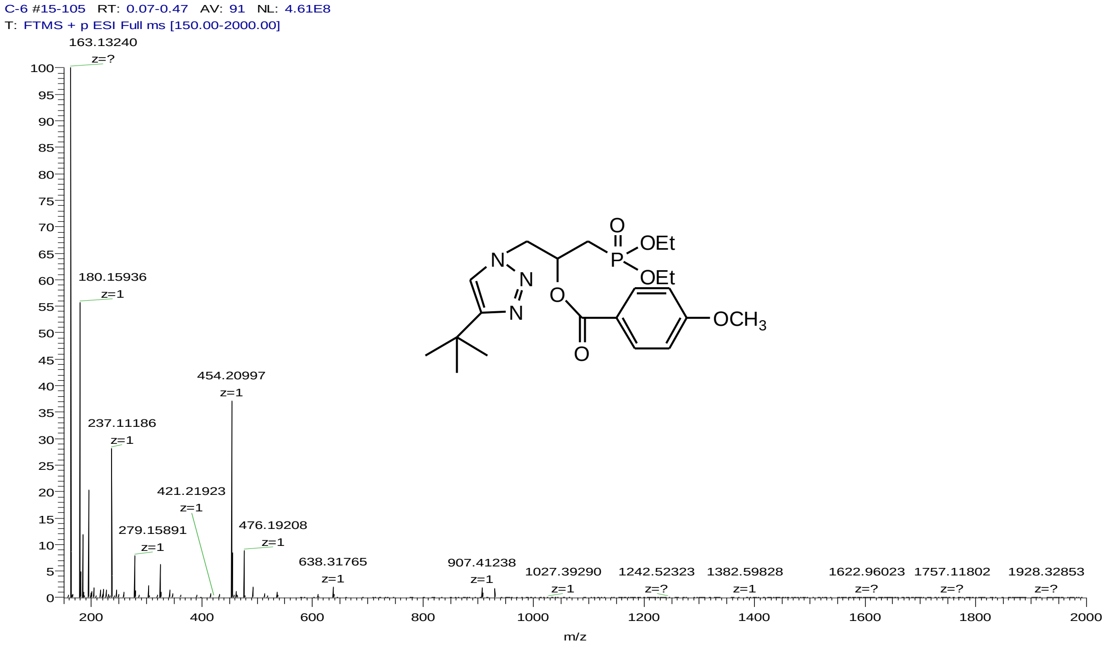

Figure S109. HRMS spectrum of compound **5-C6**.

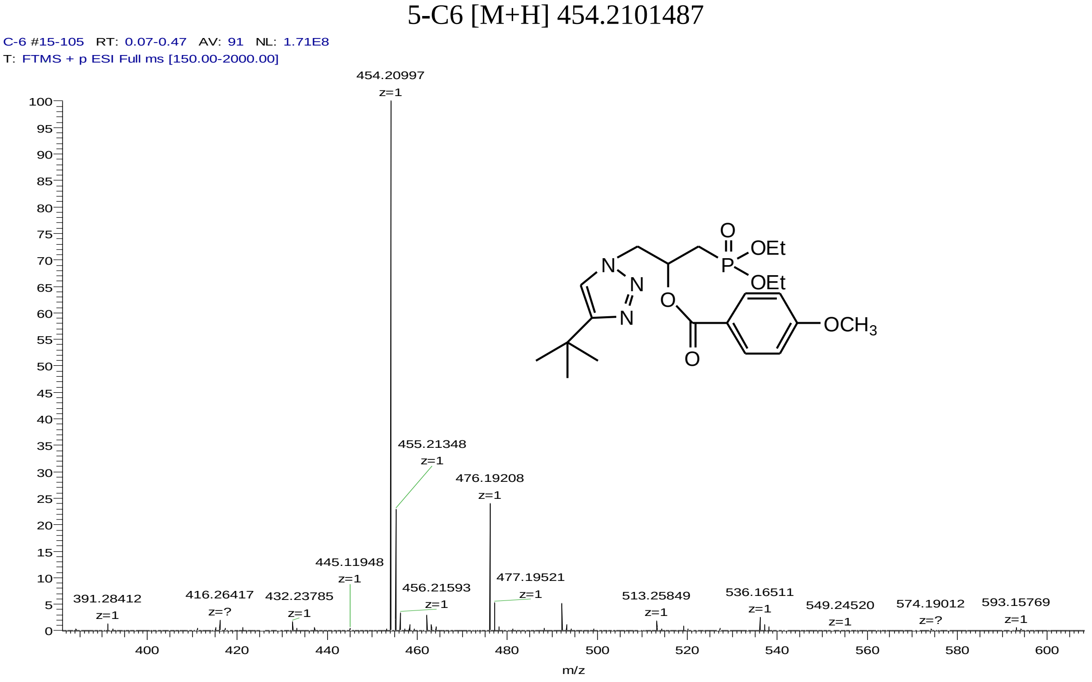

Figure S110. ^{1}H-NMR spectrum of compound **5-C7**.

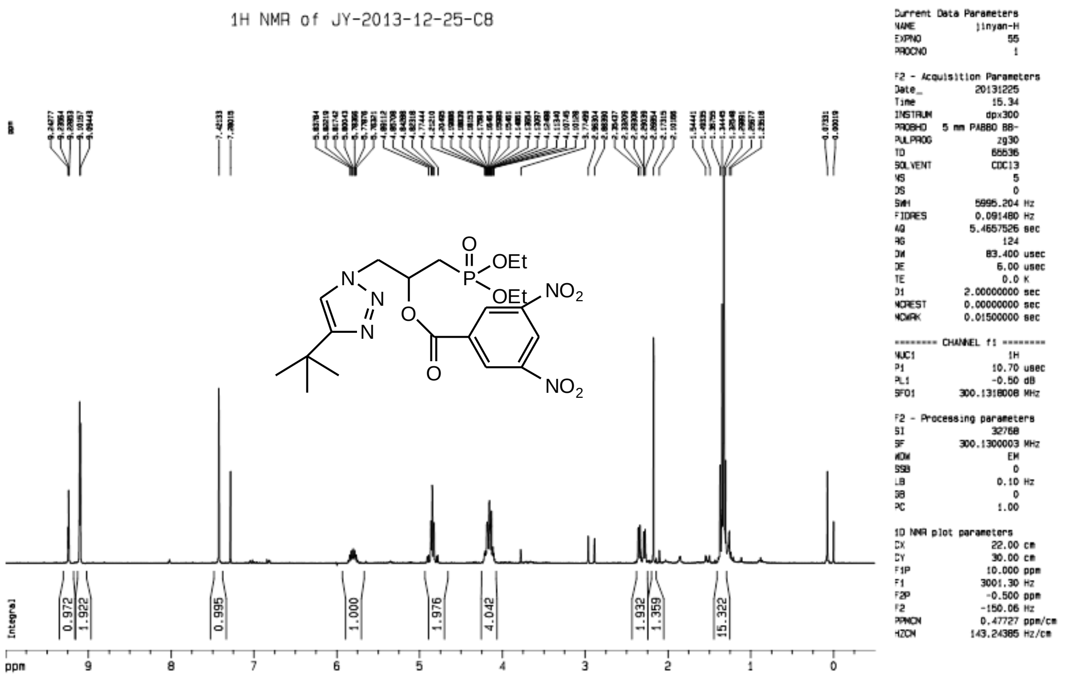

Figure S111. ^{13}C-NMR spectrum of compound **5-C7**.

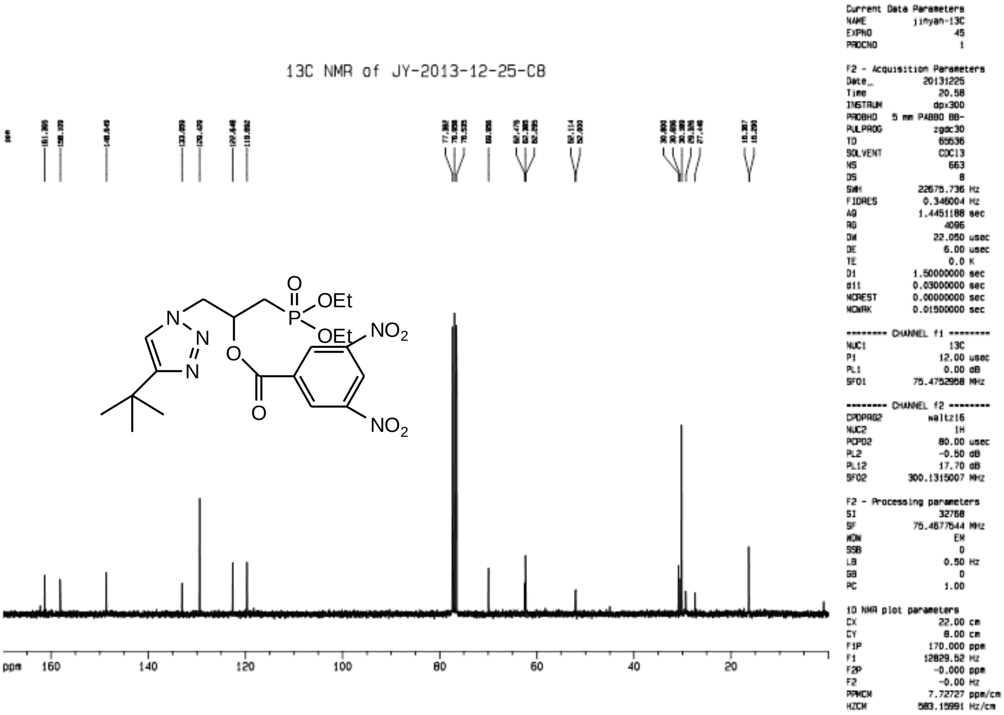

Figure S112. ^{31}P-NMR spectrum of compound **5-C7**.

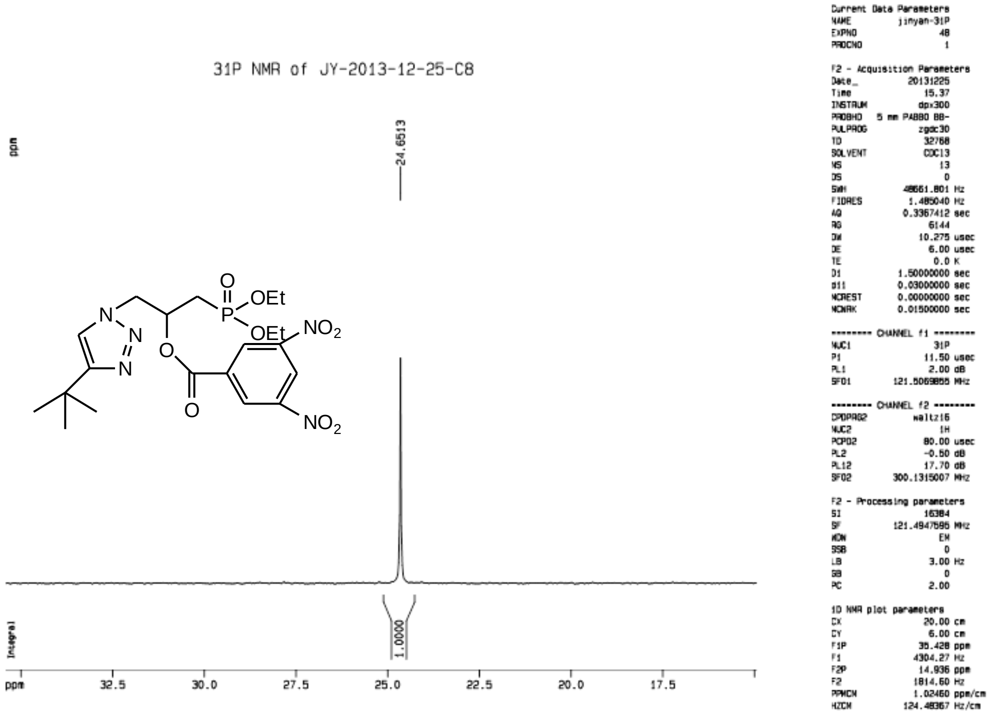

Figure S113. HRMS spectrum of compound **5-C7**.

5-C7 [M+H]514.1697404

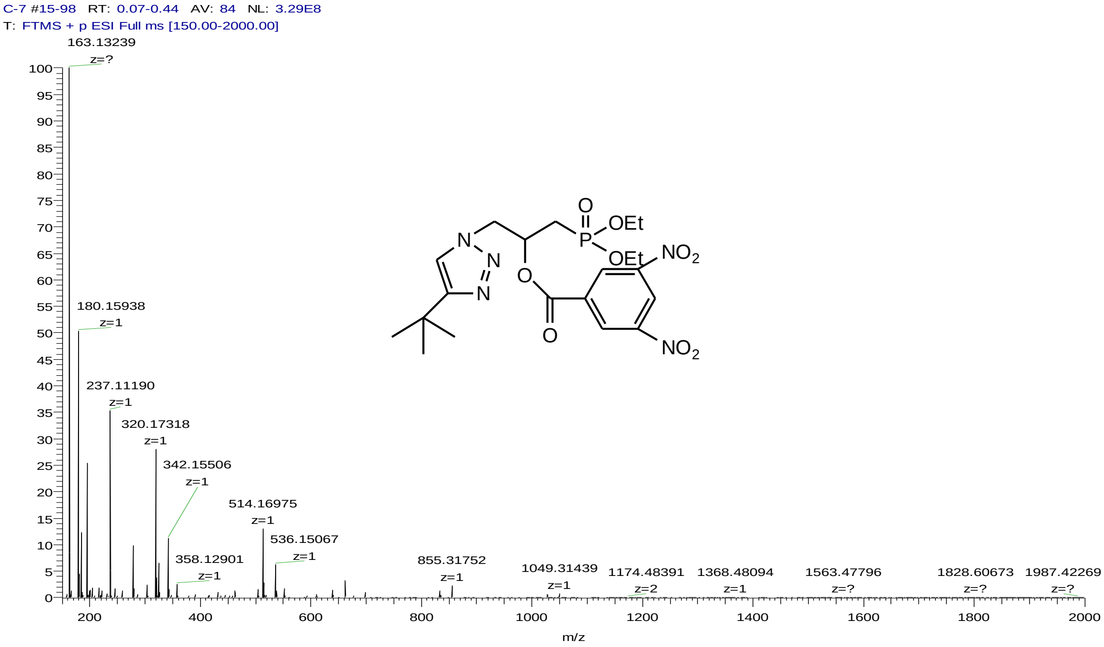

Figure S114. HRMS spectrum of compound **5-C7**.

5-C7 [M+H] 514.1697404

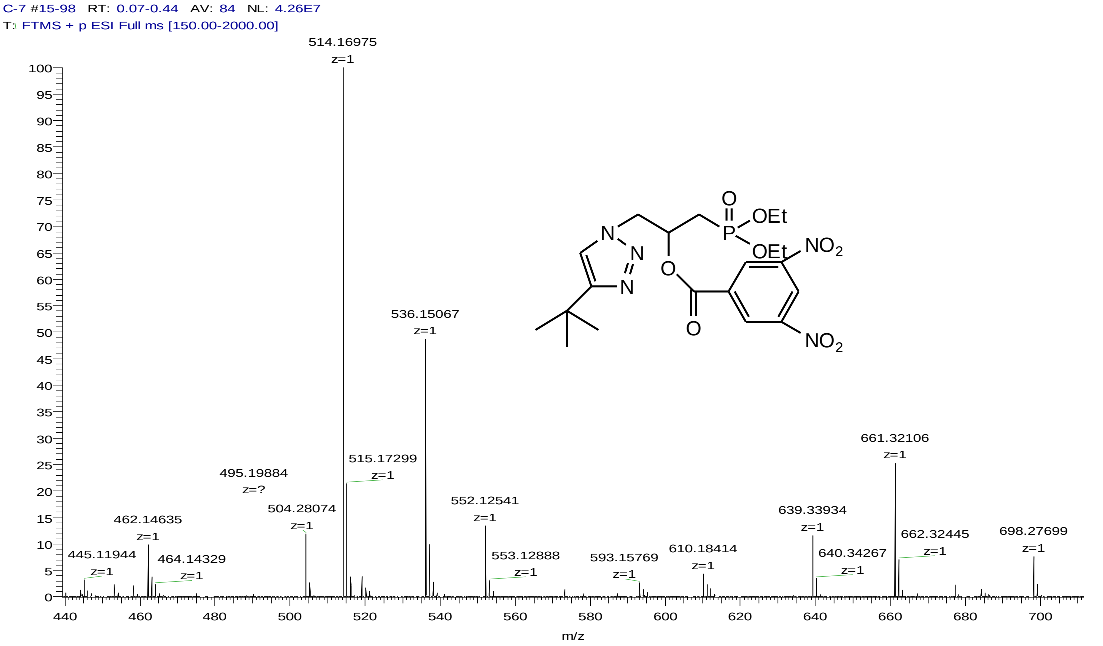

Figure S115. ^{1}H-NMR spectrum of compound **5-D1**.

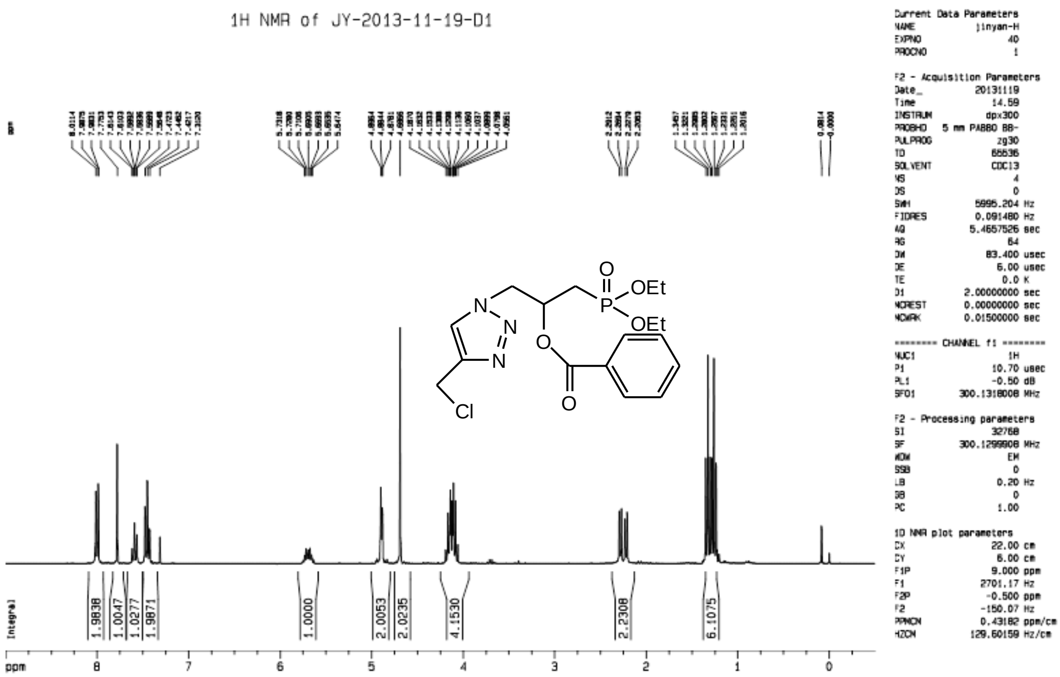

Figure S116. ^{13}C-NMR spectrum of compound **5-D1**.

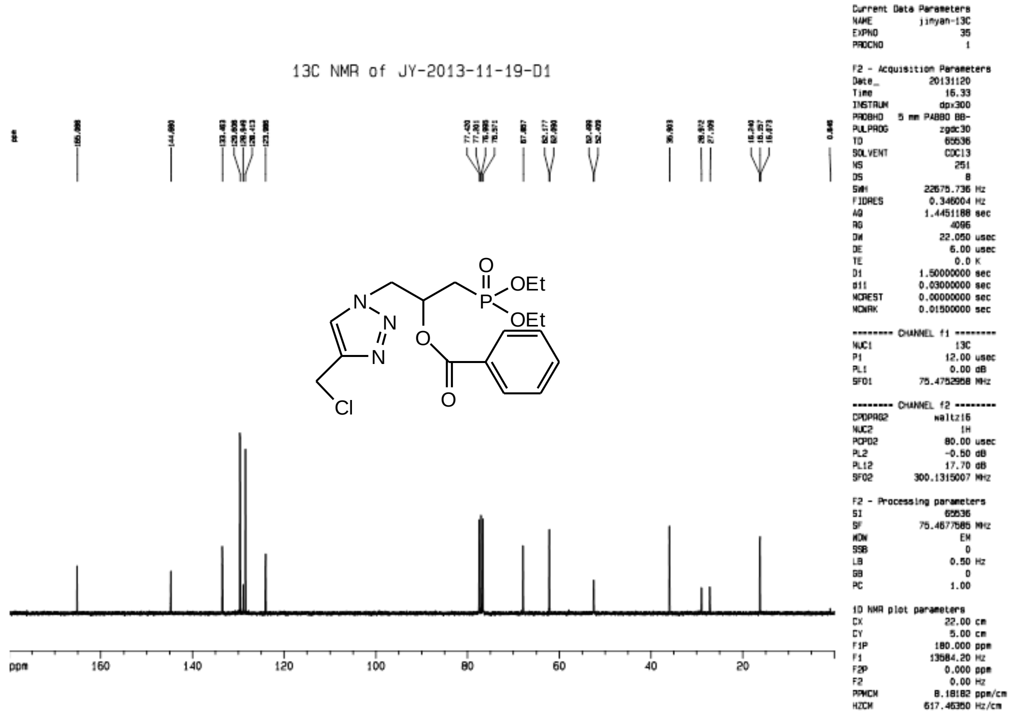

Figure S117. ^{31}P-NMR spectrum of compound **5-D1**.

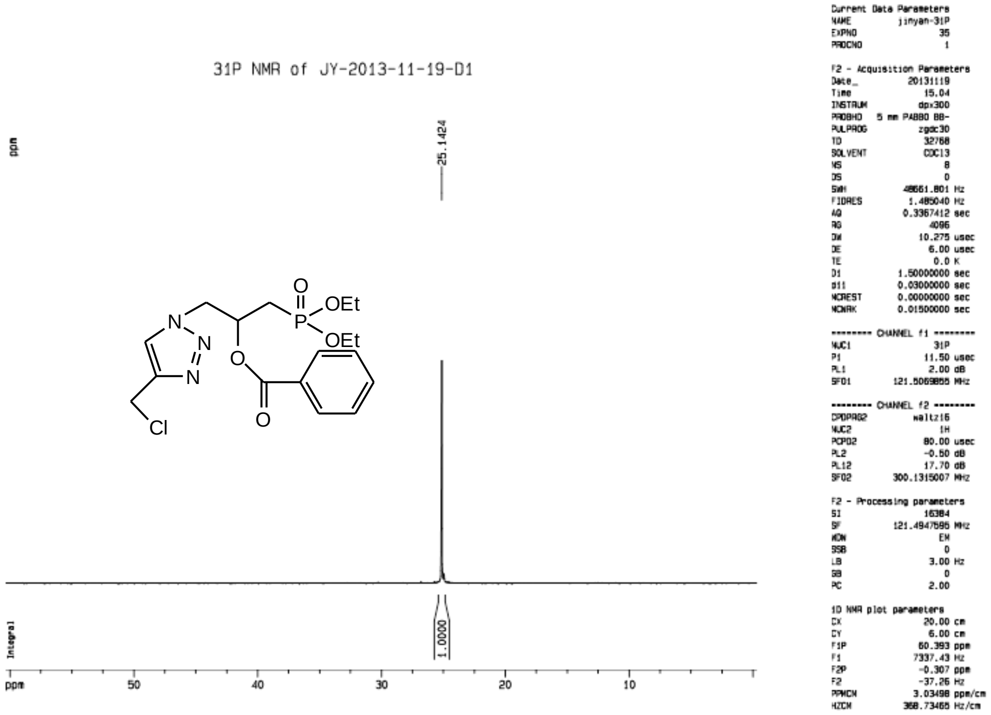

Figure S118. HRMS spectrum of compound **5-D1**.

5-D1 [M+H] 416.1136615

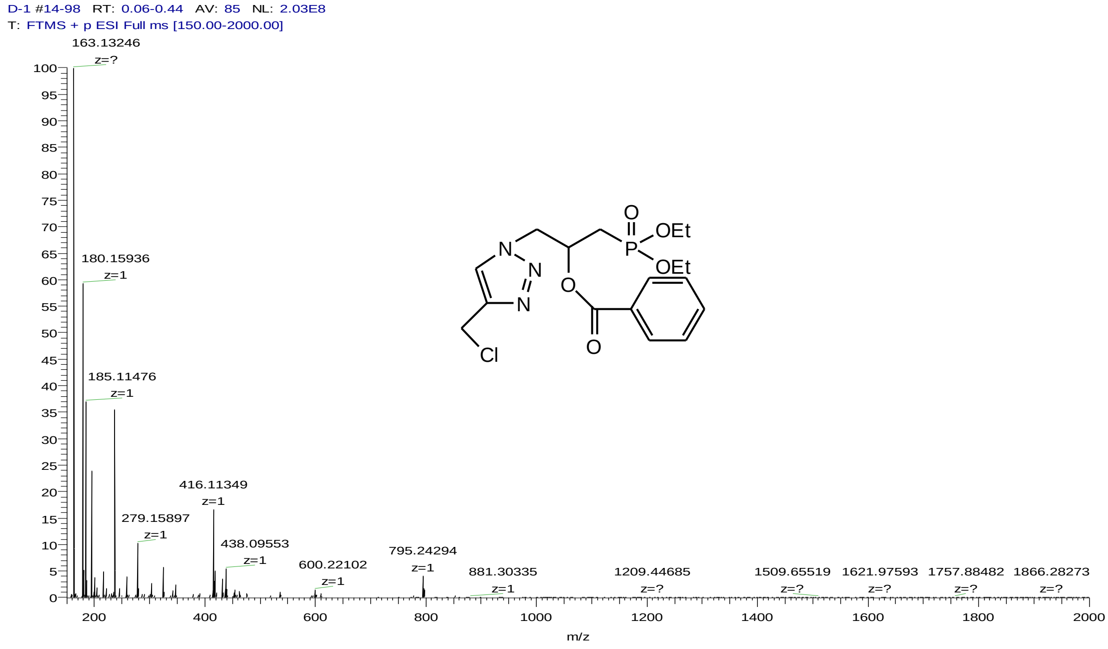

Figure S119. HRMS spectrum of compound **5-D1**.

5-D1 [M+H] 416.1136615

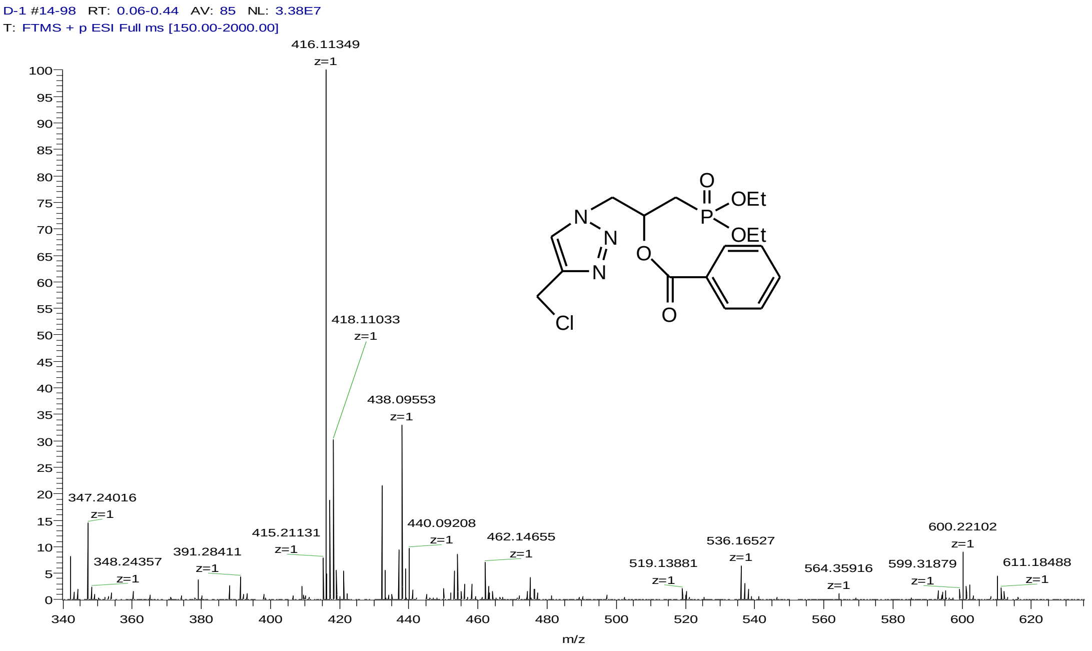

Figure S120. ^{1}H-NMR spectrum of compound **5-D2**.

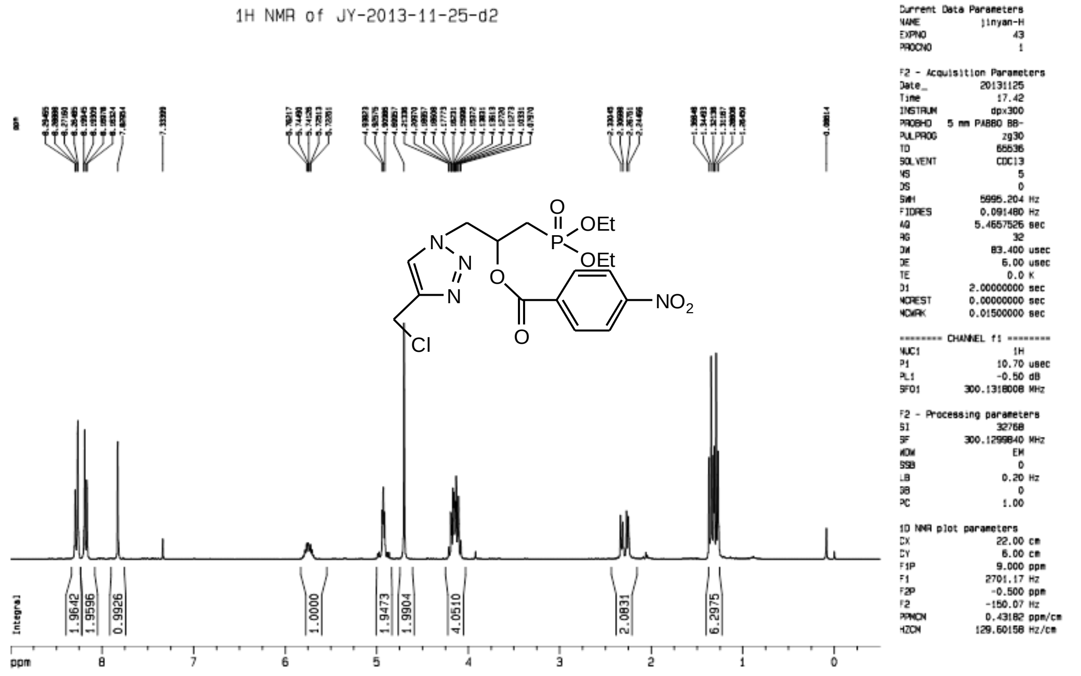

Figure S121. ^{13}C-NMR spectrum of compound **5-D2**.

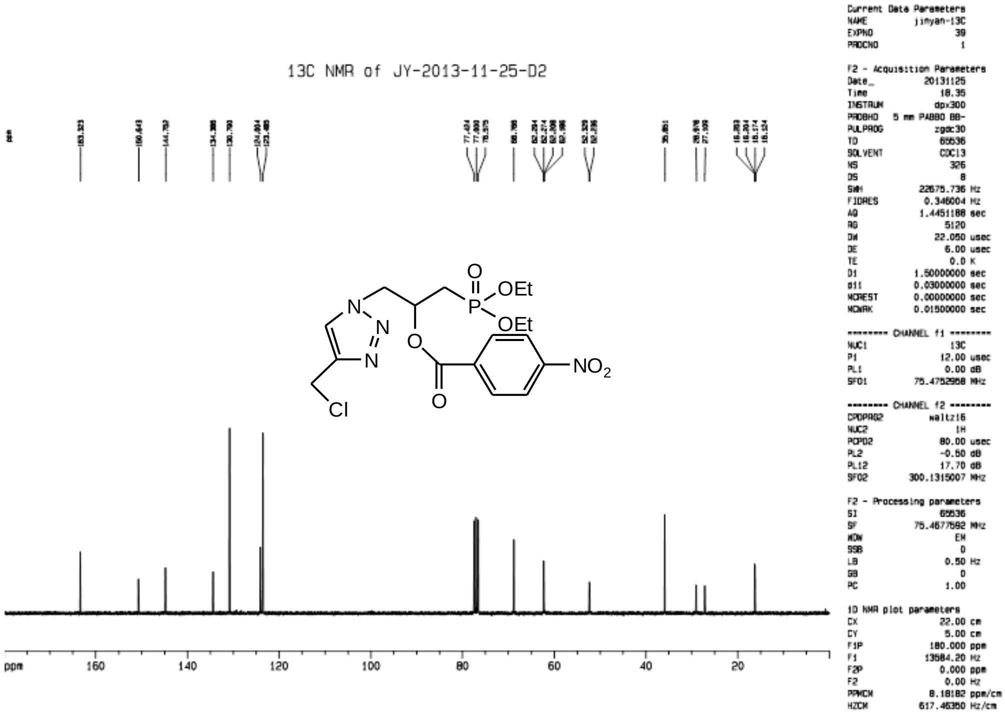

Figure S122. ^{31}P-NMR spectrum of compound **5-D2**.

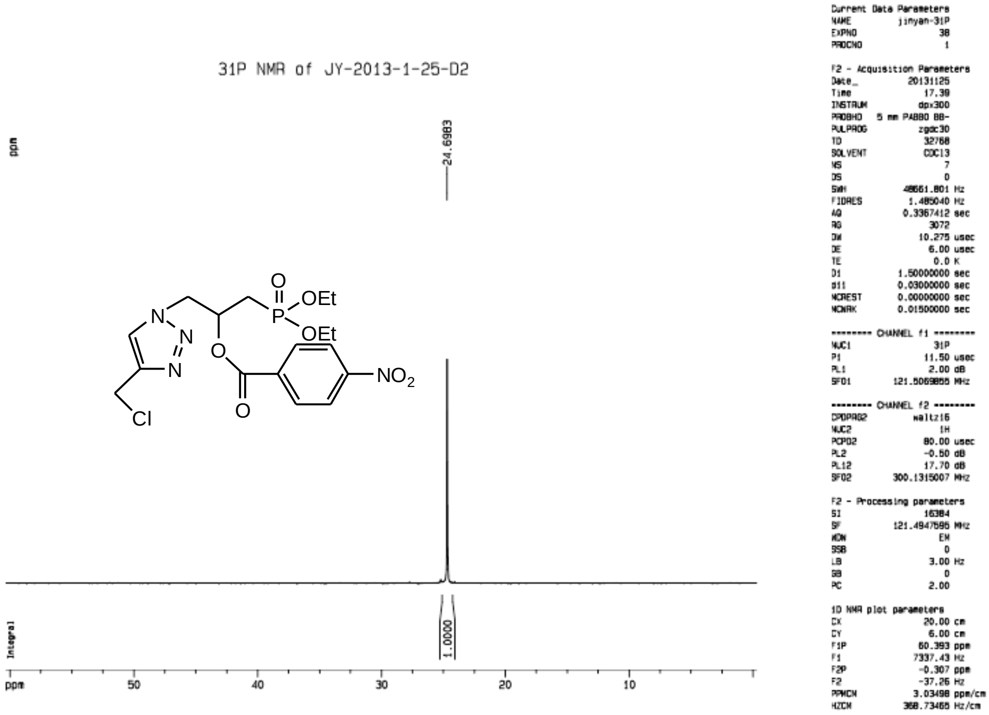

Figure S123. HRMS spectrum of compound **5-D2**.

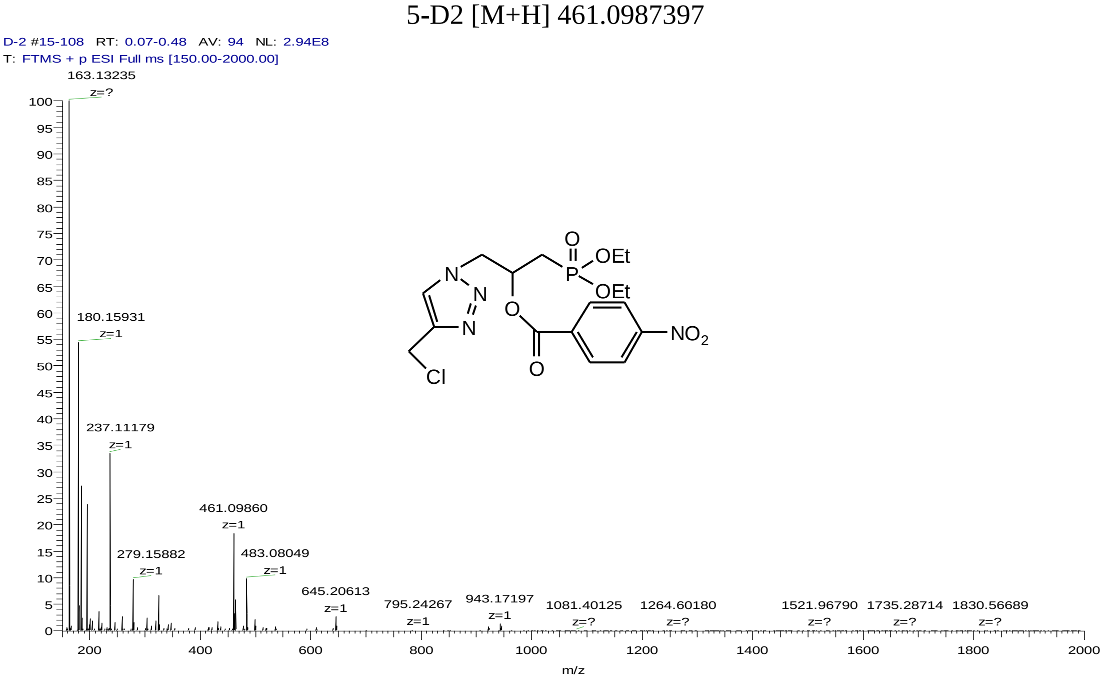

Figure S124. HRMS spectrum of compound **5-D2**.

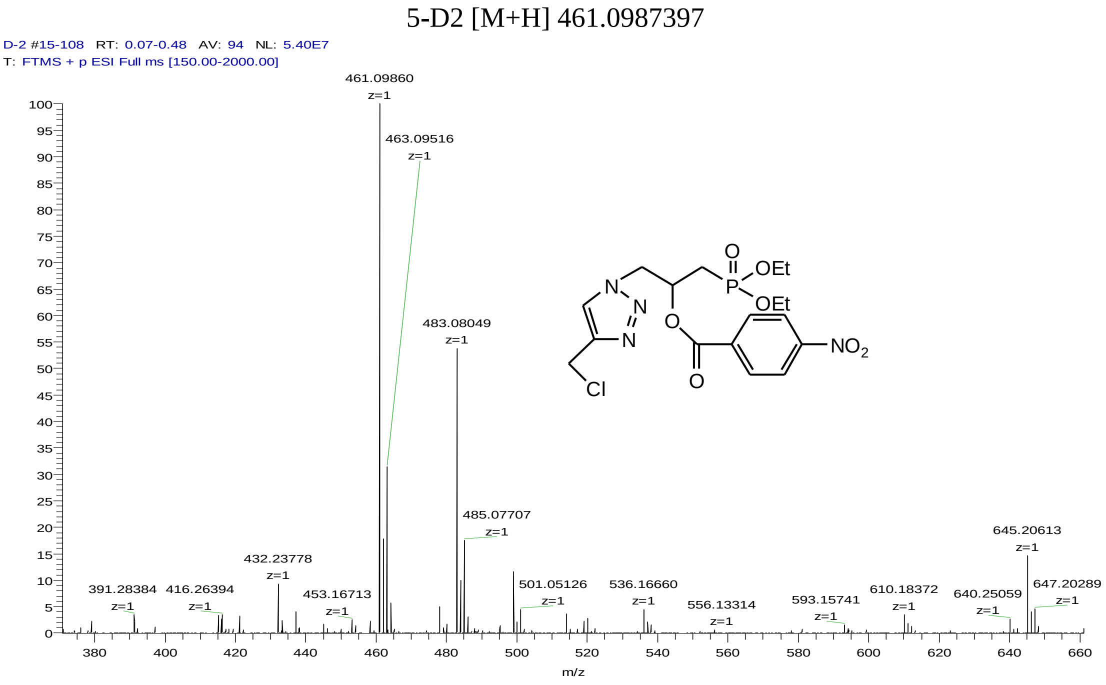

Figure S125. ^{1}H-NMR spectrum of compound **5-D3**.

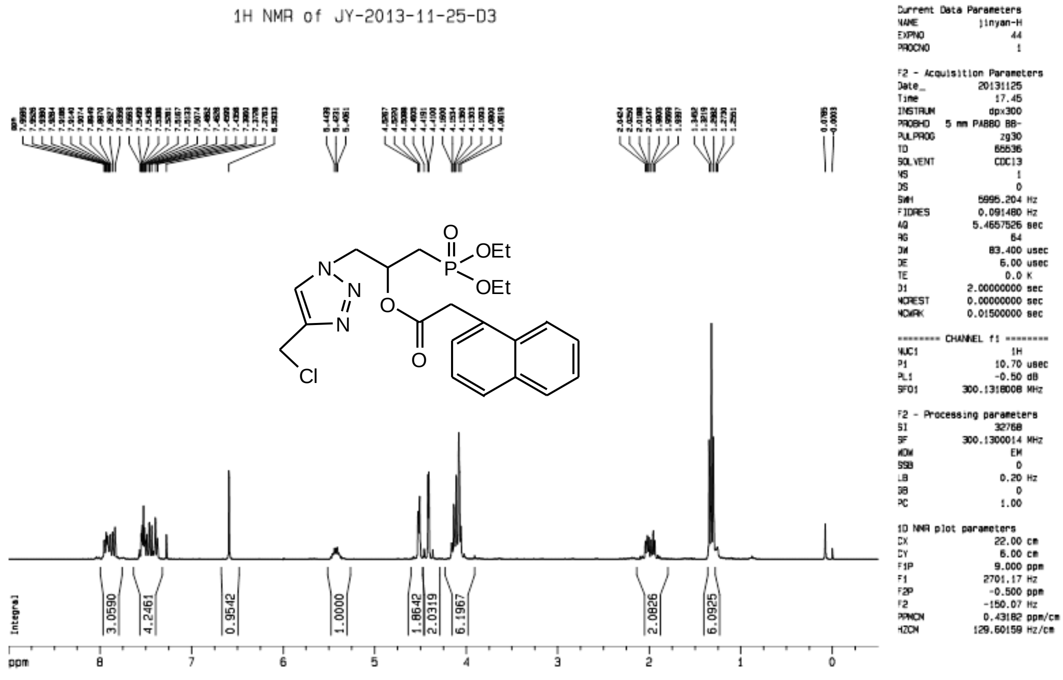

Figure S126. ^{13}C-NMR spectrum of compound **5-D3**.

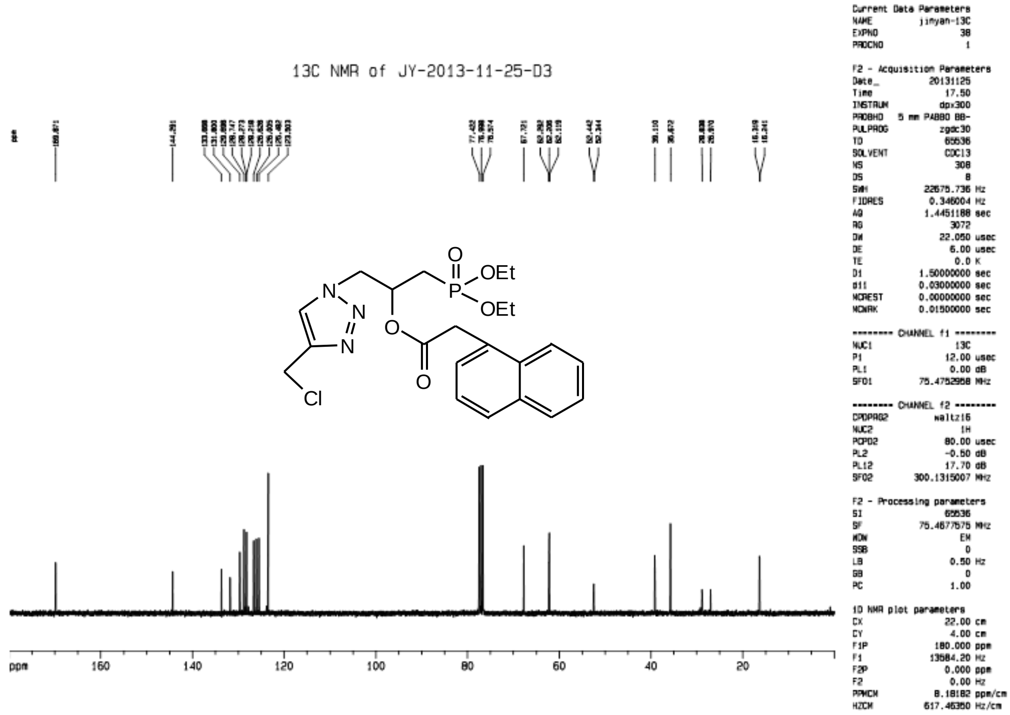

Figure S127. ^{31}P-NMR spectrum of compound **5-D3**.

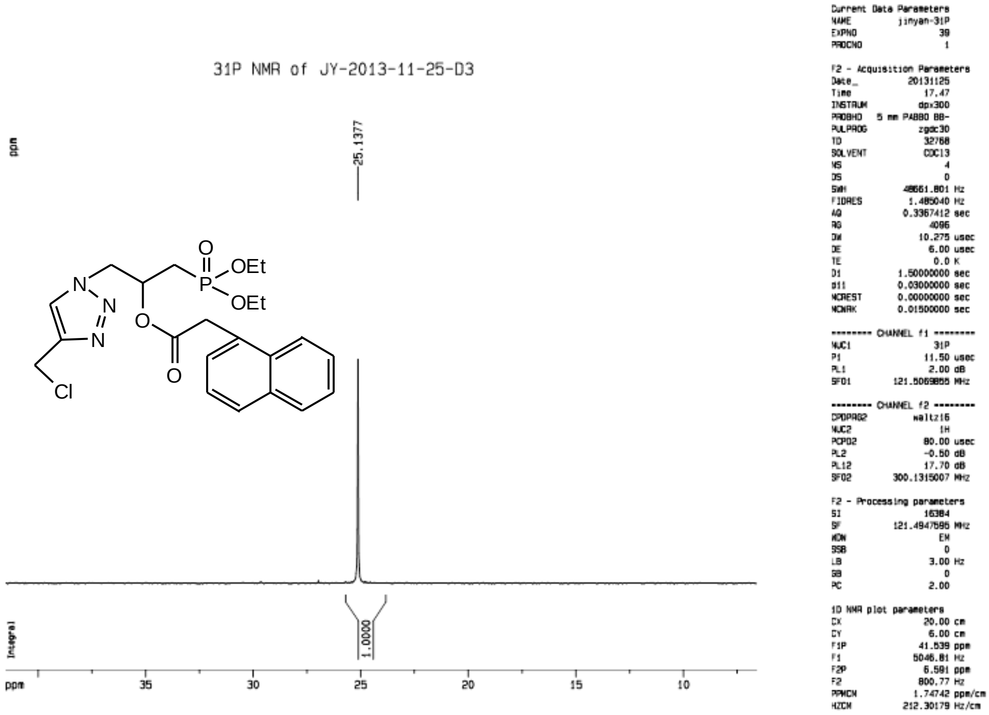

Figure S128. HRMS spectrum of compound **5-D3**.

5-D3 [M+H]480.1449616

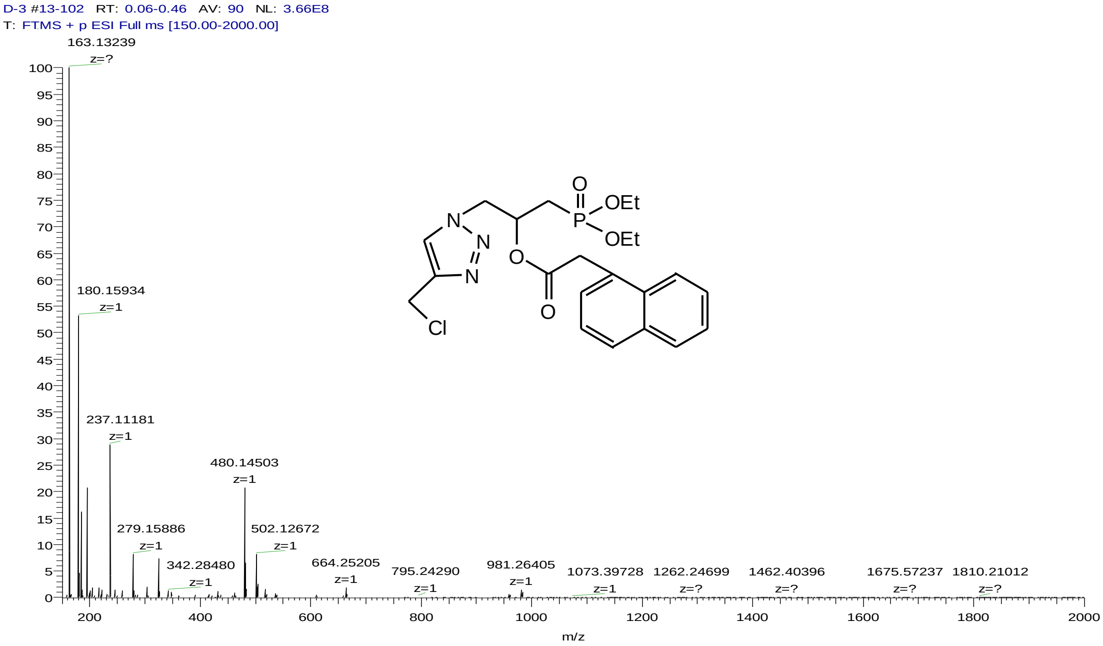

Figure S129. HRMS spectrum of compound **5-D3**.

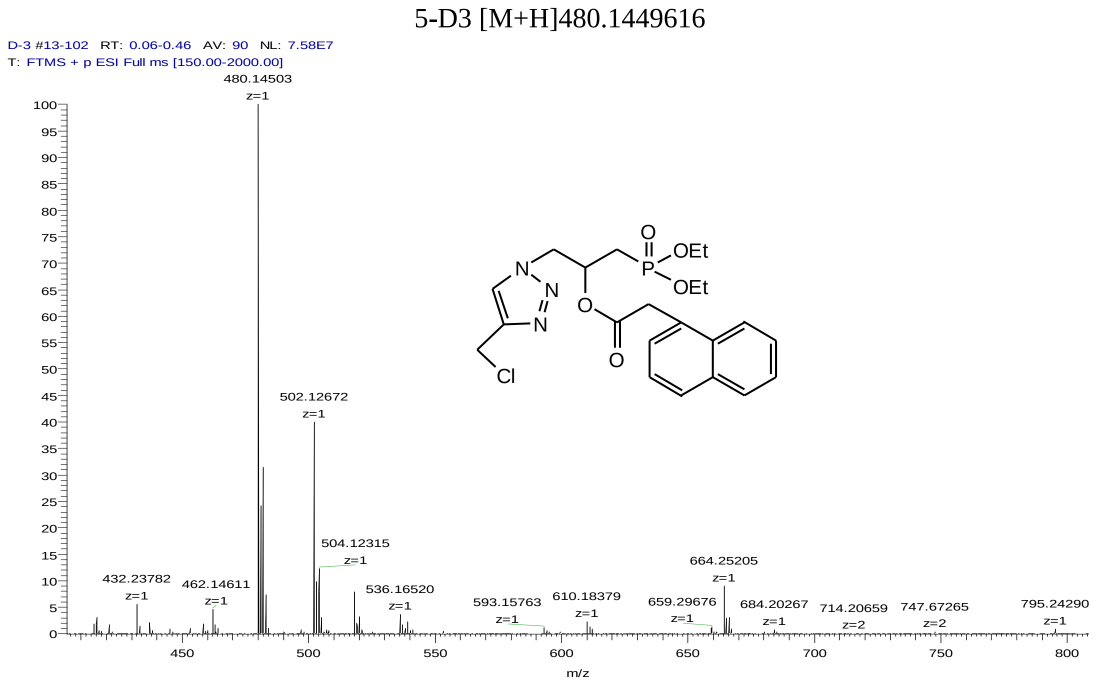

Figure S130. ^{1}H-NMR spectrum of compound **5-D4**.

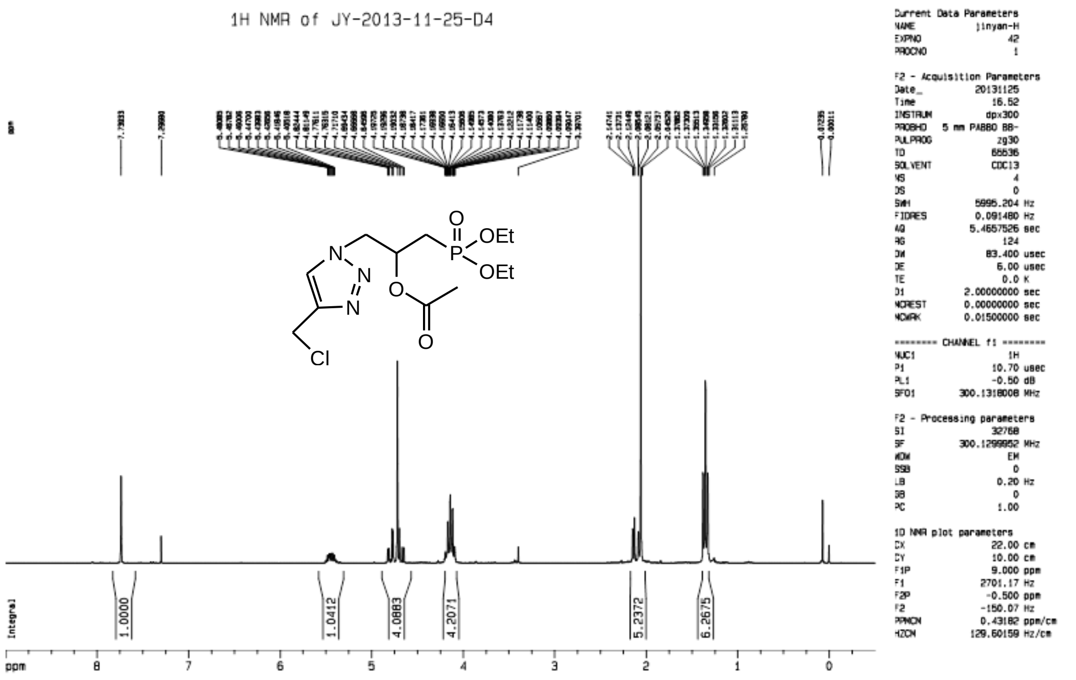

Figure S131. ^{13}C-NMR spectrum of compound **5-D4**.

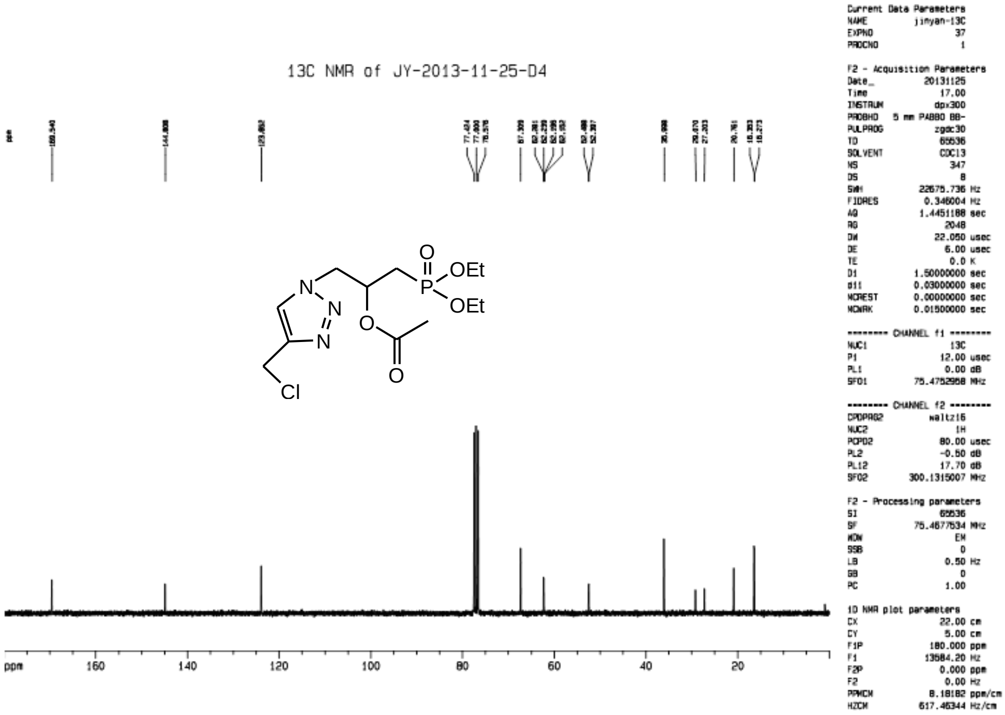

Figure S132. ^{31}P-NMR spectrum of compound **5-D4**.

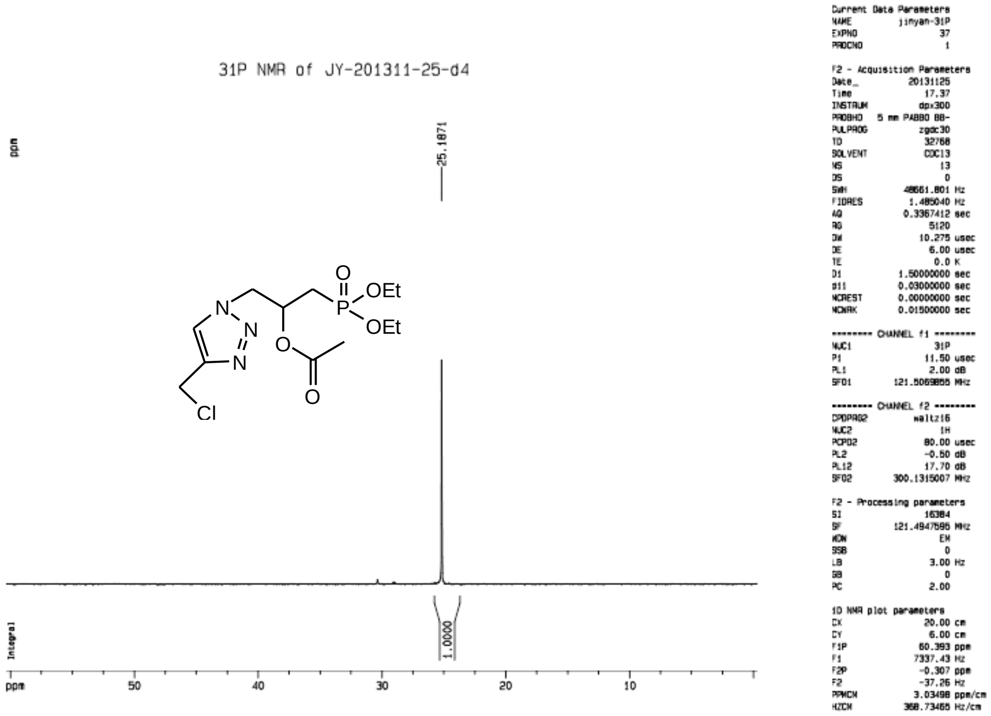

Figure S133. HRMS spectrum of compound **5-D4**.

5-D4 [M+H]354.0980115

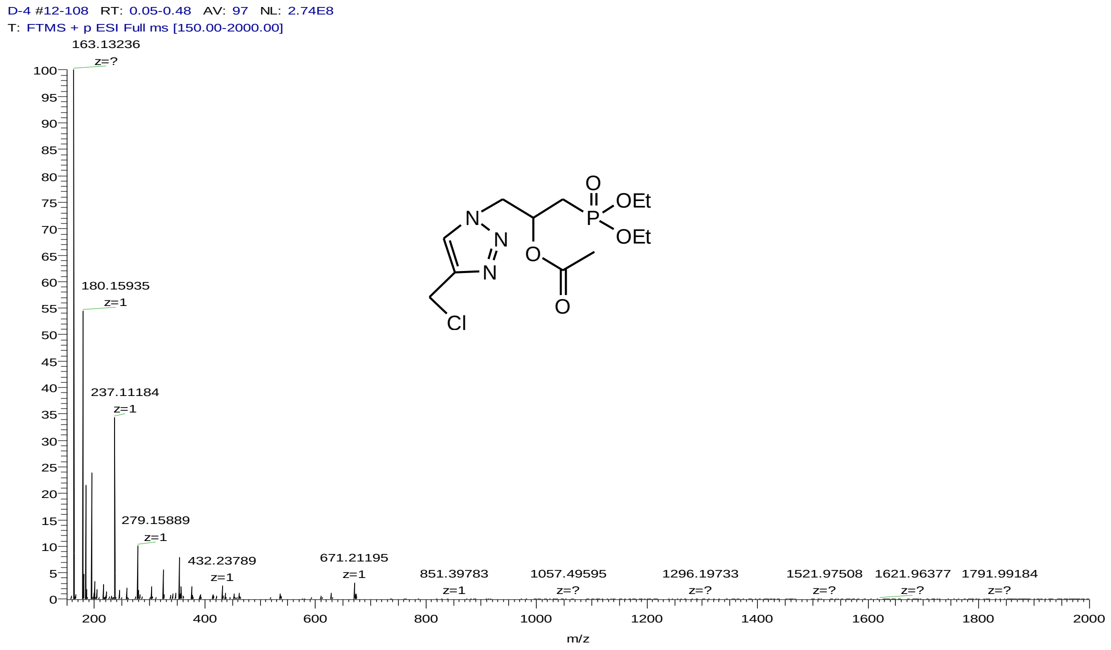

Figure S134. HRMS spectrum of compound **5-D4**.

5-D4 [M+H]354.0980115

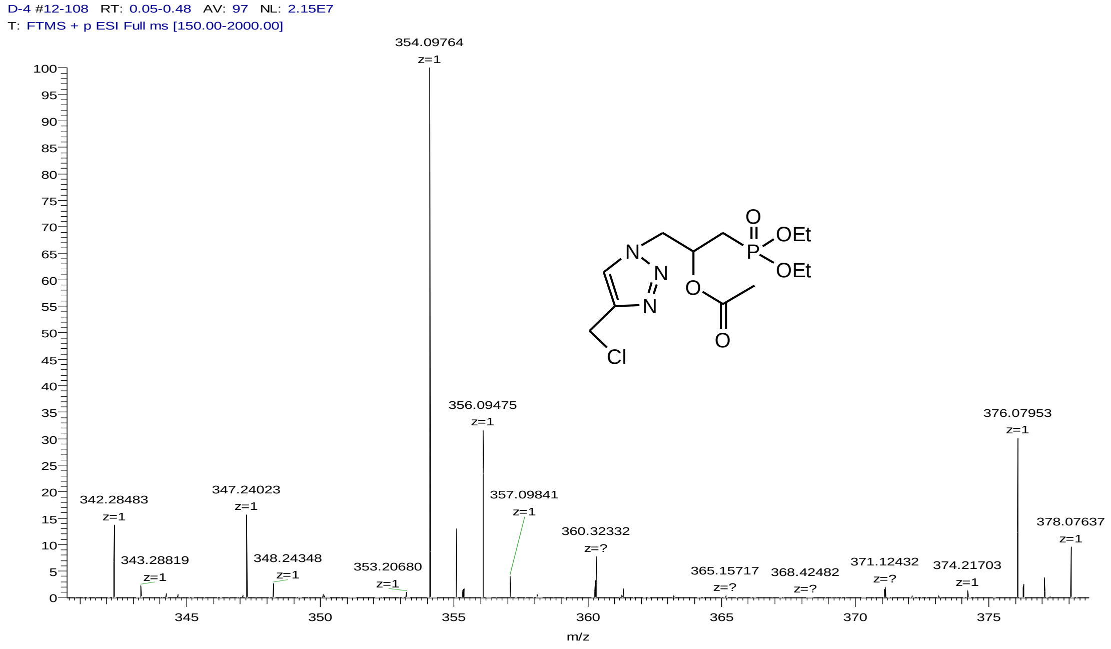

Figure S135. ^{1}H-NMR spectrum of compound **5-D5**.

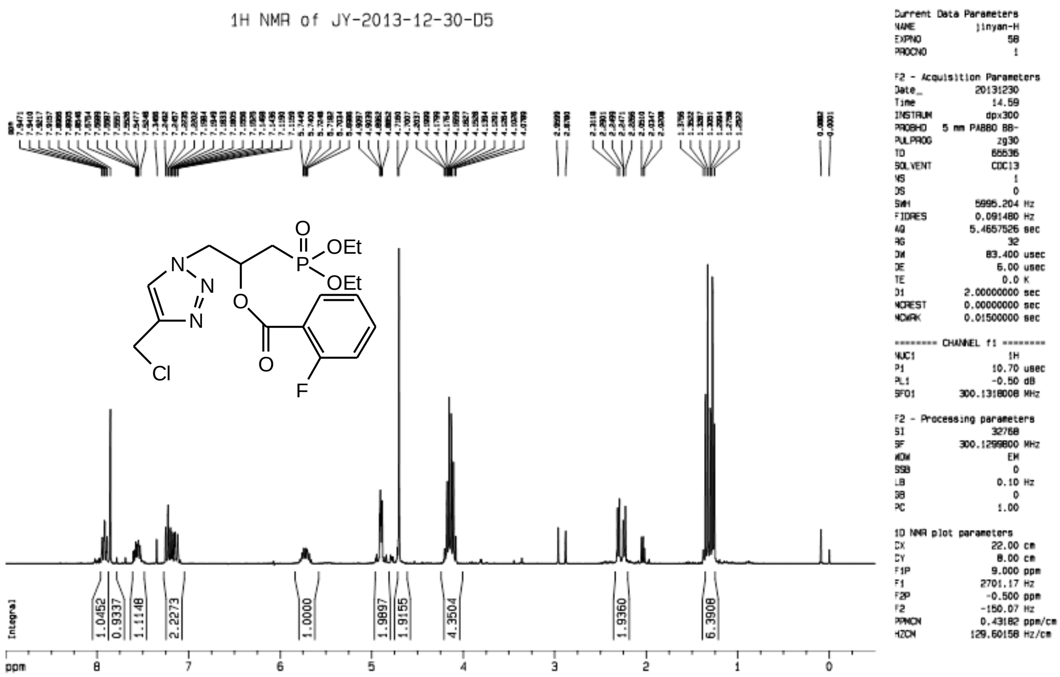

Figure S136. ^{13}C-NMR spectrum of compound **5-D5**.

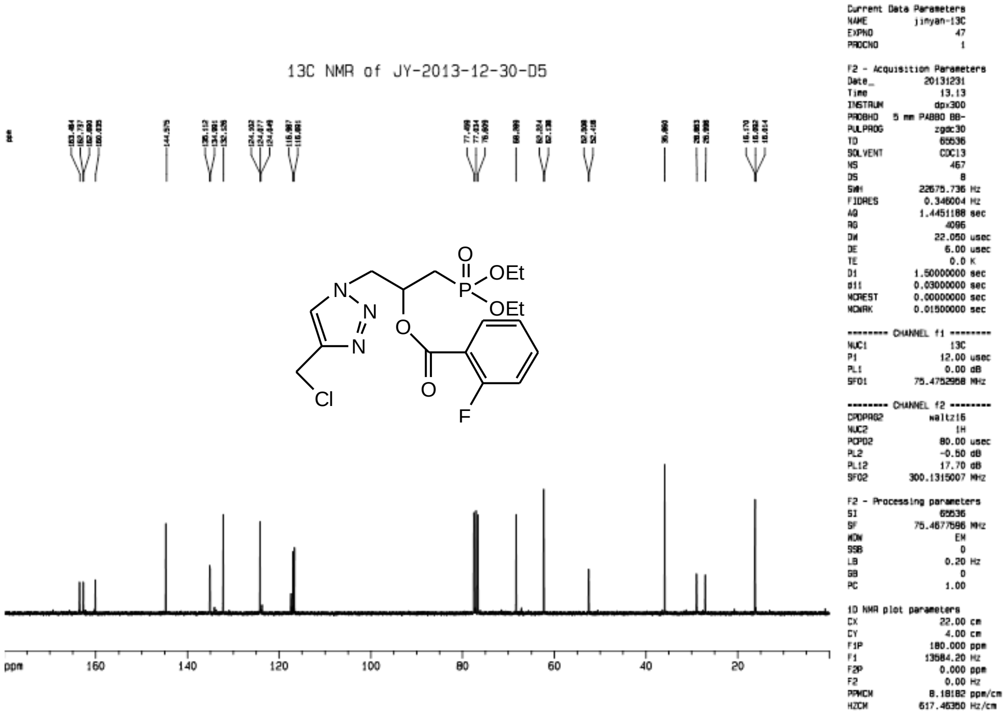

Figure S137. ^{31}P-NMR spectrum of compound **5-D5**.

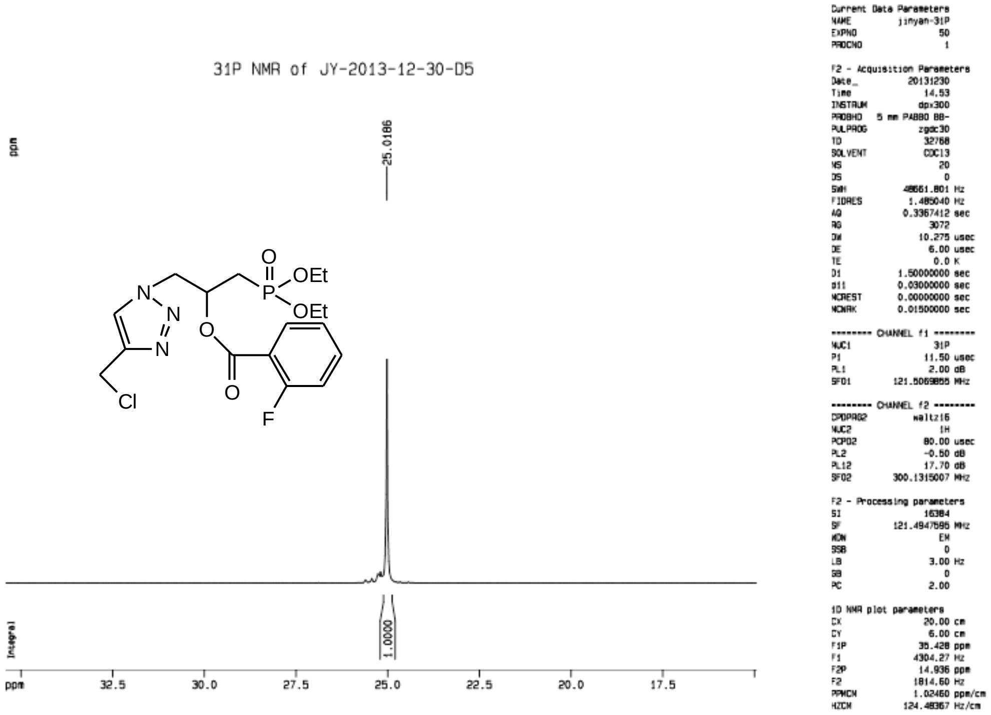

Figure S138. HRMS spectrum of compound **5-D5**.

5-D5 [M+H] 434.1042397

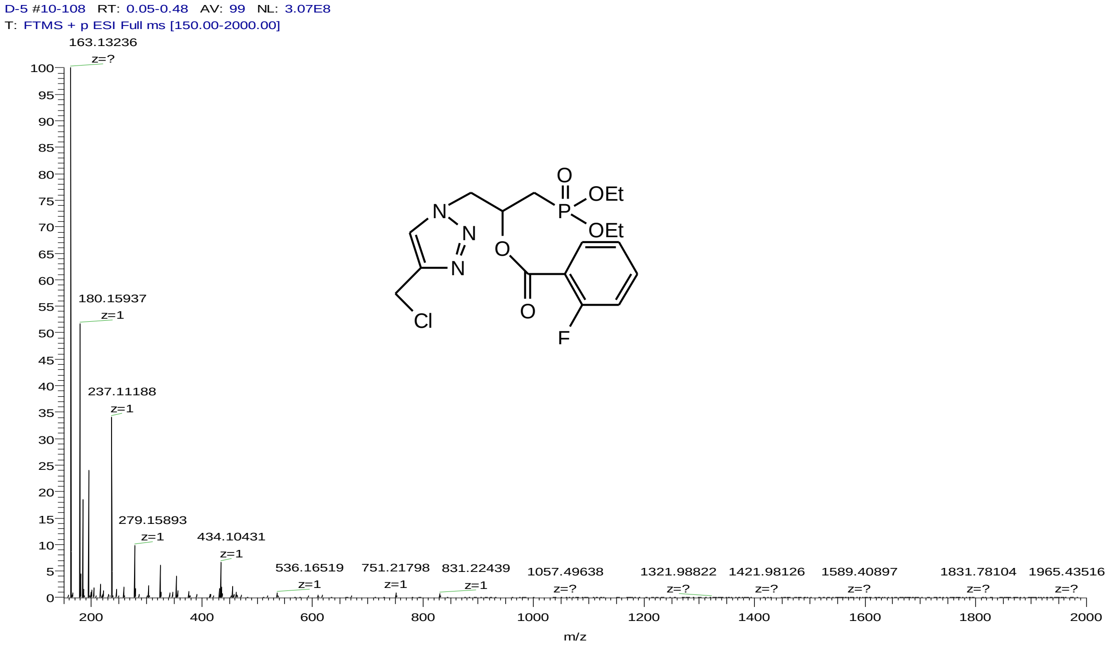

Figure S139. HRMS spectrum of compound **5-D5**.

5-D5 [M+H] 434.1042397

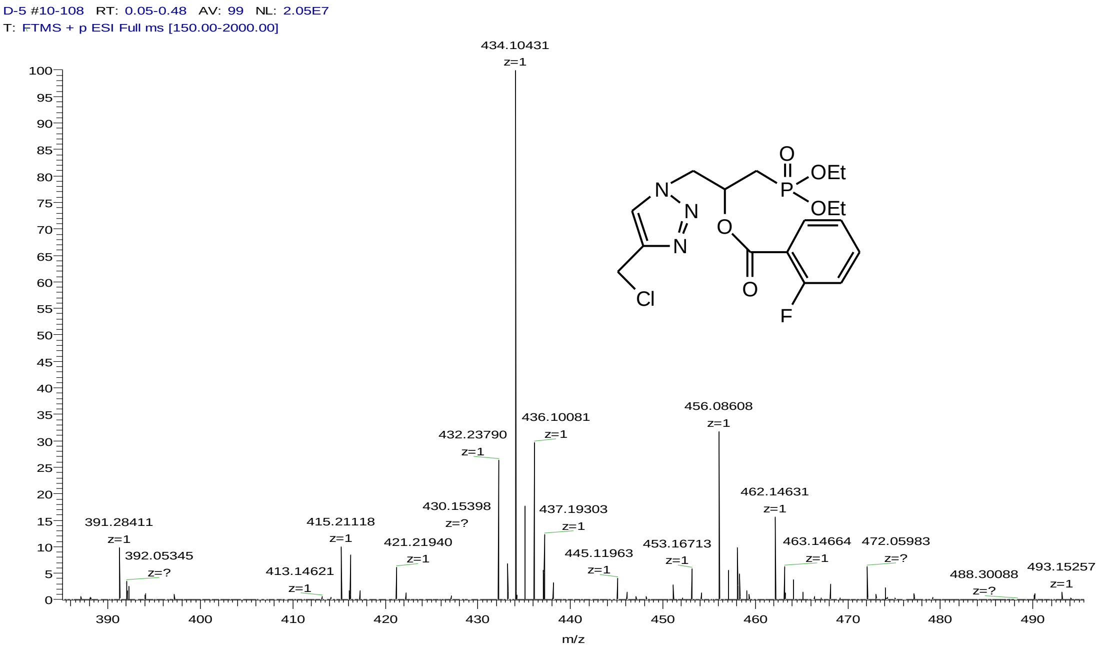

Figure S140. ^{1}H-NMR spectrum of compound **5-D6**.

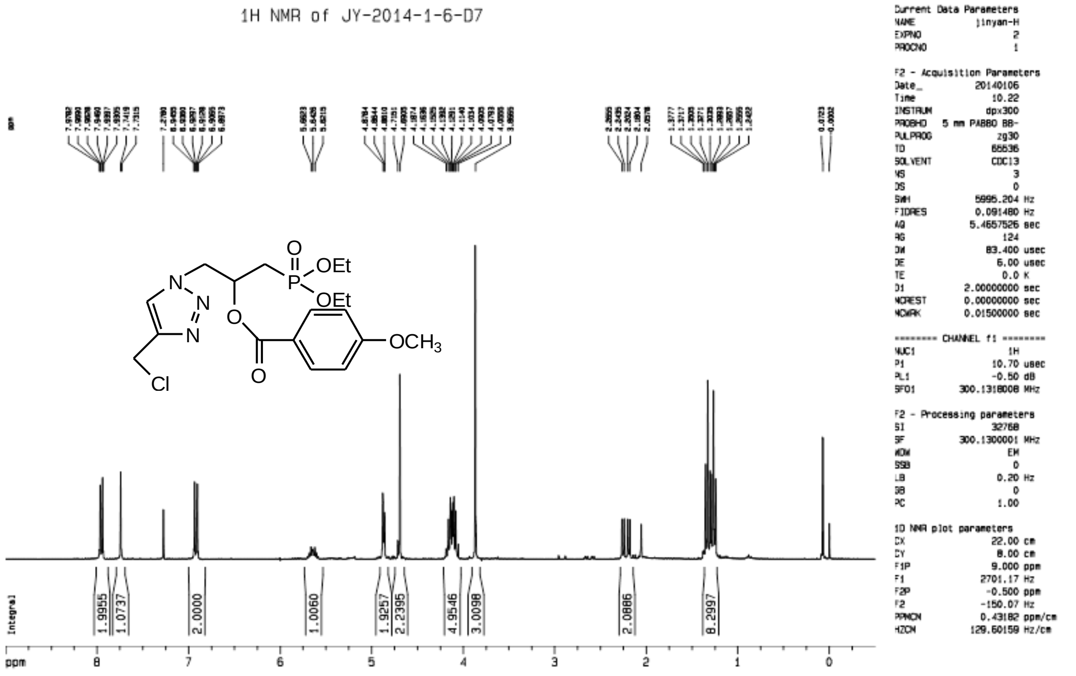

Figure S141. ^{13}C-NMR spectrum of compound **5-D6**.

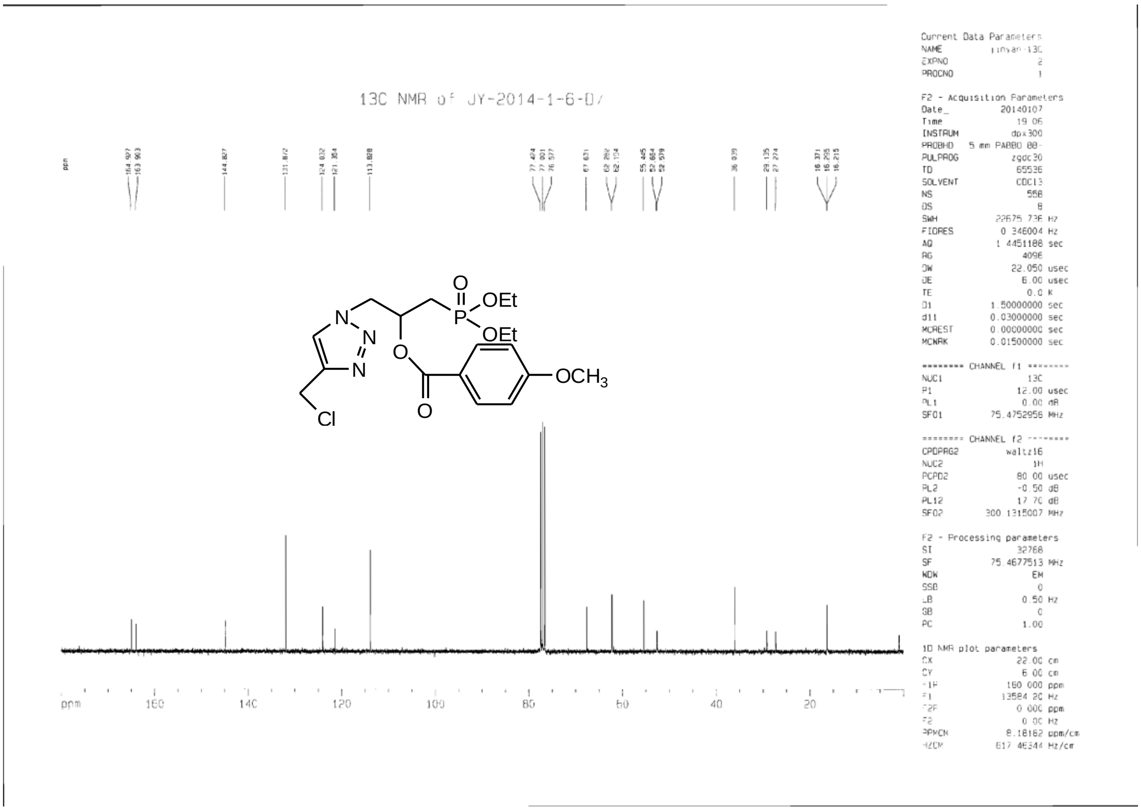

Figure S142. ^{31}P-NMR spectrum of compound **5-D6**.

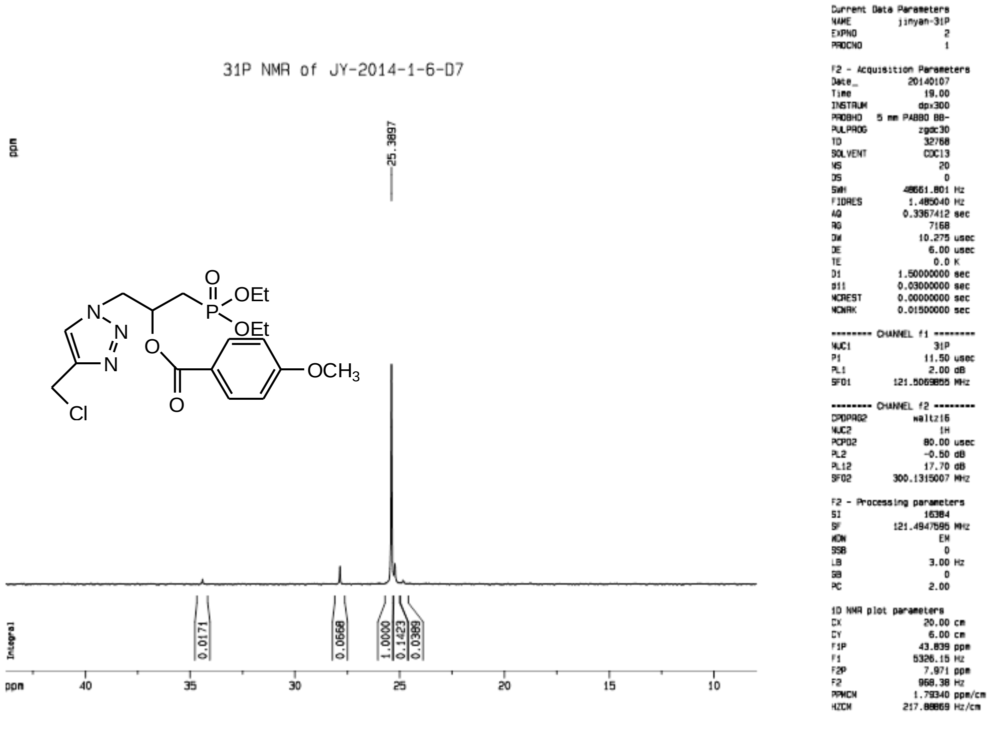

Figure S143. HRMS spectrum of compound **5-D6.**

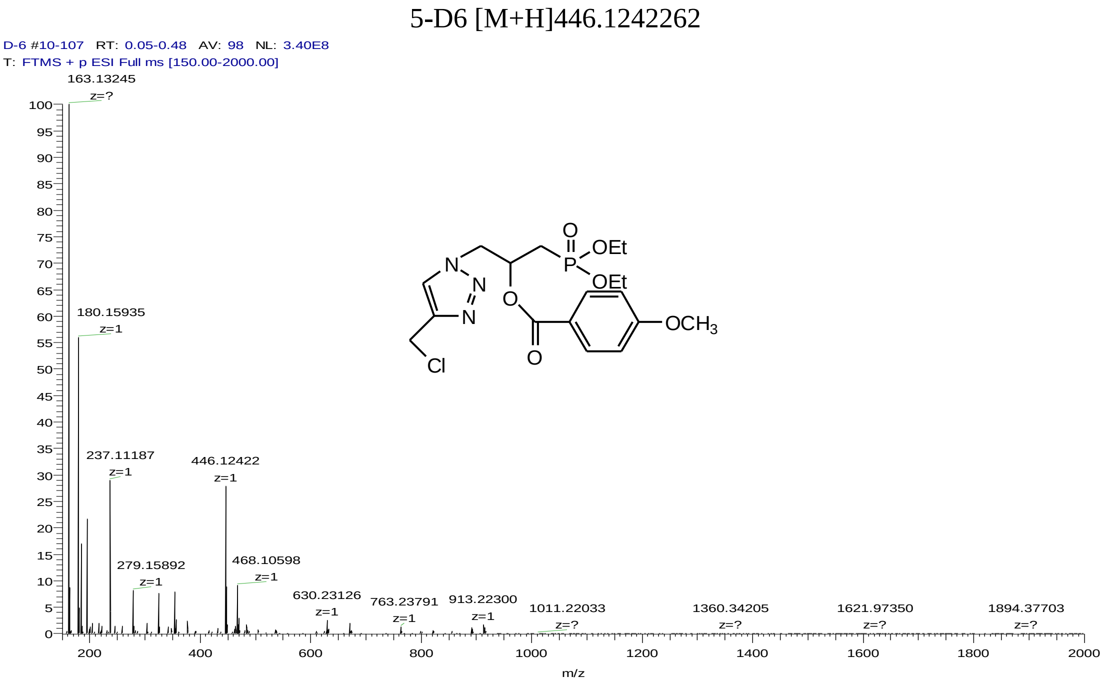

Figure S144. HRMS spectrum of compound **5-D6.**

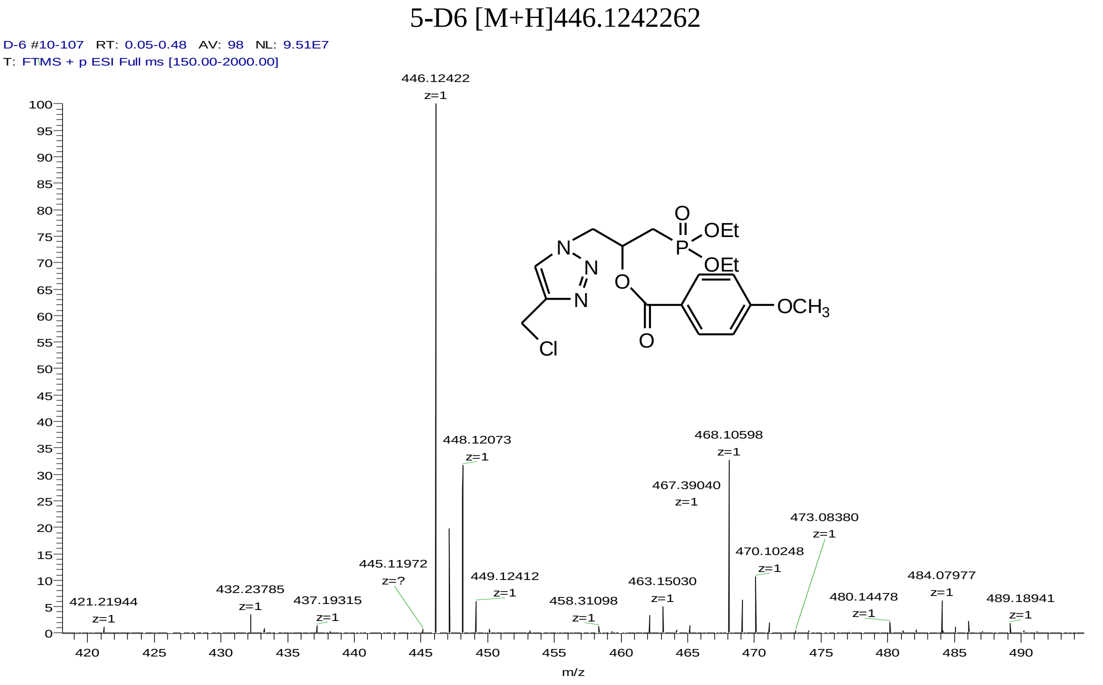

Figure S145. ^{1}H-NMR spectrum of compound **5-D7**.

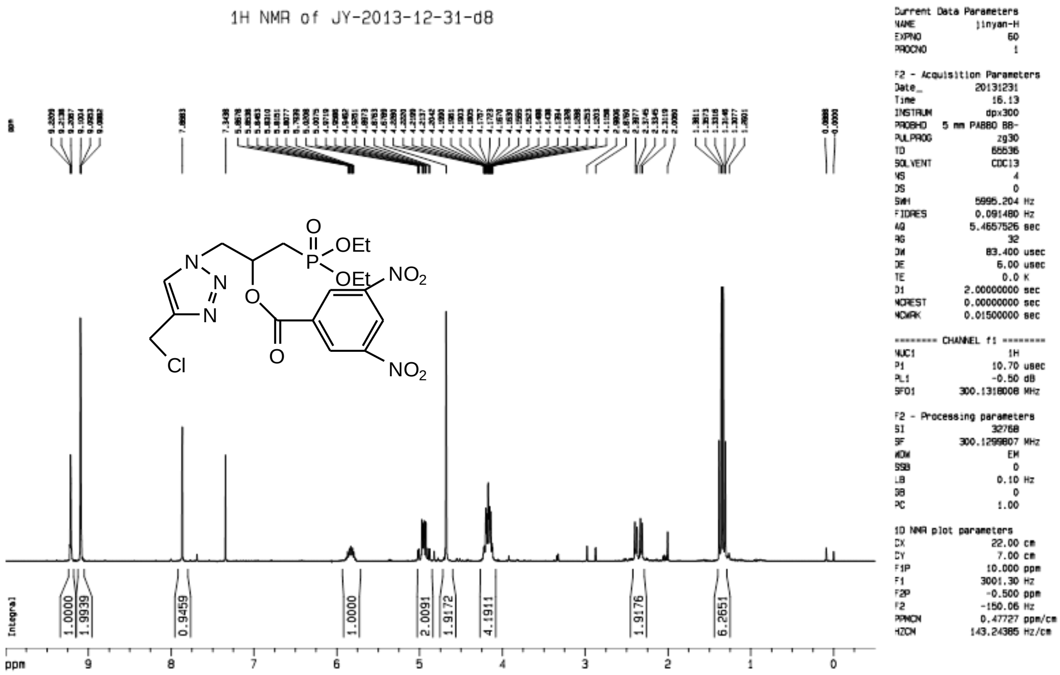

Figure S146. ^{13}C-NMR spectrum of compound **5-D7**.

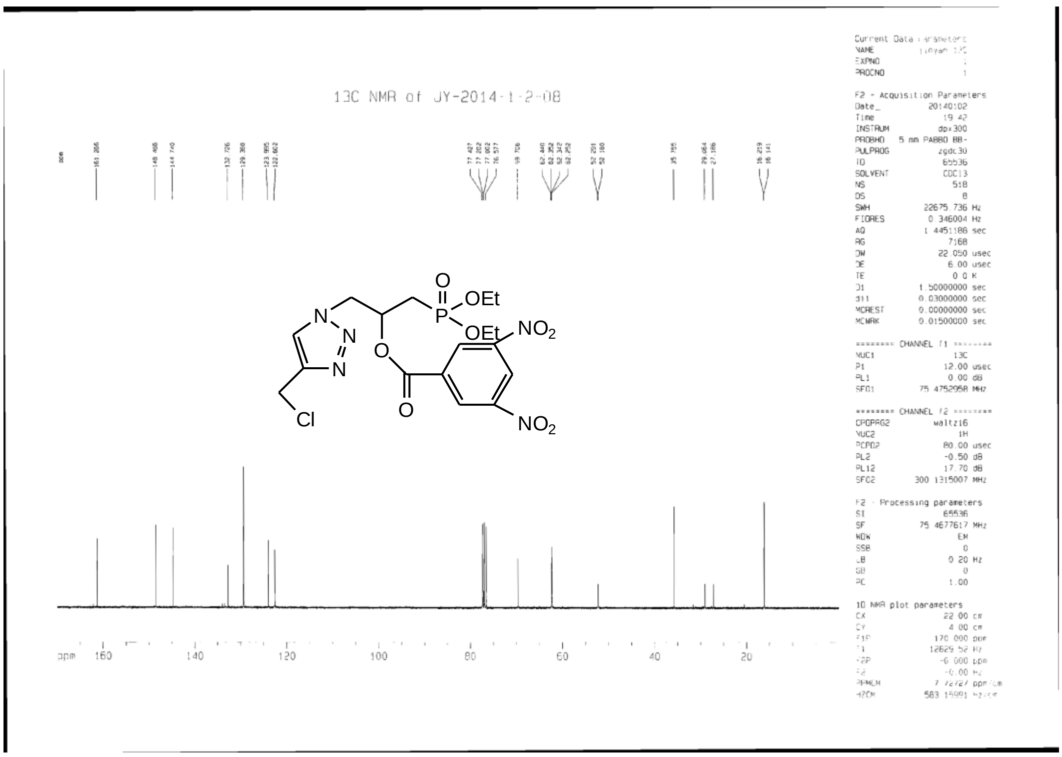

Figure S147. ^{31}P-NMR spectrum of compound **5-D7**.

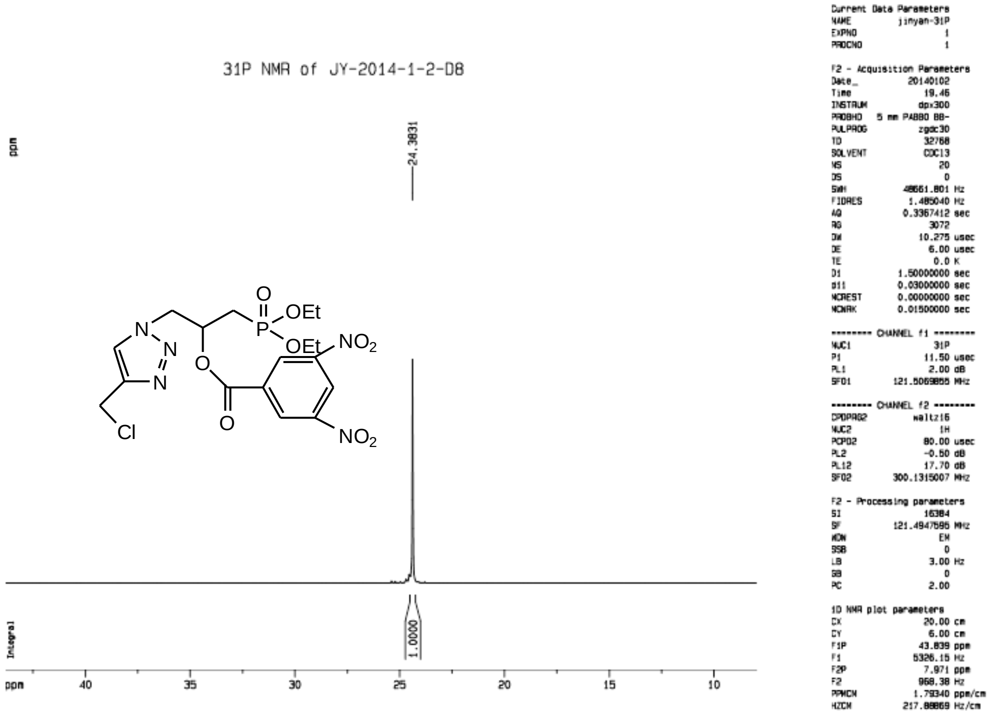

Figure S148. HRMS spectrum of compound **5-D7**.

5-D7 [M+H]506.0838179

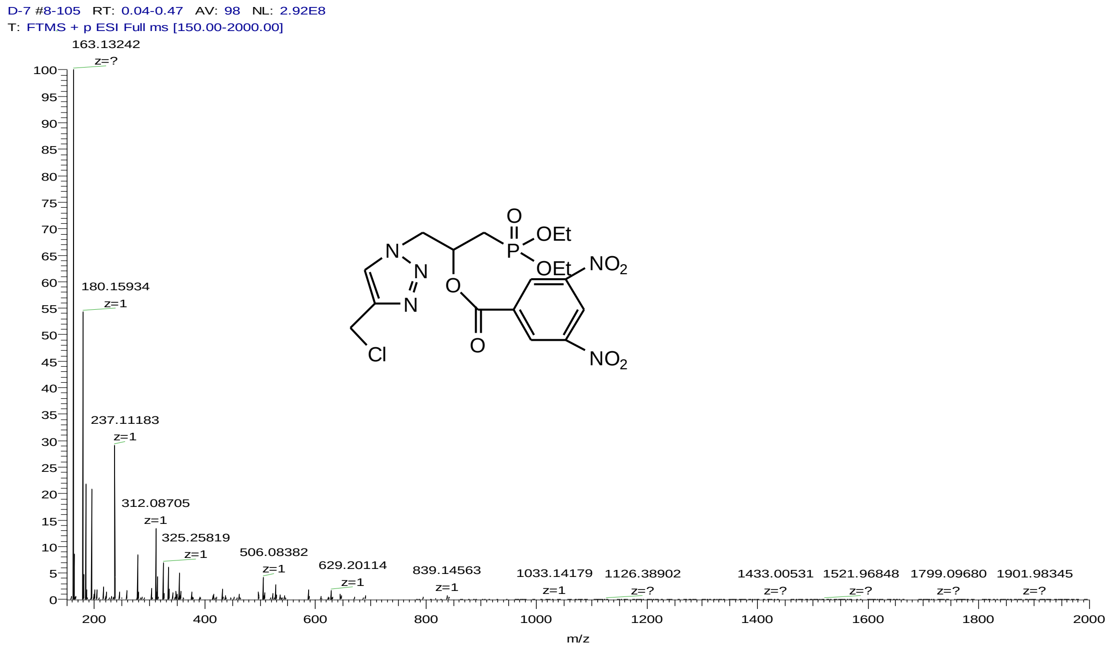

Figure S149. HRMS spectrum of compound **5-D7**.

5-D7 [M+H]506.0838179

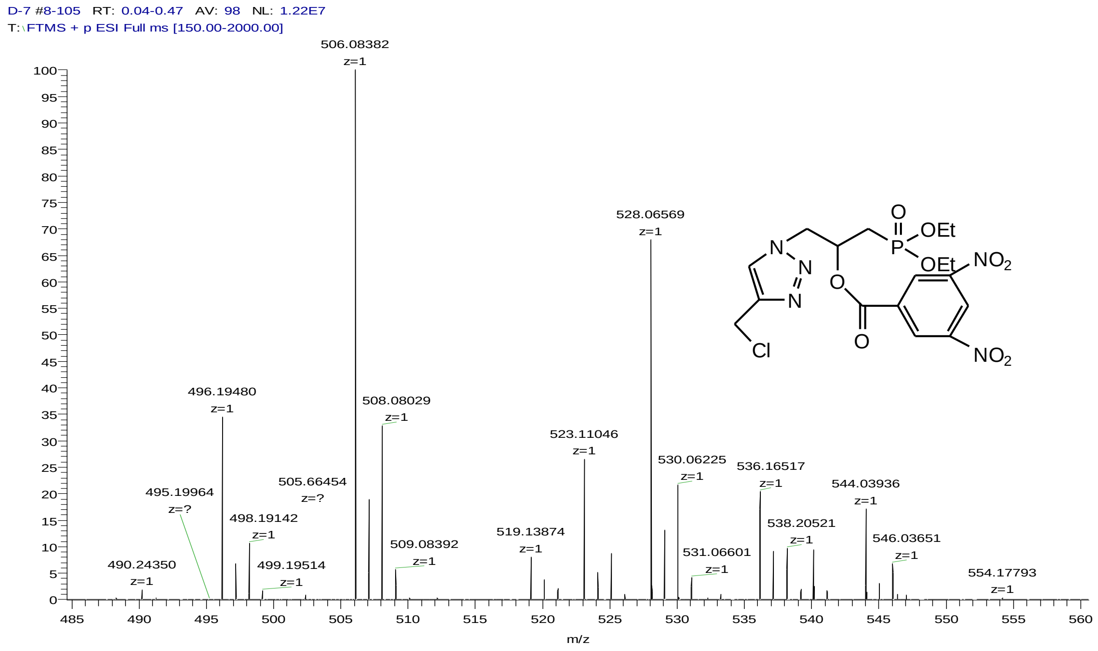

Supplement: Supplementary file 1 [file molecules-20-01088-s001.pdf]
